# Room-Temperature Superconductivity in Yb/Lu Substituted Clathrate Hexahydrides under Moderate Pressure

**DOI:** 10.34133/2022/9784309

**Published:** 2022-08-05

**Authors:** Mingyang Du, Hao Song, Zihan Zhang, Defang Duan, Tian Cui

**Affiliations:** ^1^College of Physics, Jilin University, Changchun 130012, China; ^2^Institute of High-Pressure Physics, School of Physical Science and Technology, Ningbo University, Ningbo 315211, China

## Abstract

Room temperature superconductivity is a dream that mankind has been chasing for a century. In recent years, the synthesis of H_3_S, LaH_10_, and C-S-H compounds under high pressures has gradually made that dream become a reality. But the extreme high pressure required for stabilization of hydrogen-based superconductors limit their applications. So, the next challenge is to achieve room-temperature superconductivity at significantly low pressures, even ambient pressure. In this work, we design a series of high temperature superconductors that can be stable at moderate pressures by incorporating heavy rare earth elements Yb/Lu into sodalite-like clathrate hexahydrides. In particular, the critical temperatures (*T*_c_) of Y_3_LuH_24_, YLuH_12_, and YLu_3_H_24_ can reach 283 K at 120 GPa, 275 K at 140 GPa, and 288 K at 110 GPa, respectively. Their critical temperatures are close to or have reached room temperature, and minimum stable pressures are significantly lower than that of reported room temperature superconductors. Our work provides an effective method for the rational design of low-pressure stabilized hydrogen-based superconductors with room-temperature superconductivity simultaneously and will stimulate further experimental exploration.

## 1. Introduction

Since H. K. Onnes observed the superconductivity of Hg in 1911, researchers have been trying many ways to find new superconductors or improve their superconducting properties. For example, cuprate high-temperature superconductor HgBa_2_Ca_2_Cu_3_O_8+*δ*_ with critical temperature (*T*_c_) of 133 K was discovered [1], whose *T*_c_ was improved to 164 K in diamond anvil cells [2]; the *T*_c_ of Bi_2_Sr_2_CaCu_2_O_8+*δ*_ was altered from 84 K to 94 K via a shockwave treatment [3]. Recently, many hydrides with *T*_c_s exceeding 200 K were discovered under high pressure [4–6], e.g., H_3_S with 203 K at 155 GPa [7–10] and LaH_10_ with 250-160 K at 170 GPa [11–14].

Among these hydrogen-based superconductors, clathrate hydrides are one type that exists widely, including the well-known CaH_6_ with H_24_ cage [15], YH_9_ with H_29_ cage [16], and LaH_10_ with H_32_ cage [11, 12]. The clathrate hexahydrides *Im*-*3*m-XH_6_ (*X* = Mg, Ca, Sc, Y, La, Tm, Yb, and Lu) are prevalent in alkaline earth and rare earth metal hydride [12, 15, 17–20], in which, the metal atoms form a body centered cubic (bcc) lattice, and hydrogen atoms occupy all the tetrahedral voids of the bcc lattice, forming a H_24_ cage. CaH_6_ and YH_6_ have been experimentally synthesized and exhibit high *T*_c_ of 215 K at 172 GPa and 227 K at 166 GPa, respectively [21, 22]. Theoretical predicted *T*_c_s of MgH_6_, ScH_6_, and LaH_6_ are 260 K at 300 GPa, 147 K at 285 GPa, and 174 K at 100 GPa, respectively. It is reported that the large chemical precompression of H-rich clathrate structures can be attained in rare earth hydrides with 4*f* electrons [23]. With the filling of the *f* orbitals of metal atoms, the structure is more easily stabilized at low pressure, but the unfilled *f* electrons can negatively affect superconductivity. For example, the CeH_9_ with unfilled 4*f* orbitals were experimentally synthesized at low pressure of 88 GPa with low *T*_c_ of 57 K [24]. TmH_6_, with unfilled 4*f* orbitals, is predicted to stable at 50 GPa and has a lower *T*_c_ of 25 K. YbH_6_ and LuH_6_, with filled *f*-shelled, are predicted to exhibit high-*T*_c_ superconductivity of 145 K and 273 K at relatively low pressures of 70 GPa and 100 GPa, respectively [20]. These results suggest that heavy rare earth metals Yb/Lu are suitable elements to reduce the pressure of stability and keep high *T*_c_ simultaneously.

Incorporating a new element into binary hydrides to form ternary hydrides is an important way to improve the superconducting transition temperature or reduce the superconducting phase stable pressure. In 2019, Li_2_MgH_16_ with the highest *T*_c_ to date (473 K at 250 GPa) was designed by introducing extra electrons (Li element) to fill the antibonding orbital of the H_2_ molecular units of MgH_16_ [25]. In 2020, a compound of hydrogen, carbon, and sulfur showed a superconducting transition at 288 K [26] and 267 GPa. However, the stoichiometry and crystal structure of this compound have not yet been determined. This experiment is still subject to many controversies [16, 27, 28]. Although the *T*_c_s of Li_2_MgH_16_ and C-S-H compounds can reach room temperature, the extreme pressure above 250 GPa also makes them difficult in practical application. Recently, a new class of fluorite-type clathrate ternary hydrides AXH_8_ (*A* = Ca, Sr, Y, and La, *X* = B, Be, and Al) with hydrogen alloy backbone were predicted [29]. The most outstanding one, LaBeH_8_, is dynamically stable down to 20 GPa with a high *T*_c_ ∼185 K. It is inspiring that the cubic clathrate superhydrides La_x_Y_1-x_H_6,10_ via laser heating of yttrium–lanthanum alloys have been experimental synthesized exhibiting a maximum critical temperature *T*_c_ of 253 K without increasing pressure [30]. This experiment demonstrates that selecting a suitable central metal element to substitute sodalite-like clathrate hydrides is feasible.

The focus of hydrogen-based superconductor research is not simply the pursuit of high-temperature superconductivity or low stable pressure. A good superconductor should achieve a good balance between the pressure required for stability and the critical temperature. The next challenge is to achieve room-temperature superconductivity at significantly low pressures, even ambient pressure. In the present work, we chose heavy rare earth element Yb/Lu-doped clathrate hexahydrides to achieve this goal. According to the aforementioned introduction, the heavy rare earth metals Yb/Lu show outstanding properties in sodalite-like clathrate hexahydrides YbH_6_ and LuH_6_. With full-filled *f*-shells, Yb/Lu can eliminate the negative impact on *T*_c_ of *f* electrons, meanwhile satisfying the purpose of reducing the pressure.

Here, a serial of ternary hydrides A_1-x_B_x_H_6_ (*A* = Y, Ca, Sc, *B*=Yb, Lu, and *A* = Yb, *B* = Lu, *x* = 0.25, 0.33, 0.5, 0.67, 0.75) with high *T*_c_ at moderate pressure were found, in which the atomic radii of *A* and *B* are similar. Among them, the critical temperatures of Y_3_LuH_24_, YLuH_12_, and YLu_3_H_24_ are 283 K at 120 GPa, 275 K at 140 GPa, and 288 K at 110 GPa, respectively, which are close to or have reached room temperature. Recently, it is reported that pressure-induced high-temperature superconducting FeSe retained without pressure via pressure quenching (PQ) [31]. This PQ technique offers the possibility of these high *T*_c_ hydrides for practical application in the future.

## 2. Results

**Figure 1:**
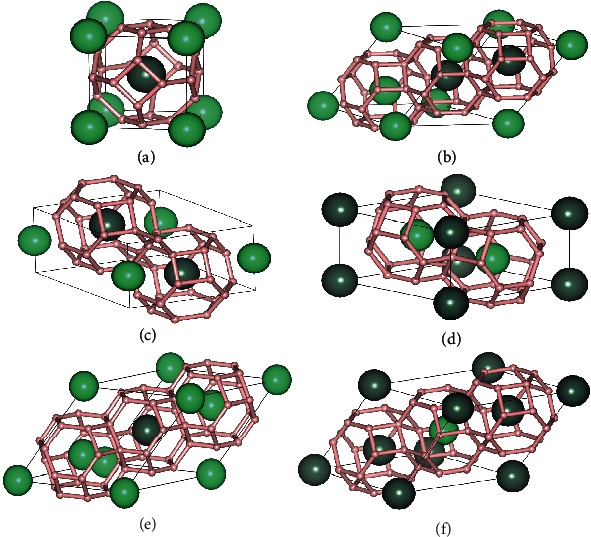
Crystal structures of (a) *Pm*-3m-ABLuH_12_, (b) *Fd*-3*m*-ABH_12_, (c) *P*-3*m*1-AB_2_H_18_, (d) *P*-3*m*1-A_2_BH_18_, (e) *Fm*-3*m*-AB_3_H_24_, and (f) *Fm*-3*m*-A_3_BH_24_. The light green and dark green balls represent “precompressor” metal atoms A and B, respectively. The small pink balls represent H atoms.

We first performed an extensive variable composition structure searches of ternary hydrides Y-Lu-H, Ca-Lu-H, and Yb-Lu-H under high pressure. Six sodalite-like clathrate structures A_1-x_B_x_H_6_ (*A* = Y, Ca, and Yb, *B* = Lu, *x* = 0.25, 0.33, 0.5, 0.67, 0.75) were found in our structure searches, including *Pm*-3m and *Fd*-3*m* of ABH_12_, *P*-3*m*1-AB_2_H_18_, *P*-3*m*1-A_2_BH_18_, *Fm*-3*m*-AB_3_H_24_, and *Fm*-3*m*-A_3_BH_24_ (see Figure 1). The thermodynamic stability of these structures was determined by constructing convex hulls (see Figures S1–S4). YLu_3_H_24_, YLuH_12_, Y_3_LuH_24_, CaLuH_12_, CaLu_3_H_24_, and Yb_2_LuH_18_ are thermodynamically stable at 300 GPa, and Ca_3_LuH_24_ can be thermodynamically stable at 200 GPa. In addition to hexahydrides, several thermodynamically stable ternary hydrides such as YLuH_8_, Y_3_LuH_20_, CaLu_3_H_3_, and Ca_3_LuH_15_ were discovered, which will not be discussed in depth in this work (see Figures S1–S4).

In order to extend the study to more ternary hydride systems, we substitute the metal elements in these six structures. The properties of clathrate structure can be further improved by choosing a suitable “precompressor” element. As discussed above, at least one of them is heavy rare earth element Yb/Lu, and the other element has similar radius with Yb/Lu, including K, Mg, Ca, Sr, Sc, Y, and La. We calculated the phonon dispersion for all possible components in the pressure range of 50-200 GPa and finally determined that 36 sodalite-like clathrate hexahydrides can be dynamically stable in seven ternary hydride systems, including Y-Lu-H, Ca-Lu-H, Sc-Lu-H, Y-Yb-H, Ca-Yb-H, Sc-Yb-H, and Yb-Lu-H (see Figures S9–S15). To determine the thermodynamic stability of these structures in Y-Yb-H, Ca-Yb-H, Sc-Lu-H, and Sc-Yb-H system, we also performed the fixed composition structure searches of A_x_B_1-x_H_6_ and constructed the convex hull, shown in Figures S5–S8. It is reported that the YH_6_, CaH_6_, and YbH_6_ are always thermodynamically stable in the pressure range of 150–300 GPa. Therefore, the energetic stabilities of the Yb-containing hexahydrides in Y-Yb-H and Ca-Yb-H system are evaluated using their formation enthalpies (Δ*H*) with respect to binary hexahydrides. Compared with Lu-containing hexahydrides, many Yb-containing hexahydrides can be thermodynamically stable at 200 GPa, including CaYbH_12_, Ca_3_YbH_24_, CaYb_3_H_24_, and YYbH_12_. All Sc-containing sodalite-like clathrate hexahydrides we studied are metastable phases in our studied pressure range (see Figures S7 and S8).

After determining the stability of all structures, we further calculated their superconducting properties. The superconductive transition temperatures of these structures are estimated through the Allen−Dynes-modified McMillan equation with correction factors and self-consistent solution of the Eliashberg equation (see Table S1). We also calculated the critical temperatures of CaH_6_ and YH_6_ in the same way and compared them with the experimentally measured values (see Table S2). The results show that the critical temperature *T*_c_ using the self-consistent solution of the Eliashberg equation is in good agreement with the experimental measurements.

**Figure 2:**
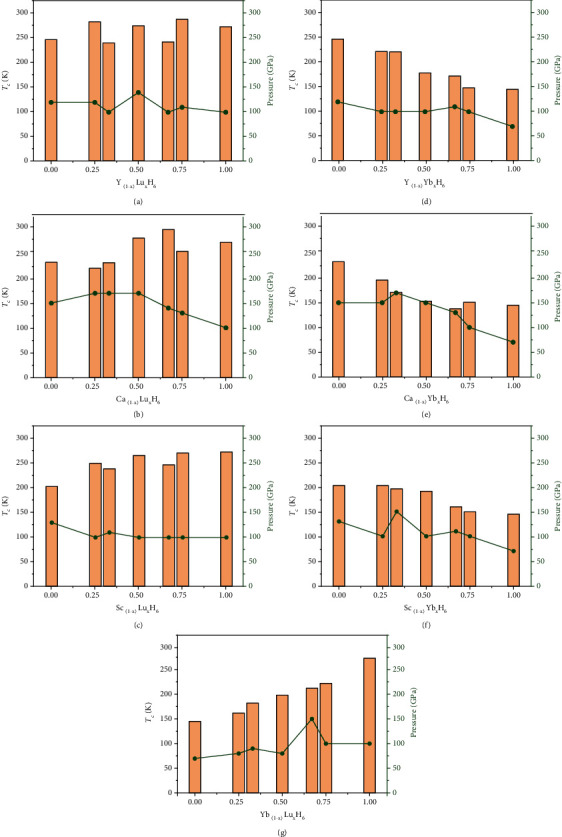
The calculated superconducting critical temperature *T*_c_ using the self-consistent solution of the Eliashberg equation and the minimum dynamically stable pressure as a function of doping concentration in (a) Y_(1-x)_Lu_x_H_6_, (b) Ca_(1-x)_Lu_x_H_6_, (c) Sc_(1-x)_Lu_x_H_6_, (d) Y_(1-x)_Yb_x_H_6_, (e) Ca_(1-x)_Yb_x_H_6_, (f) Sc_(1-x)_Yb_x_H_6_, and (g) Yb_(1-x)_Lu_x_H_6_. The Coulomb pseudopotential is using *μ*^∗^ = 0.13.

Figure 2 shows the variation of critical temperature *T*_c_ and minimum dynamically stable pressure with the concentration of heavy rare earth element Lu/Yb in sodalite-like clathrate hexahydrides. YH_6_ has more excellent properties than CaH_6_ and ScH_6_, and its properties are further improved after incorporation of Lu. The results show that YLu_2_H_18_ and Y_2_LuH_18_ with the *P*-3m1 space group have similar properties, with high *T*_c_ of 242 K and 240 K at 100 GPa, respectively (see Figure 2(a)). And YLu_3_H_24_ and Y_3_LuH_24_ with the *Fm*-3m space group also have very close properties, exhibiting room temperature of 288 K at 110 GPa and 283 K at 120 GPa, respectively. The critical temperature of YLuH_12_ with space group *Pm*-3*m* is 275 K at 140 GPa, reaching ice point temperature. The critical temperatures of Y_3_LuH_24_, YLuH_12_, and YLu_3_H_24_ are close to or have reached room temperature. Surprisingly, the images of *T*_c_ and minimum pressure of Y_(1-x)_Lu_x_H_6_ exhibit mirror symmetry as a function of Lu doping concentration due to their similar properties (see Figure 2(a)). For Y_(1-x)_Lu_x_H_6_, the structure appears to have a greater effect on the *T*_c_ and minimum pressure than the doping concentration.

For CaH_6_, both *T*_c_ and minimum pressure show a trend of first increasing and then decreasing with the Lu doping concentration (see Figure 2(b)). Ca_3_LuH_24_ has a *T*_c_ of 221 K at 170 GPa, which is close to CaH_6_. At 170 GPa, the critical temperature of CaLuH_12_ is 282 K, near room temperature. And CaLu_2_H_18_ can be dynamically stable at 140 GPa, and its *T*_c_ is as high as 299 K at this pressure, which is the highest *T*_c_ in this work. It is only 6 meV/atom above the convex hull at 300 GPa, implying the possibility of experimental synthesis. For ScH_6_, doping with Lu can significantly reduce the minimum stable pressure and increase *T*_c_. With the increase of Lu doping concentration in Sc_(1-x)_Lu_x_H_6_, *T*_c_ showed an upward trend, and the minimum pressure is basically maintained at about 100 GPa (see Figure 2(c)). The effect of Lu on reducing the minimum pressure is obvious. The most prominent is ScLu_3_H_24_, which reaches a *T*_c_ of 271 K at 100 GPa, close to ice point temperature. In short, doping Lu in binary hexahydrides achieves the goal of reducing the dynamical stable pressure and increasing *T*_c_, such as YLuH_12_ (275 K at 140 GPa), YLu_3_H_24_ (288 K at 110 GPa), Y_3_LuH_24_ (283 K at 120 GPa), CaLuH_12_ (282 K at 170 GPa), and CaLu_2_H_18_ (299 K at 140 GPa).

The *T*_c_s of Yb-containing structures are all significantly lower than those of Lu-containing structures and show a decreasing trend with the increase of doping concentration of Yb. There are only five low Yb concentration hydrides have *T*_c_ over 200 K, such as Y_3_YbH_24_ (222 K at 100 GPa), Y_2_YbH_18_ (221 K at 100 GPa), Sc_3_YbH_24_ (203 K at 100 GPa), YbLu_2_H_18_ (212 K at 150 GPa), and YbLu_3_H_24_ (222 K at 100 GPa). The effect of Yb on reducing the dynamical stable pressure is obvious. The minimum dynamically stable pressure of Y_(1-x)_Yb_x_H_6_ is basically maintained around 100 GPa (see Figure 2(d)). But for Ca_(1-x)_Yb_x_H_6_, the introduction of heavy rare earth element cannot always reduce the dynamically stable pressure (see Figure 2(e)). The minimum dynamically stable pressure of CaYbH_12_ and Ca_3_YbH_24_ is 150 GPa like that of CaH_6_, and the minimum dynamically stable pressure of Ca_2_YbH_18_ is even higher than that of CaH_6_, reaching 170 GPa. Only CaYb_2_H_18_ and CaYb_3_H_24_ with Yb concentrations over 50% exhibit lower pressures than CaH_6_, with minimum dynamically stable pressures of 130 GPa and 100 GPa, respectively. This is similar to Ca_(1-x)_Lu_x_H_6_ discussed above. Doping less than 50% Yb/Lu in CaH_6_ cannot reduce the dynamically stable pressure. This is due to the large difference between the radius of Ca and Yb/Lu. The minimum pressures of Sc_(1-x)_Yb_x_H_6_ and Yb_(1-x)_Lu_x_H_6_ decrease with the concentration of Yb doping (see Figures 2(f) and 2(g)). Especially YbLuH_12_ and Yb_3_LuH_24_, which contain both heavy rare earth elements Yb and Lu, can be stable at 80 GPa and exhibit *T*_c_ of 198 K and 162 K, respectively. They have the lowest stable pressure in this work. To confirm the effect of 4*f* electrons on strengthening precompression, we calculated the phonon spectrum of YbLuH_12_ at 80 GPa without considering the interactions of 4*f* electrons in valence electrons (see Figure S15). The calculated phonon spectrum exhibits imaginary frequencies in the whole Brillouin zone, indicating that YbLuH_12_ is dynamically unstable. This result suggests that 4*f* electrons play an important role in stabilizing these clathrate structures.

The focus of hydrogen-based superconductor research is not simply the pursuit of high-temperature superconductivity, after the realization of room-temperature superconductivity. A good superconductor should achieve a good balance between the pressure required for stability and the critical temperature. Therefore, we use a figure of merit *S* [38] to evaluate the significance of all thermodynamically stable sodalite-like clathrate hexahydrides and metastable phase CaLu_2_H_18_ in this work. *S* is obtained from the critical temperature *T*_c_ and the pressure required for stabilization *P*:

(1) $$\begin{array}{c} S=\frac{T_{c}}{\sqrt{T_{c,{MgB}_{2}}^{2}+P^{2}}}. \end{array}$$

**Figure 3:**
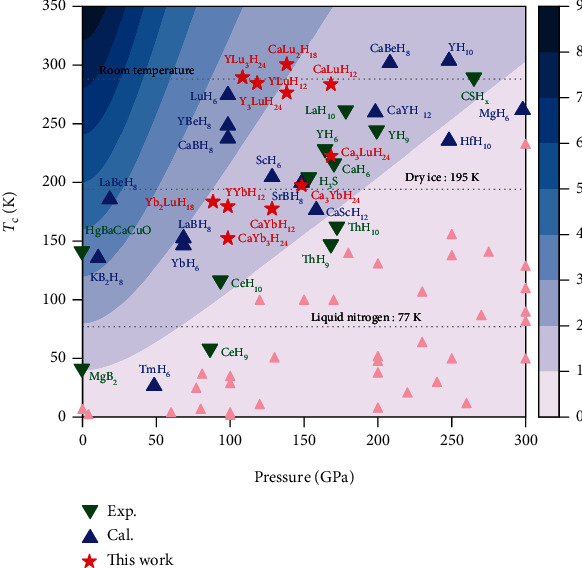
Pressure dependence of *T*_c_s calculated for Yb/Lu substituted hexahydrides shown alongside other high-*T*_c_ superconductors. All thermodynamically stable hexahydrides and metastable phase CaLu_2_H_18_ in this work are marked with red stars. Blue triangles correspond to theoretical predictions [11, 17, 19, 20, 29, 32–34], green inverted triangles correspond to experimental measurements [1, 9, 14, 21, 22, 24, 26, 35–37], and pink triangles correspond to other less prominent results. The background is shaded according to the figure of merit *S* [38].

Y_3_LuH_24_ and YLu_3_H_24_ with *S* > 2 and YLuH_12_ and Yb_2_LuH_18_ with *S* close to 2 are better than the well-known hydrogen-based superconductor H_3_S, LaH_10_, and C-S-H compounds. Most importantly, it can be seen from Figure 3 that Y_3_LuH_24_ is the room temperature superconductor with the lowest pressure required for stability (110 GPa), which is much lower than that of C-S-H, YH_10_ and CaBeH_8_ with room-temperature superconducting. It also means that room-temperature superconductivity at moderate pressure is promising in hydrogen-based superconductors.

**Figure 4:**
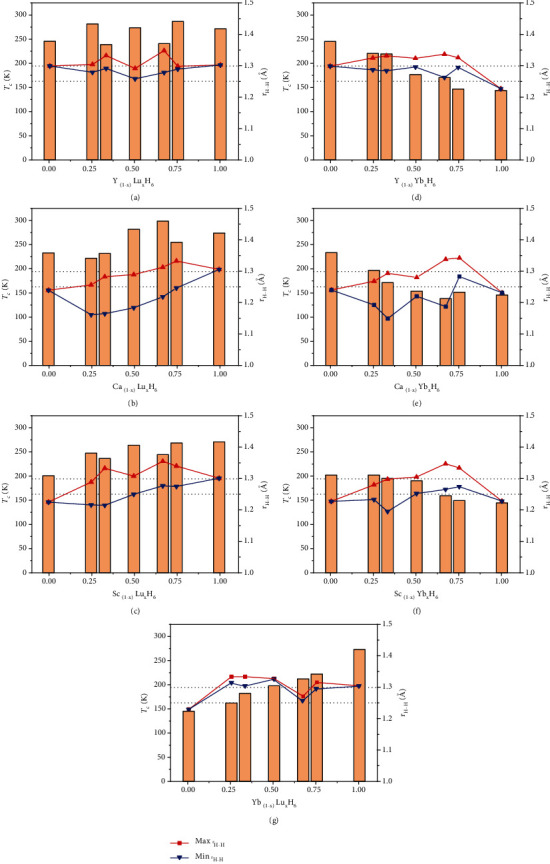
Maximum and minimum H-H bond lengths and calculated critical temperature *T*_c_s as a function of doping concentration in (a) Y_(1-x)_Lu_x_H_6_, (b) Ca_(1-x)_Lu_x_H_6_, (c) Sc_(1-x)_Lu_x_H_6_, (d) Y_(1-x)_Yb_x_H_6_, (e) Ca_(1-x)_Yb_x_H_6_, (f) Sc_(1-x)_Yb_x_H_6_, and (g) Yb_(1-x)_Lu_x_H_6_. Dashed lines correspond to bond lengths of 1.25 and 1.3 Å.

It seems difficult to summarize the law of *T*_c_ change only from the perspective of doping concentration for Lu-containing structures. So, we evaluated the distribution of H-H bond lengths for all hexahydrides in this work.  Figure 4 shows the variation of superconducting critical temperature *T*_c_ and H-H bond lengths with the concentration of heavy rare earth element Lu/Yb in sodalite-like clathrate hexahydrides. There are nine hexahydrides with *T*_c_ over 250 K, including LuH_6_, YLuH_12_, YLu_3_H_24_, Y_3_LuH_24_, CaLuH_12_, CaLu_2_H_18_, CaLu_3_H_24_, ScLuH_12_, and ScLu_3_H_24_. They all have one thing in common, that is, the H-H bond lengths are all distributed around 1.25-1.30 Å (see Figures 4(a)–4(c)). The H-H bond length distribution may be an important factor on *T*_c_ for Lu-containing structures. The more H-H bond lengths are distributed around 1.25-1.30 Å, the higher *T*_c_ will be. Elongating the H-H bond length to more than 1.25 Å is an effective means to increase *T*_c_. In the Yb-containing hexahydrides, Y_3_YbH_24_, Y_2_YbH_18_, Sc_3_YbH_24_, YbLu_2_H_18_, and YbLu_3_H_24_ have *T*_c_ higher than 200 K, in which the H-H bond length distribution is also close to 1.25-1.30 Å (see Figures 4(d)–4(g)), compared to other Yb-containing hexahydrides. This means that the H-H bond length distribution is likely to be an important reference for finding high-temperature hydrogen-based superconductors in these sodalite-like clathrate hexahydrides.

**Figure 5:**
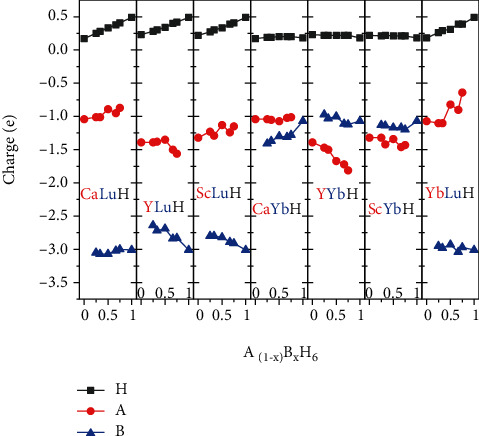
Charges transferred as a function of doping concentration between different elements in A_(1-x)_B_x_H_6_. Negative means loss of electrons; positive means gain of electrons.

Charge transfer is essential for the formation of hydrogen cages. The stability of H_24_ cages in clathrate hexahydrides comes from H_2_ molecular units accepting electrons from the central metal atoms to form six H_4_ units as the cornerstone of the construction of the three-dimensional sodalite gabion.  Figure 5 shows the charge transfer between two “precompressor” metal elements and hydrogen in all sodalite-like clathrate hexahydrides we studied. It can be clearly seen that Lu element is an extremely good electron donor. Each Lu atom can donate 3.07 electrons at most, which is far more than other metal elements. The electrons obtained by H atom in the Lu-containing structures increase with increasing the Lu doping concentration (black curves). In the structures without Lu, the number of electrons obtained by H atom is basically the same level (about 0.23 |*e*|). Although Y atom is also ideal candidates for “precompressor” metal element, it can only donate up to 1.81 electrons. Ca, Sc, and Yb atoms cannot donate more than 1.5 electrons. Both YH_6_ and CaH_6_ exhibit high-temperature superconductivity, but they are still not qualified for room temperature superconductor. The introduction of Lu makes it possible to achieve room-temperature superconductivity in sodalite-like clathrate hexahydrides.

## 3. Discussion

**Figure 6:**
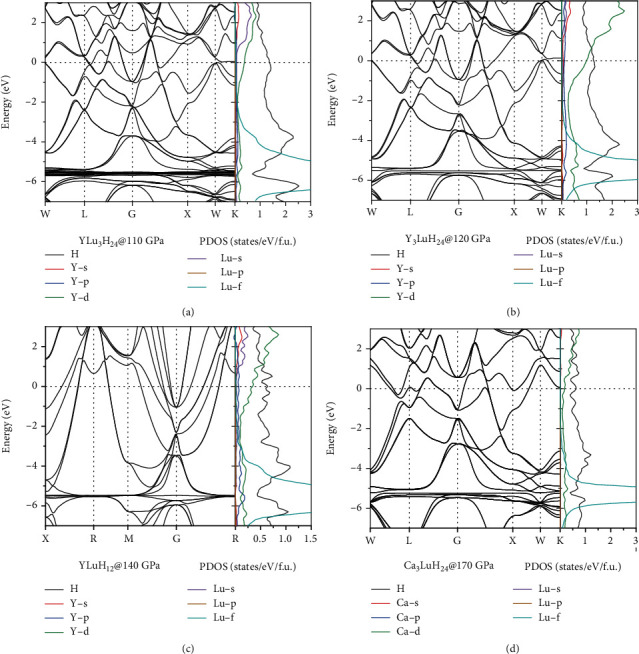
Electronic band structures and projected density of electronic states (PDOS) of (a) *Fm*-3m-YLu_3_H_24_ at 110 GPa, (b) *Fm*-3*m*-Y_3_LuH_24_ at 120 GPa, (c) *Pm*-3*m*-YLuH_12_ at 140 GPa, and (d) *Fm*-3*m*-Ca_3_LuH_24_ at 170 GPa.

According to the aforementioned findings, the critical temperatures of Y_3_LuH_24_, YLuH_12_, and YLu_3_H_24_ have reached room temperature under moderate pressure. In addition, although the *T*_c_ of Ca_3_LuH_24_ is only 221 K at 170 GPa, it has a lower thermodynamically stable pressure (below 200 GPa), meaning that it is easier to be synthesized experimentally. To gain insight into the origin of room-temperature superconductivity for these sodalite-like clathrate hexahydrides, we calculated their electronic band structures and projected density of electronic states (PDOS), as shown in Figure 6. For hydrides, the contribution of electronic states of H to the Fermi level is an important basis for judging whether it is an excellent superconductor. In this work, for two structures of the same space group and the same “precompressor” metal elements A and B, their contributions of electronic states of H near the Fermi surface are basically the same. For example, the contributions of electronic states of H near the Fermi surface in YLu_3_H_24_ and Y_3_LuH_24_ are both 1.5 states/eV/f.u. (see Figures 6(a) and 6(b)), which is the essential reason for their close *T*_c_.

In addition to H, *f* electrons also have an important impact on superconductivity and dynamical stability, especially in Yb-containing structures. Note that the 4*f* orbitals associated with the Yb atom form a set of localized bands that appear about 1.5 eV below the Fermi level (see Figures S19–S22). As the Yb doping concentration increases, the position of PDOS peak corresponding to the *f* electrons is always 1.5 eV below the Fermi level and does not shift, but the contribution of electronic states of the *f* electrons at the Fermi surface increases. The 4*f*-orbital electrons are good for stabilizing the structure, but excessive *f* electrons at the Fermi surface will negatively affect superconductivity. Therefore, the *T*_c_s of Yb-containing structures in Table S1 mostly does not exceed 200 K. Although lowering the concentration of Yb can increase *T*_c_, it also makes Yb lose its role in reducing the pressure required for stabilization. For Lu-containing structures, extra electron in the 5*d* orbitals leads to the 4*f* electrons moving away from the Fermi surface in the band structure (see Figures S19–S22). Thus, Lu-containing structures are not negatively affected by the 4*f*-orbital electrons, and exhibits a rather high *T*_c_.

Meanwhile, YLu_3_H_24_ can exhibit 288 K room temperature superconductivity which is also related to the flat band near the Fermi surface along the *W*-*K* direction, as shown in Figures 6(a) and 6(b). Such flat bands exist in all sodalite-like clathrate hexahydrides with *Fm*-3m space group in this work, but not all the flat bands can be near the Fermi surface. The energy bands of hydrides are influenced by the precompression element. Compared to CaH_6_, YH_6_ has a higher Fermi energy due to more valence electrons [19]. Ca_3_LuH_24_ also has the same flat band, but at 1.7 eV above the Fermi energy (see Figure 6(d)). This results in that the contribution of electronic states of H in Ca_3_LuH_24_ at the Fermi level is not as high as that in YLu_3_H_24_. It may be one of the reasons why the *T*_c_ of Ca_3_LuH_24_ is not as high as that of YLu_3_H_24_. Due to the lower Lu content, the flat band of Y_3_LuH_24_ is slightly above the Fermi surface compared to YLu_3_H_24_ (see Figure 6(b)). Among all the “precompressor” metals, Y and Lu are more favorable for high-temperaturesuperconductivity of H_24_ cage. In ternary clathrate hexahydrides A_1-x_B_x_H_6_, by selecting the appropriate “precompressor” elements A and B, adjust their proportions, maximizing *T*_c_ can be achieved.

**Figure 7:**
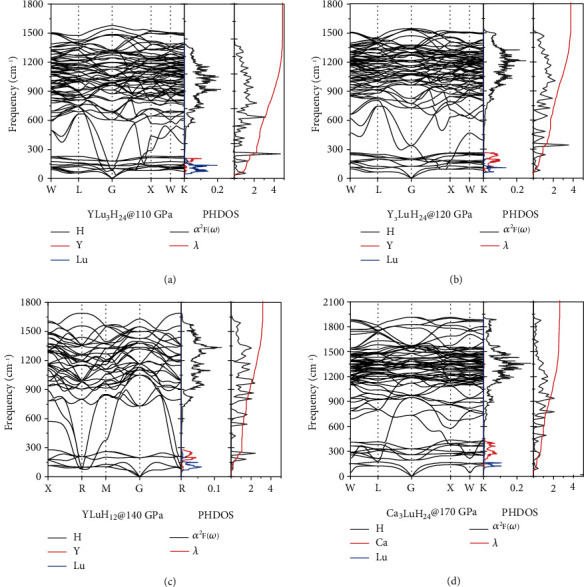
Phonon dispersion, phonon density of state (PHDOS), spectral function *α*^2^*F*(*ω*) and integral EPC parameter *λ* of (a) *Fm*-3m-YLu_3_H_24_ at 110 GPa, (b) *Fm*-3*m*-Y_3_LuH_24_ at 120 GPa, (c) *Pm*-3*m*-YLuH_12_ at 140 GPa, and (d) *Fm*-3*m*-Ca_3_LuH_24_ at 170 GPa.

The high *T*_c_s of hydrogen-based superconductors are largely due to strong electron-phonon coupling (EPC) from high frequency optical phonons. We calculated phonon spectrum, phonon density of state (PHDOS) and integral EPC parameter *λ* of all sodalite-like clathrate hexahydrides we studied, to explore the source of this strong coupling from optical phonons. As shown in Figure 7, the phonon spectra of these ternary clathrate structures are similar to those of the binary clathrate hexahydrides [15, 18]. The low frequency region is mainly the vibration of metal atoms (red and blue peak in PHDOS), and the high frequency region comes from the vibration of hydrogen (black peak in PHDOS). The maximum vibrational frequency of the hydrogen atom is related to the length of the H-H bond. The shorter the H-H bond, the higher the corresponding vibrational frequency. The maximum vibrational frequency of H_2_ or H_3_ units is generally above 2000 cm^−1^ [39]. The absence of spectral lines over 2000 cm^−1^ means that there are no H_2_ or H_3_ units. It can be seen from Figure 7 that the integral curve of *λ* (red curve) grows rapidly in 500-1500 cm^−1^, while *λ* grows slowly above 1500 cm^−1^ (see Figure 7(d)). It means that the vibration in frequency range of 500-1500 cm^−1^ is the most important source of electron-phonon coupling. By comparing the phonon spectrum (see Figures S9–S15) and bond length (see Figure 4) of all sodalite-like clathrate hexahydrides, we find that the H-H bond length corresponding to this vibrational frequency range is about 1.25-1.30 Å, which is consistent with the most suitable H-H bond length summarized in Figure 4. Elongating the H-H bond to 1.25-1.30 Å can reduce the vibrational frequency of hydrogen from high frequency (above 1500 cm^−1^) to a suitable frequency of 500-1500 cm^−1^, thereby increasing *T*_c_.

In addition to the high frequency optical phonons, “soft mode” also plays an important role in enhancement of superconductivity [40, 41]. It can be seen from YLu_3_H_24_ (Figure 7(a)) that there are some soft phonon modes near the Gamma point in frequency range of 100-500 cm^−1^. The integral curve of *λ* (red curve) grows rapidly in this frequency range which can reach 3 up to 500 cm^−1^. The Y_3_LuH_24_ and YLuH_12_ also have soft phonon modes (see Figures 7(b) and 7(c)). The integral curve of *λ* corresponding to the frequency at which the phonon softening is also rising rapidly. These results indicate that in YLu_3_H_24_, Y_3_LuH_24_, and YLuH_12_ near lattice instability, the phonon softening strengthens the electron-phonon coupling *λ*, which in turn leads to high *T*_c_.

In conclusion, the incorporation of heavy rare earth elements Yb/Lu is an effective method to tune the superconducting transition temperature and the required pressure for stabilization of sodalite-like clathrate hydrides. In particular, the introduction of Lu element can further improve the superconductivity and keep the low-pressure stability. The three most prominent compounds Y_3_LuH_24_ (283 K at 120 GPa), YLuH_12_ (275 K 140 GPa), and YLu_3_H_24_ (288 K at 110 GPa) exhibit room temperature superconductivity at much lower pressure than that of previously discovered room temperature superconductors, such as C-S-H, YH_10_, and CaBeH_8_. The enhancement of *T*_c_ is achieved by adjusting the H-H bond length to affect the hydrogen vibrational frequency and thereby enhance the electron-phonon coupling. Our results represent an important step towards room-temperature superconductivity at ambient pressure and will stimulate further experimental exploration.

## 4. Computational Methods

High-pressure structure searches were performed using the *ab initio* random structure searching (AIRSS) technique [42, 43]. For Ca-Lu-H, Y-Lu-H, and Yb-Lu-H systems, we predicted more than 8000 structures using variable composition structure searches in each system for their ternary convex hulls. Furthermore, for 36 A_1-x_B_x_H_6_ (*A* = Ca, Y, Sc, *B*=Yb, Lu, and *A* = Yb, *B* = Lu, *x* = 0.25, 0.33, 0.5, 0.67, 0.75), we predicted about 500 structures for each composition. Structure relaxations during structure searches were performed using the *ab initio* calculation of the Cambridge Serial Total Energy Package (CASTEP) code [44]. The generalized gradient approximation with the Perdew-Burke-Ernzerhof parametrization [45] for the exchange-correlation functional and ultrasoft pseudopotentials with cut-off energy of 400 eV and Brillouin zone sampling grid spacing of 2*π* × 0.07 Å^−1^ were chosen for the structure searching.

Considering the results of pseudopotential detection for Yb-H and Lu-H in previous work [20], we used CASTEP with ultrasoft pseudopotentials for structural relaxation and calculations of enthalpies and electronic properties. A cut-off energy of 1000 eV and a Brillouin zone sampling grid spacing of 2*π* × 0.03 Å^−1^ were used. All enthalpy calculations are well converged to less than 1 meV per atom, which is acceptable for density functional theoretical calculations. The charge transfer calculations are obtained using Mulliken population analysis [46].

The Quantum-ESPRESSO package [47] was used in phonon and electron−phonon calculations. Ultrasoft pseudopotentials were used with a kinetic energy cut-off of 90 Ry. The *k*-point and *q*-point meshes in the first Brillouin zone of 12 × 12 × 12 and 4 × 4 × 4 grids were adopted, respectively. The superconductive transition temperatures are estimated through the Allen−Dynes-modified McMillan equation (A-D-M) [48] with correction factors and self-consistent solution of the Eliashberg equation (scE) [49] with the Coulomb pseudopotential *μ*^∗^ = 0.10 and 0.13.

## Data Availability

The data supporting the findings of this study are available within the article and its Supplementary Materials files and from the corresponding author upon reasonable request.
