# Supplementary material for: Room-Temperature Superconductivity in Yb/Lu Substituted Clathrate Hexahydrides under Moderate Pressure
Source: Research (Wash D C). 2022 Aug 5;2022:9784309. doi: 10.34133/2022/9784309 (PMC9394054; doi:10.34133/2022/9784309)
Supplement: Supplementary Materials — Supplementary information is available and includes convex hull, phonon spectrum, band structures, superconductivity properties, and lattice parameters. [file 9784309.f1.docx]

**Supplementary Material**

**Room-temperature superconductivity in Yb/Lu substituted clathrate hexahydrides under moderate pressure**

Mingyang Du^1^, Hao Song^1^, Zihan Zhang^1^, Defang Duan^1^*, Tian Cui^1,2^*

*^1^State Key Laboratory of Superhard Materials, College of Physics, Jilin University, Changchun 130012, People's Republic of China*

*^2^School of Physical Science and Technology, Ningbo University, Ningbo, 315211, People's Republic of China*

Correspondence author: *cuitian@jlu.edu.cn, ^†^duandf@jlu.edu.cn

# FIGURES


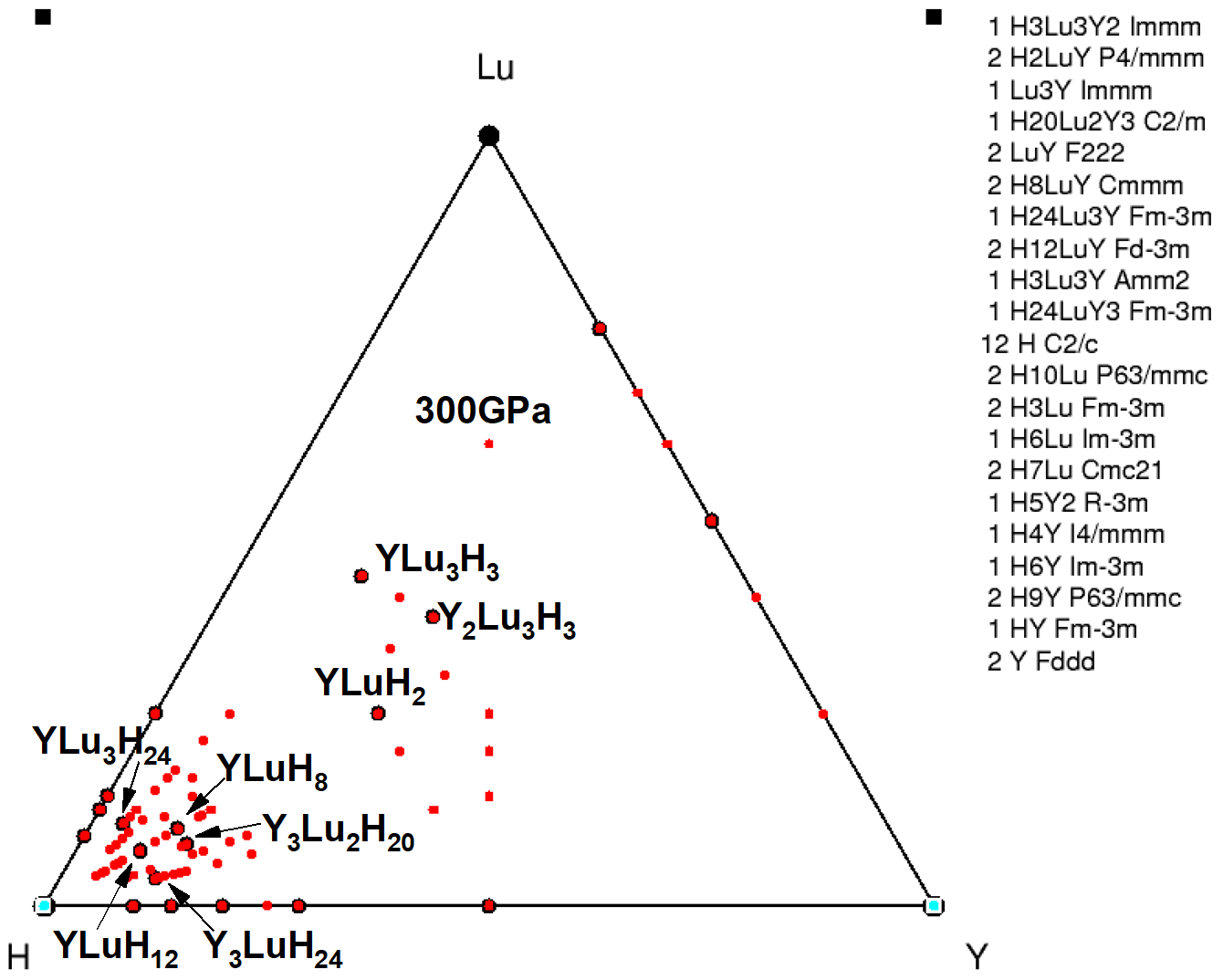


Fig. S1 The convex hull of Y-Lu-H system at 300 GPa. The corresponding elements and boundary binary phases are chosen from the results of the previous works^[1-3]^.

**
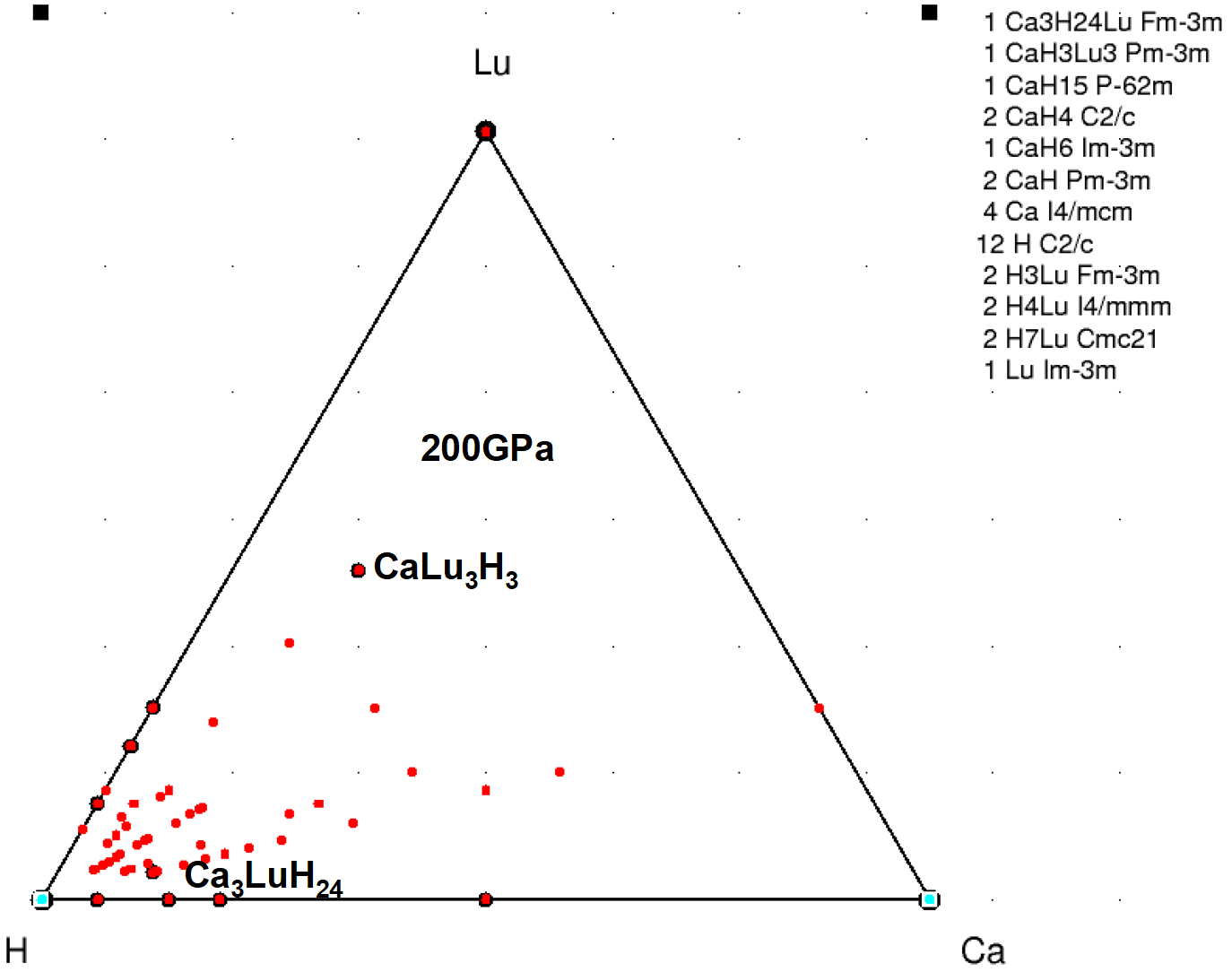
**

Fig. S2 The convex hull of Ca-Lu-H system at 200 GPa. The corresponding elements and boundary binary phases are chosen from the results of the previous works^[3, 4]^.


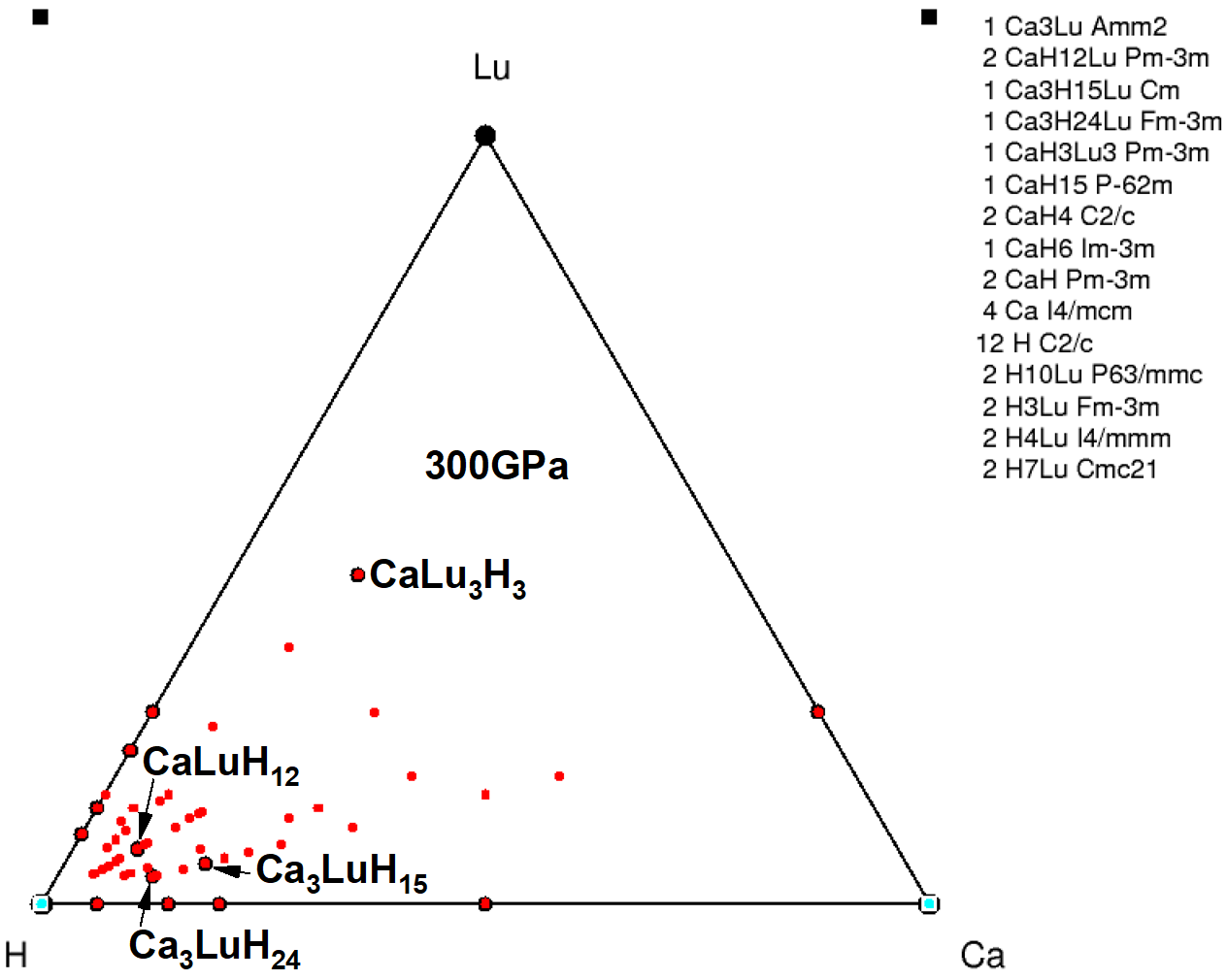


Fig. S3 The convex hull of Ca-Lu-H system at 300 GPa. The corresponding elements and boundary binary phases are chosen from the results of the previous works^[3, 4]^.


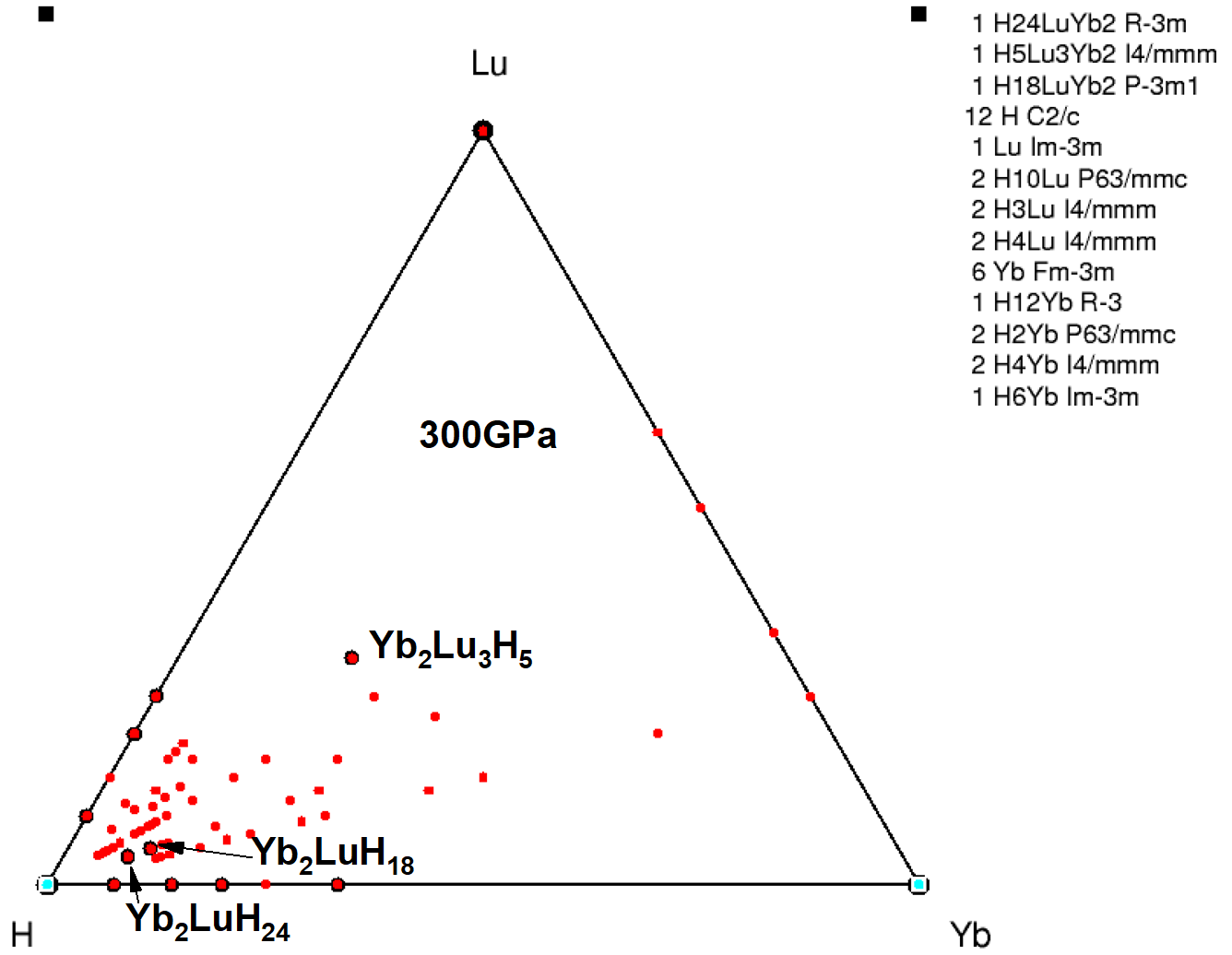


Fig. S4 The convex hull of Yb-Lu-H system at 300 GPa. The corresponding elements and boundary binary phases are chosen from the results of the previous works^[3]^.


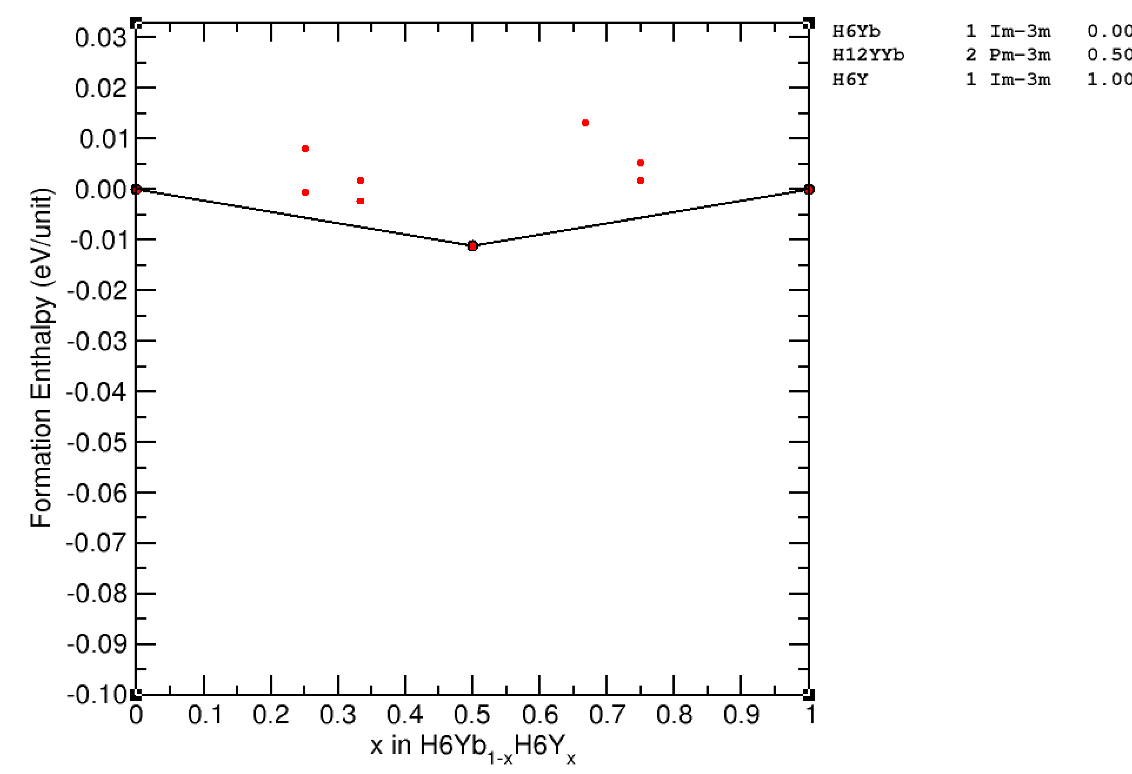


Fig. S5 The convex hull of Y-Yb-H system at 200 GPa. The corresponding boundary binary phases are chosen from the results of the previous works^[1, 3]^.


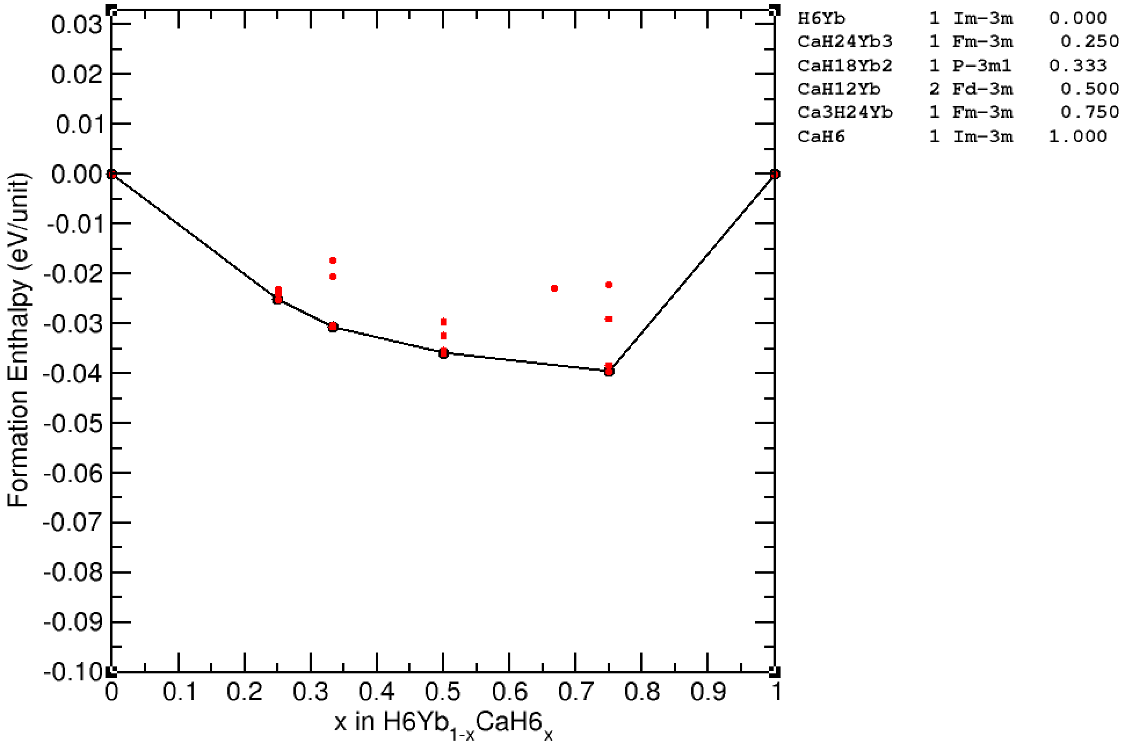


Fig. S6 The convex hull of Ca-Yb-H system at 200 GPa. The corresponding boundary binary phases are chosen from the results of the previous works^[3, 4]^.


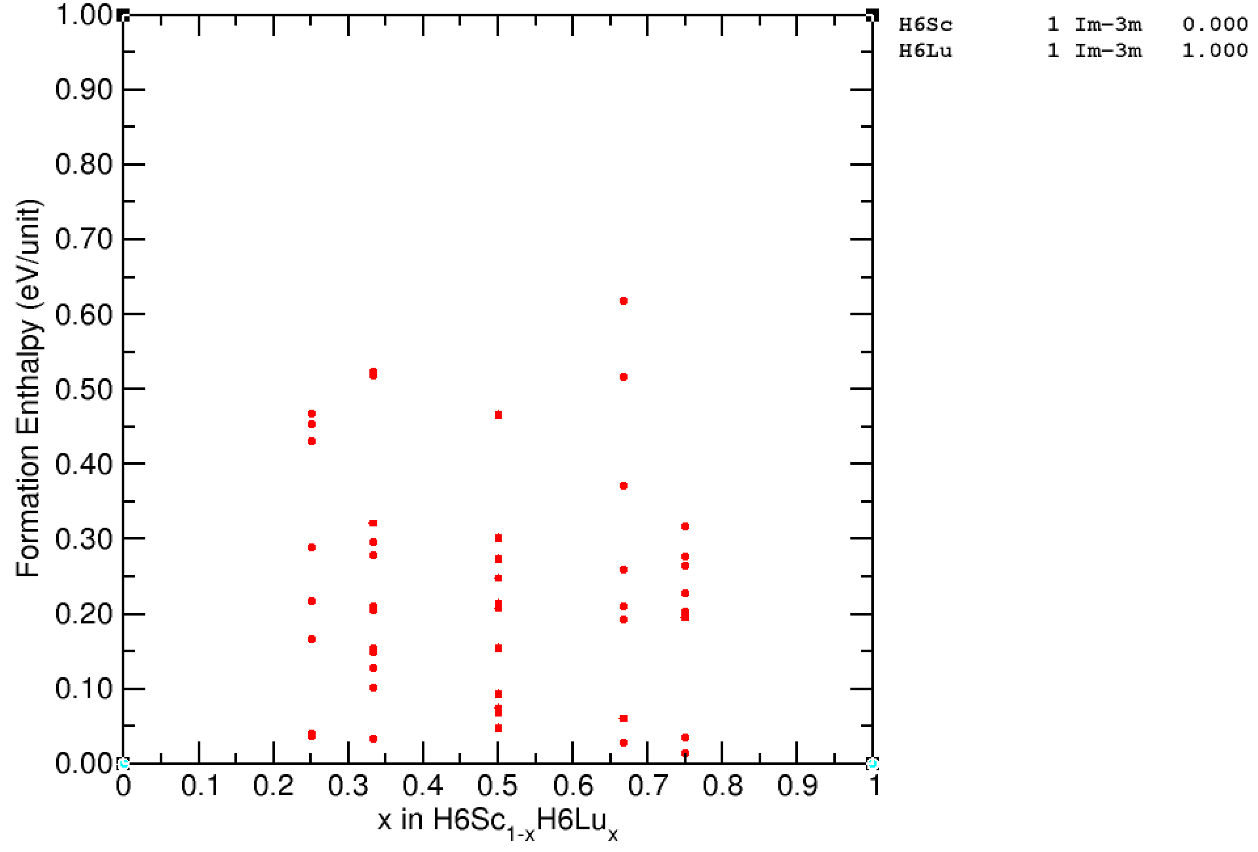


Fig. S7 The convex hull of Sc-Lu-H system at 300 GPa. The corresponding boundary binary phases are chosen from the results of the previous works ^[3, 5]^.


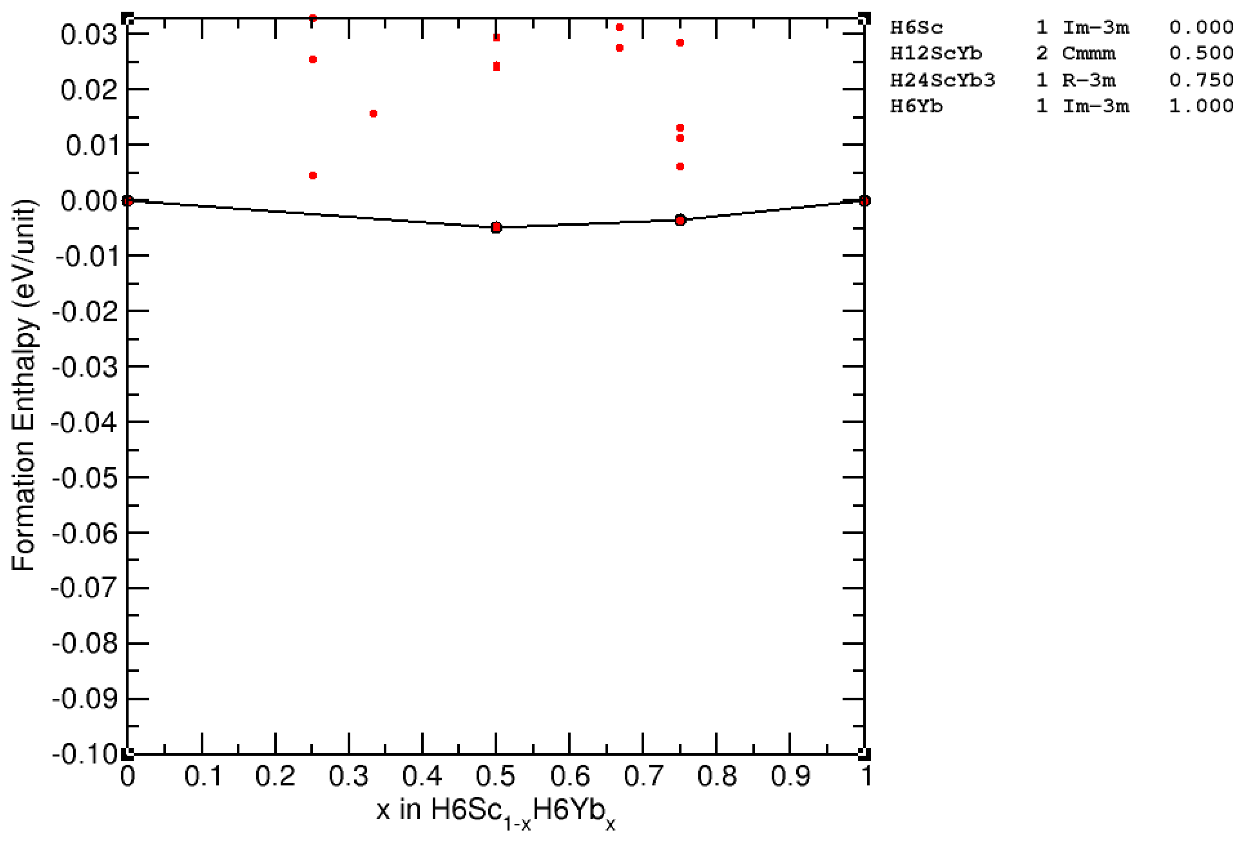


Fig. S8 The convex hull of Sc-Yb-H system at 300 GPa. The corresponding boundary binary phases are chosen from the results of the previous works ^[3, 5]^.


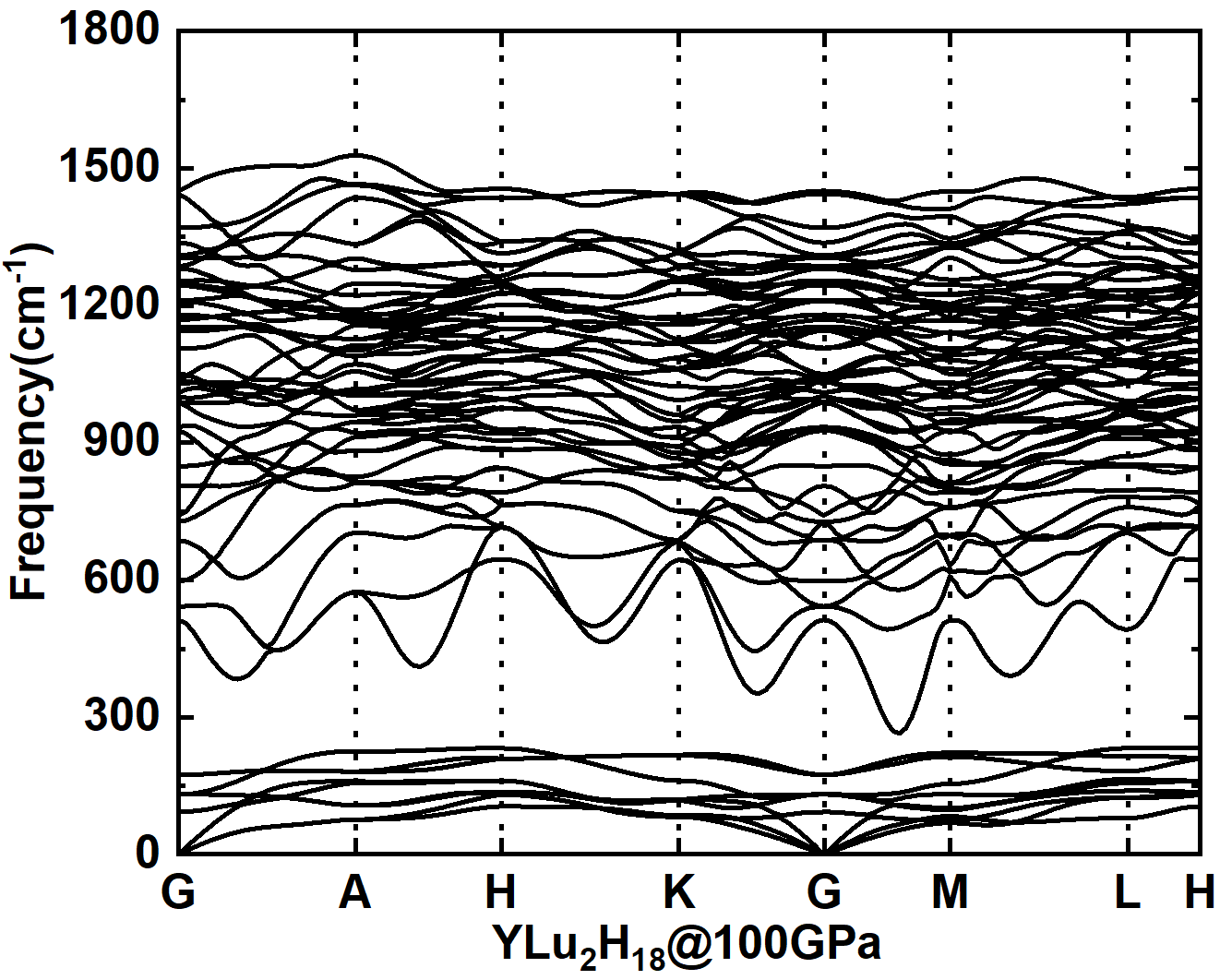

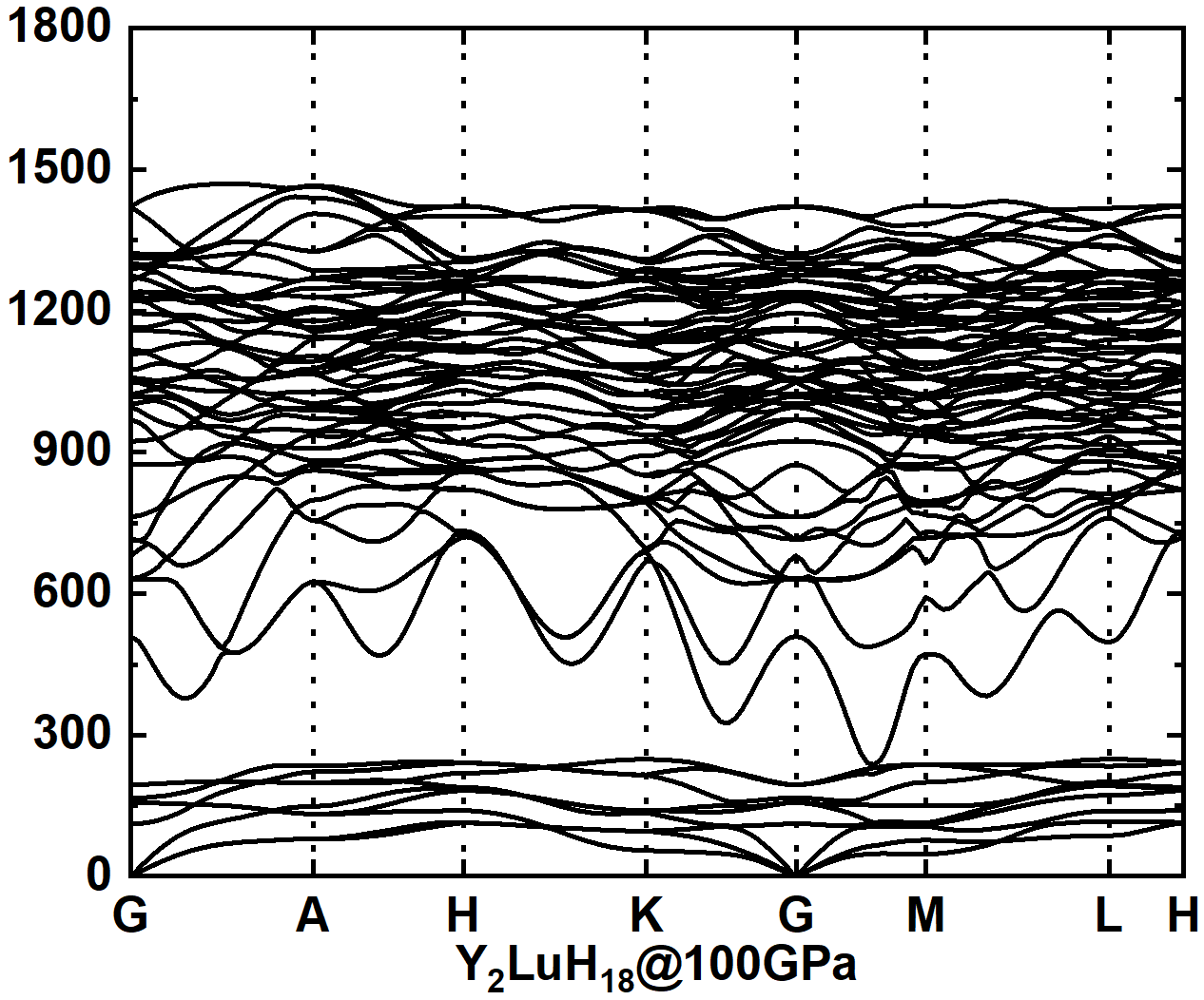


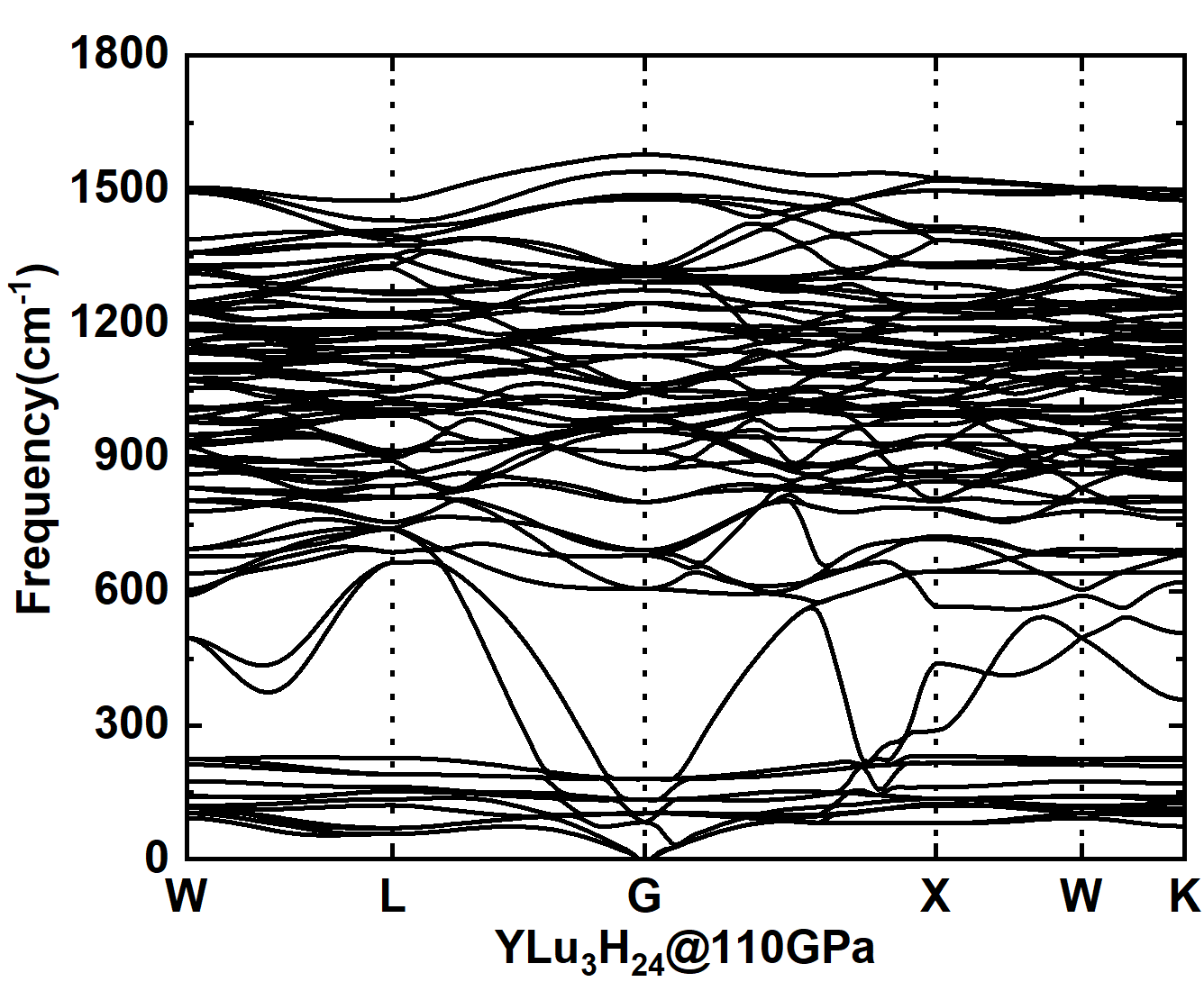

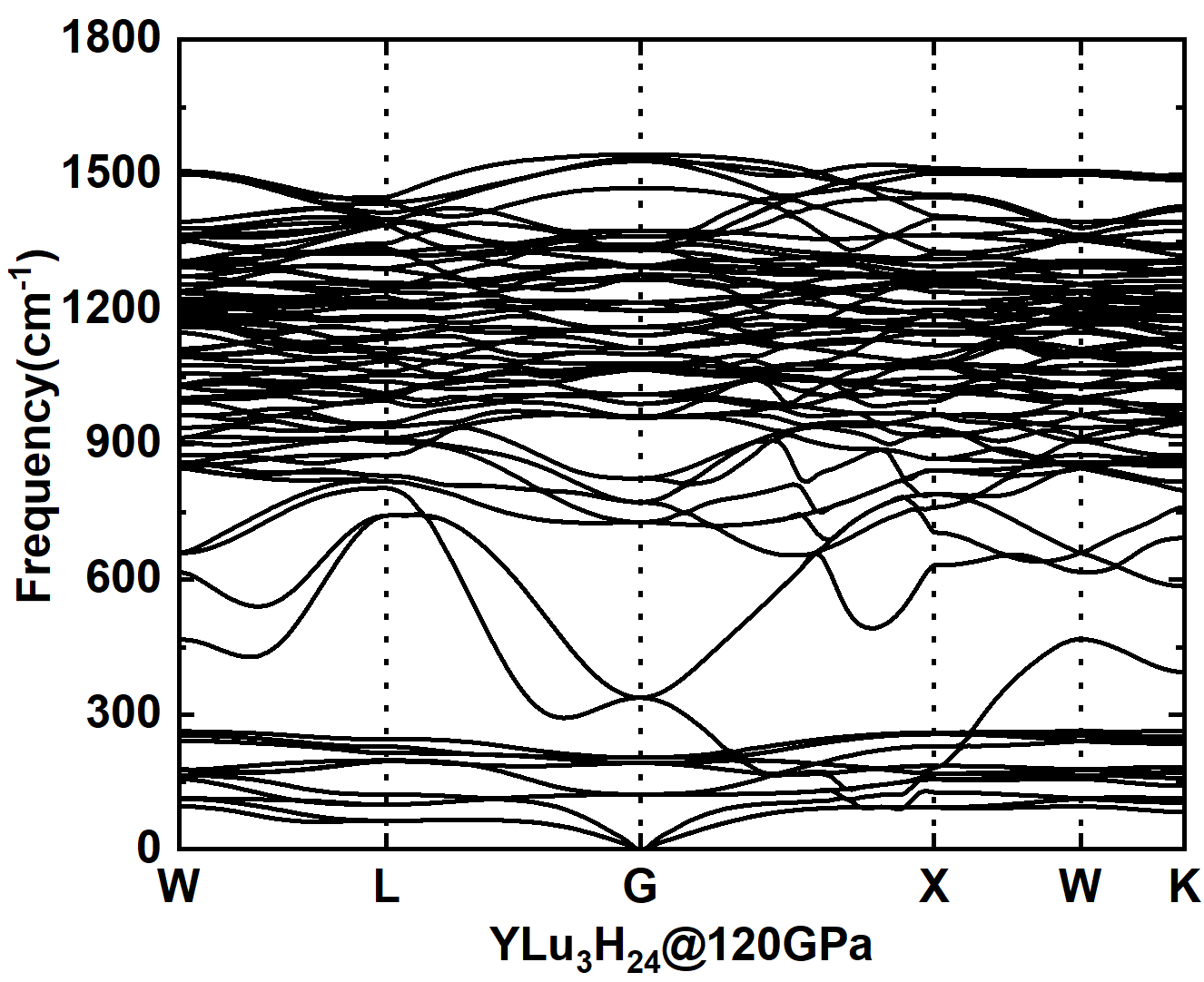


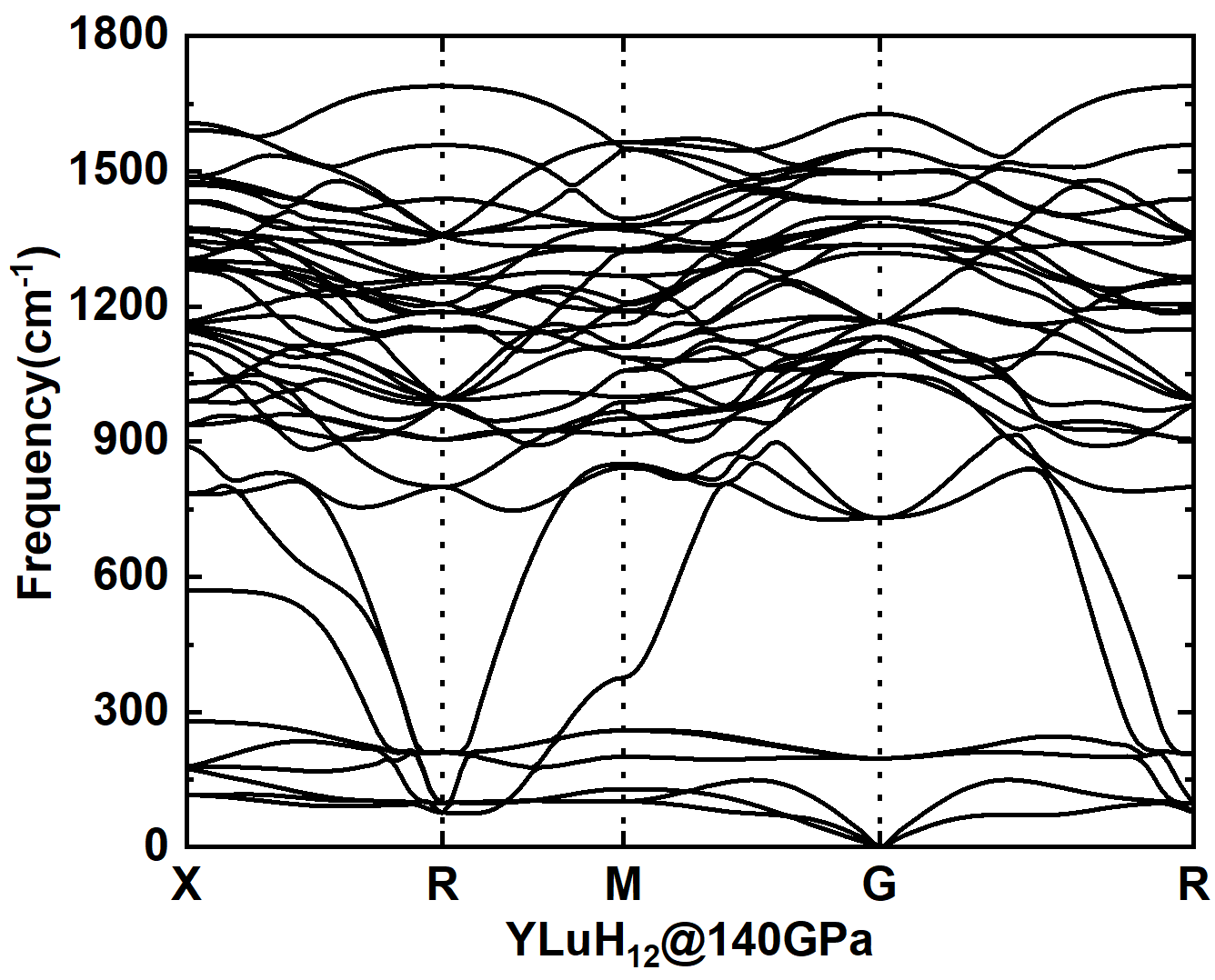


Fig. S9 The phonon band structure of YLu_2_H_18_, Y_2_LuH_18_, YLu_3_H_24_, Y_3_LuH_24_ and YLuH_12_ under their minimum dynamically stable pressures, respectively.


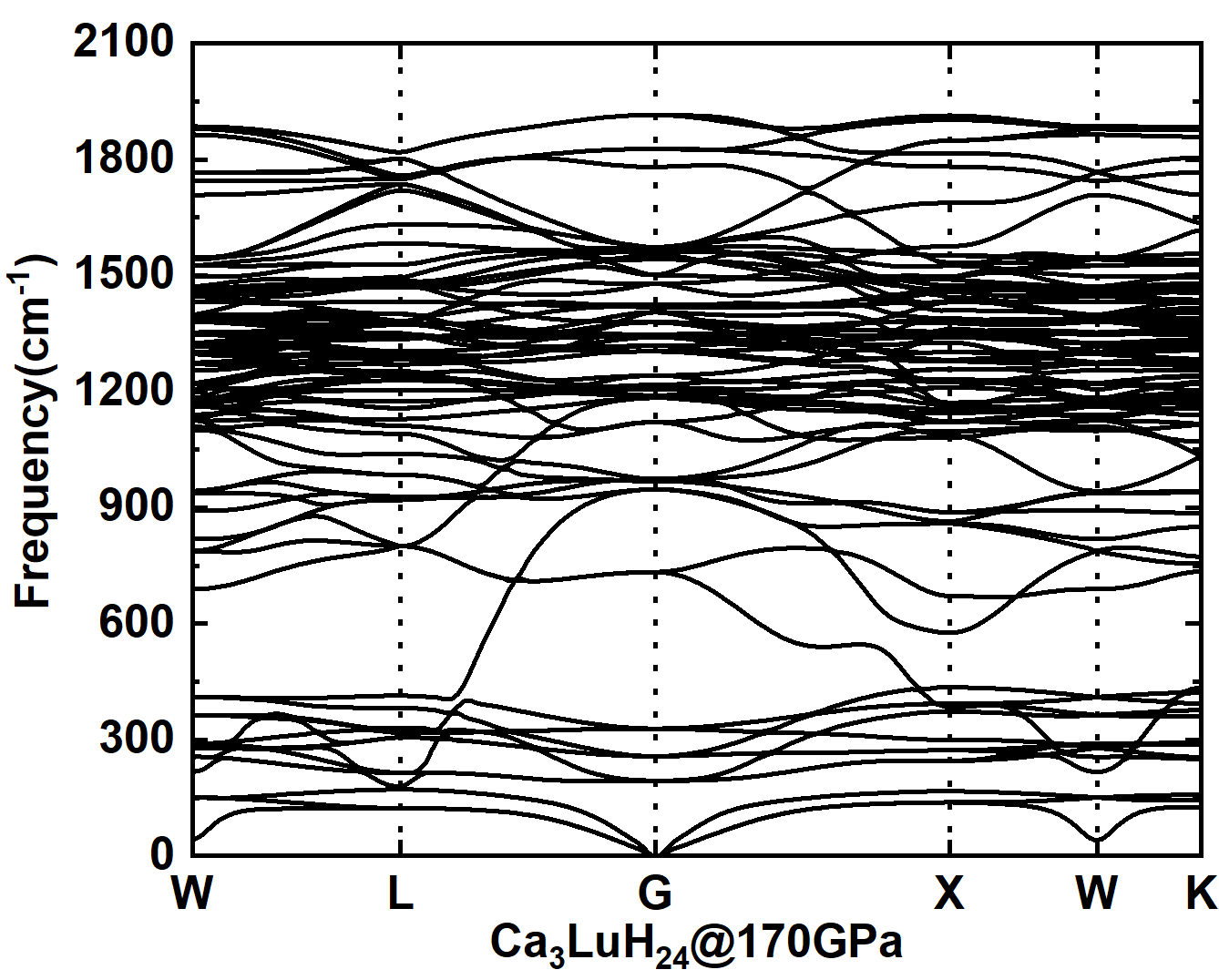

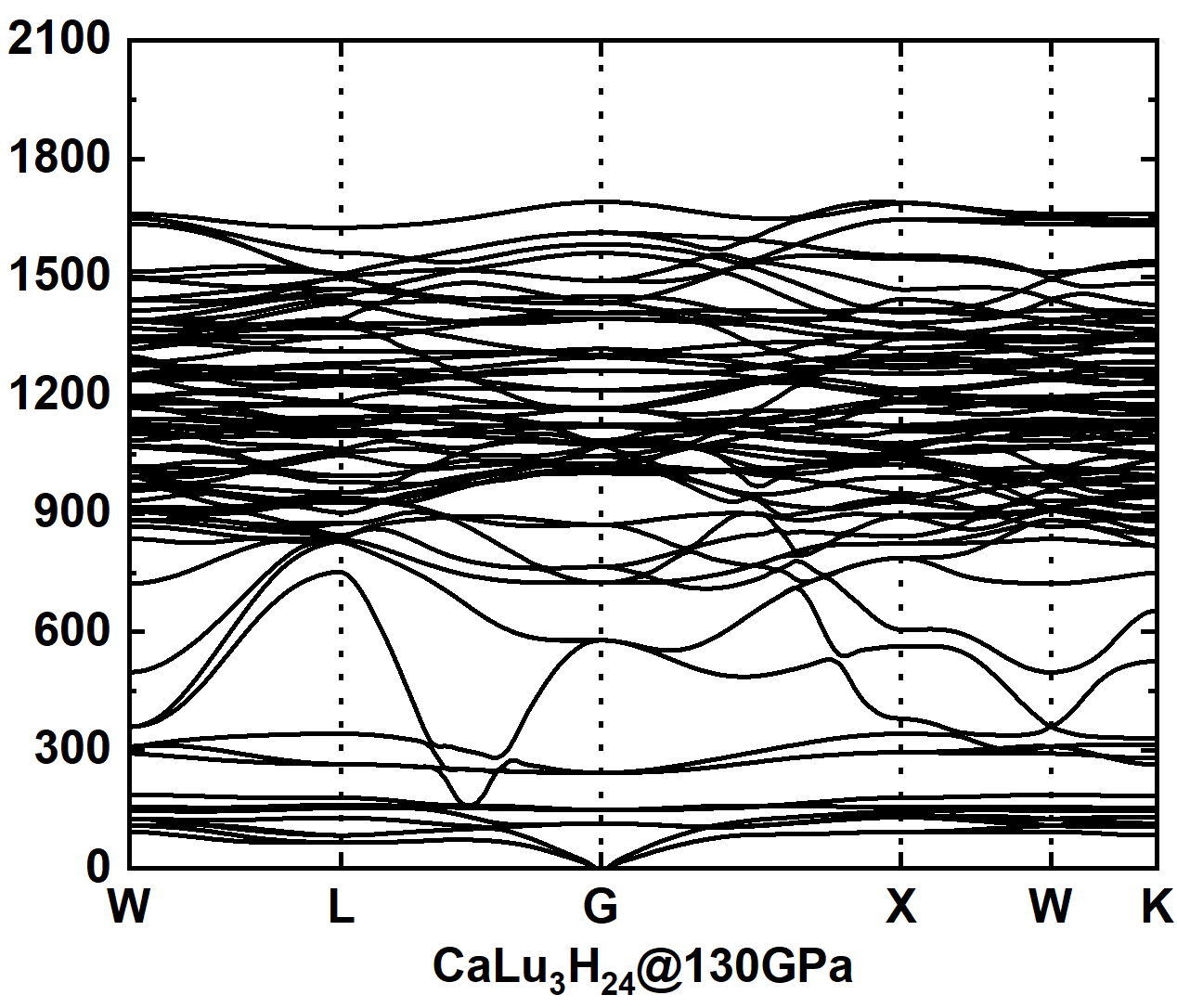


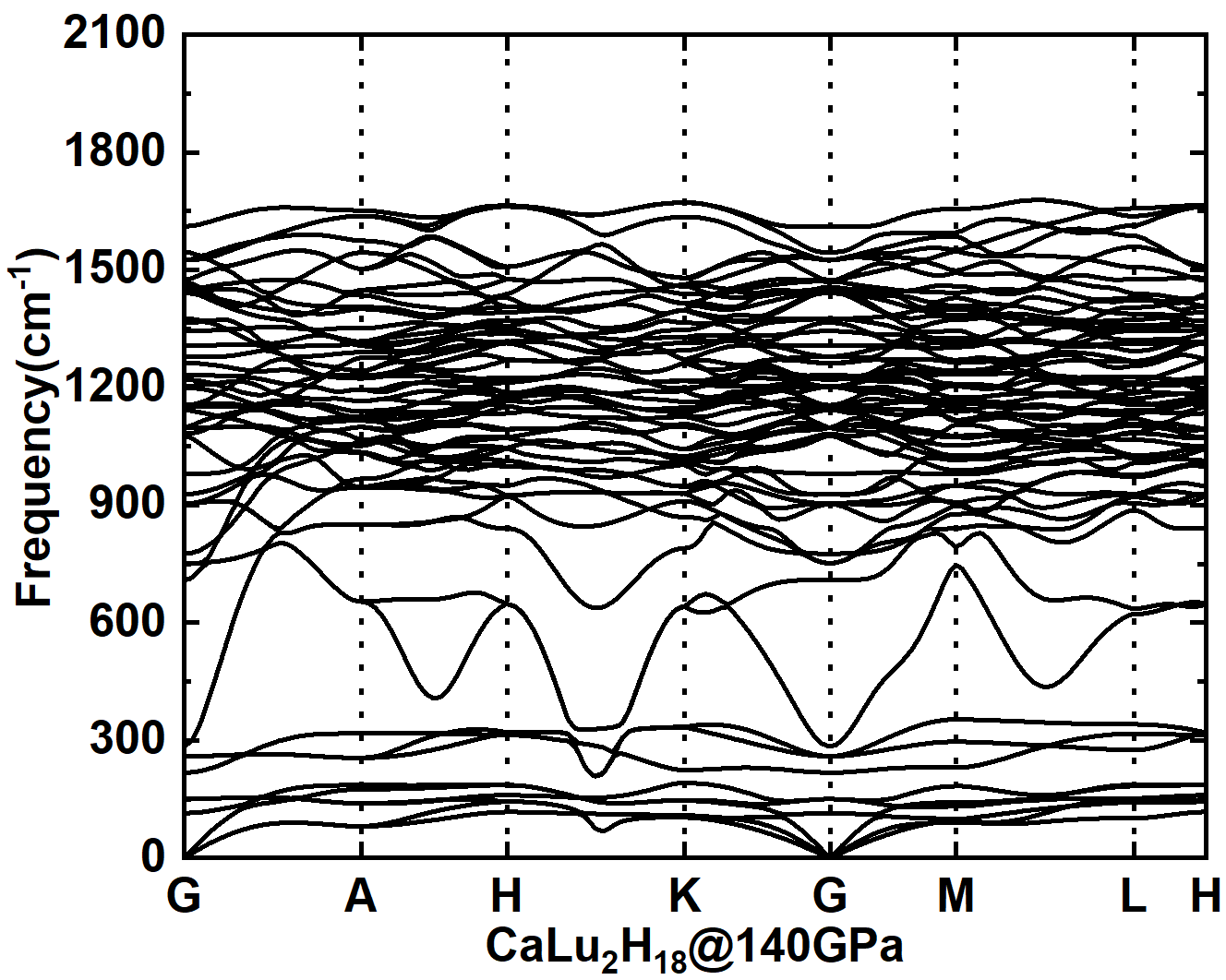

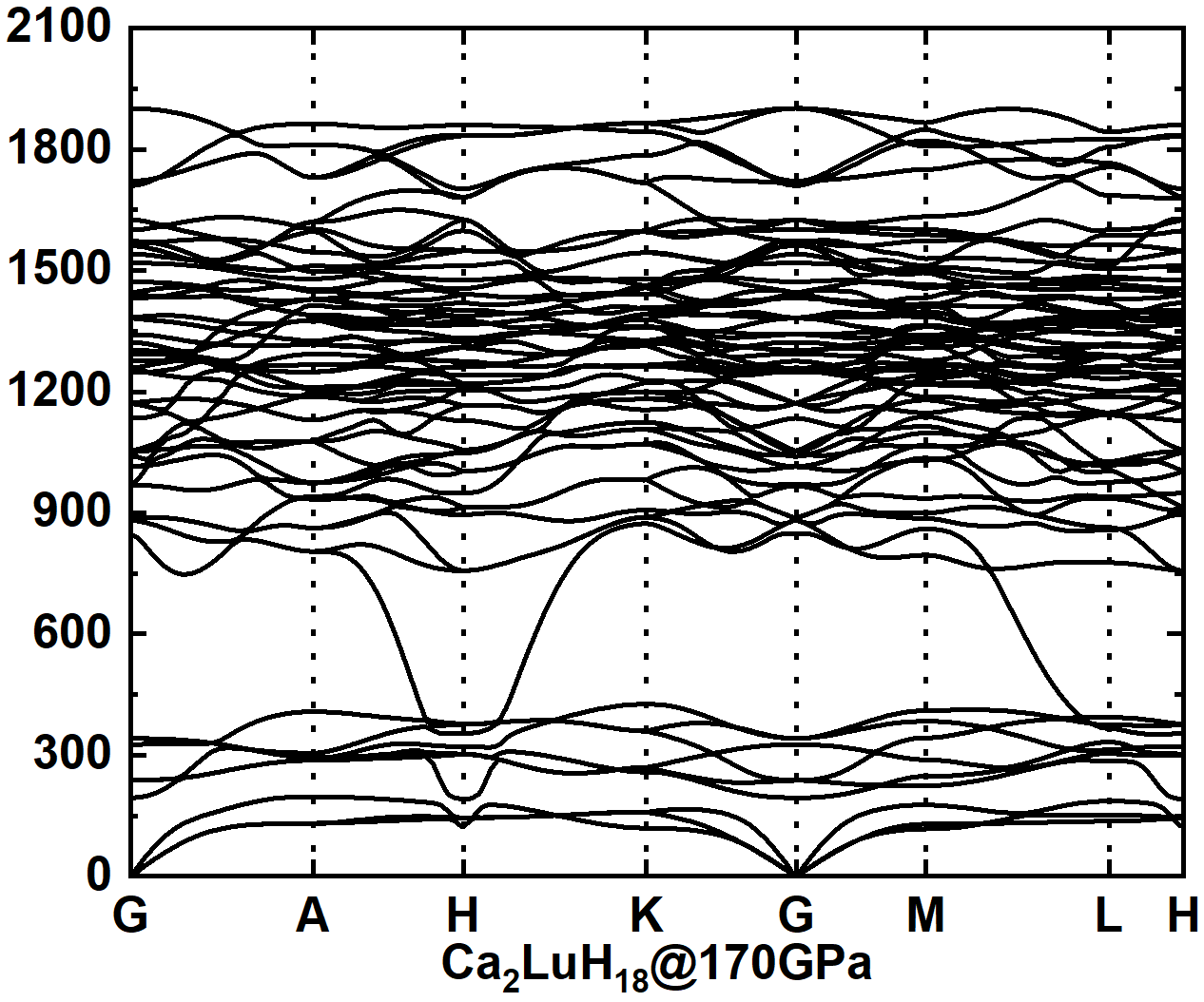


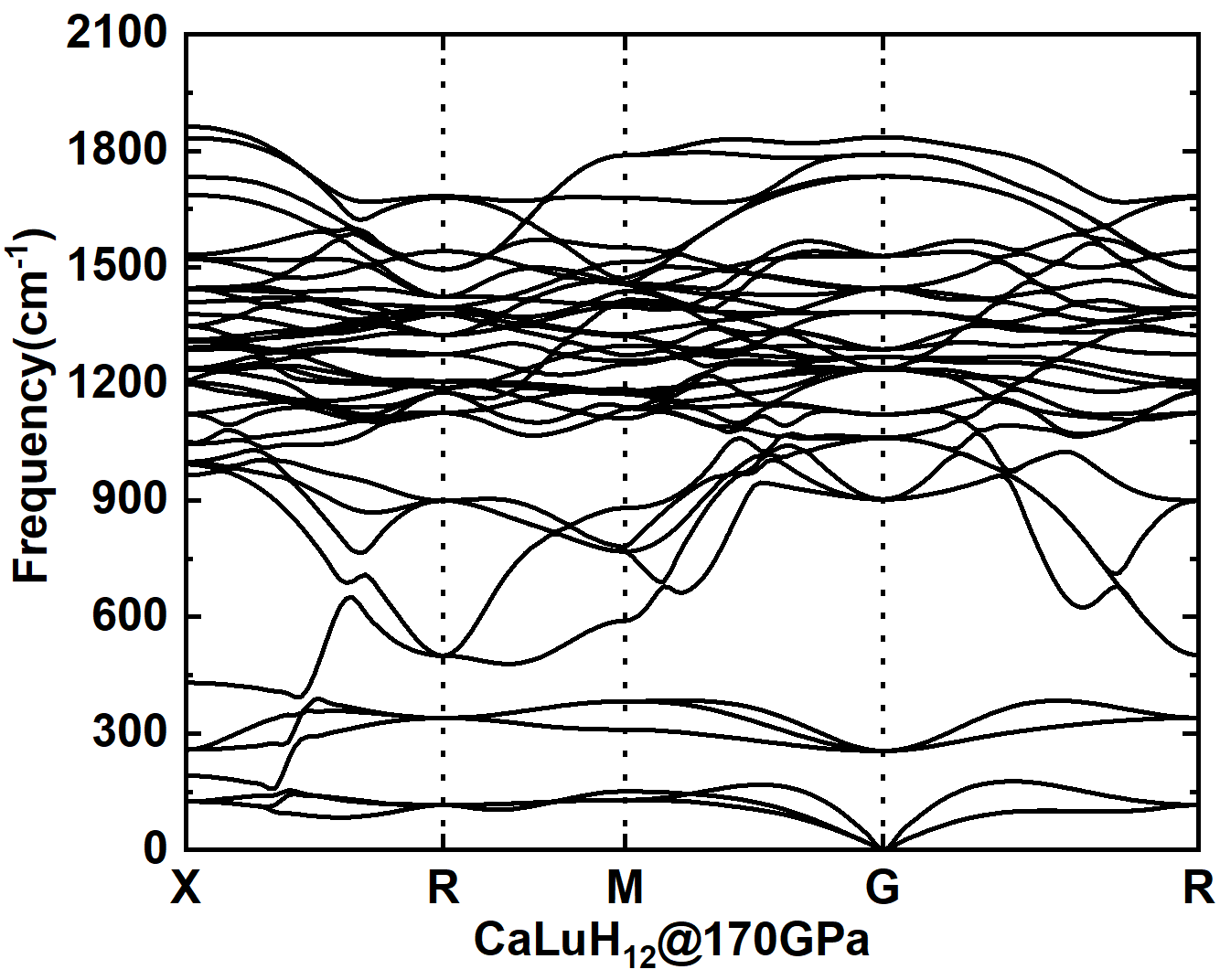


Fig. S10 The phonon band structure of Ca_3_LuH_24_, CaLu_3_H_24_, CaLu_2_H_18_, Ca_2_LuH_18_, Ca_3_LuH_24_ and CaLuH_12_ under their minimum dynamically stable pressures, respectively.


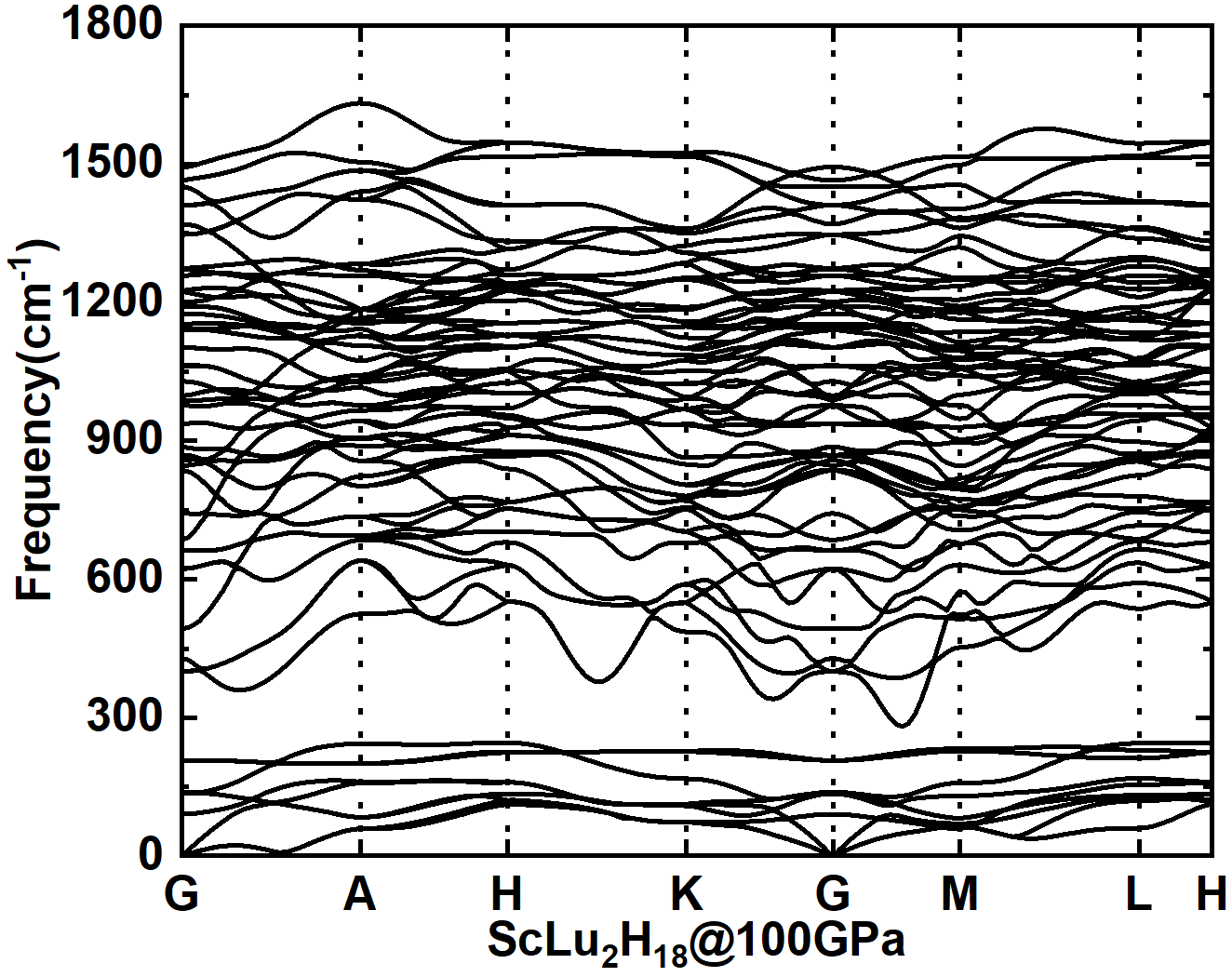

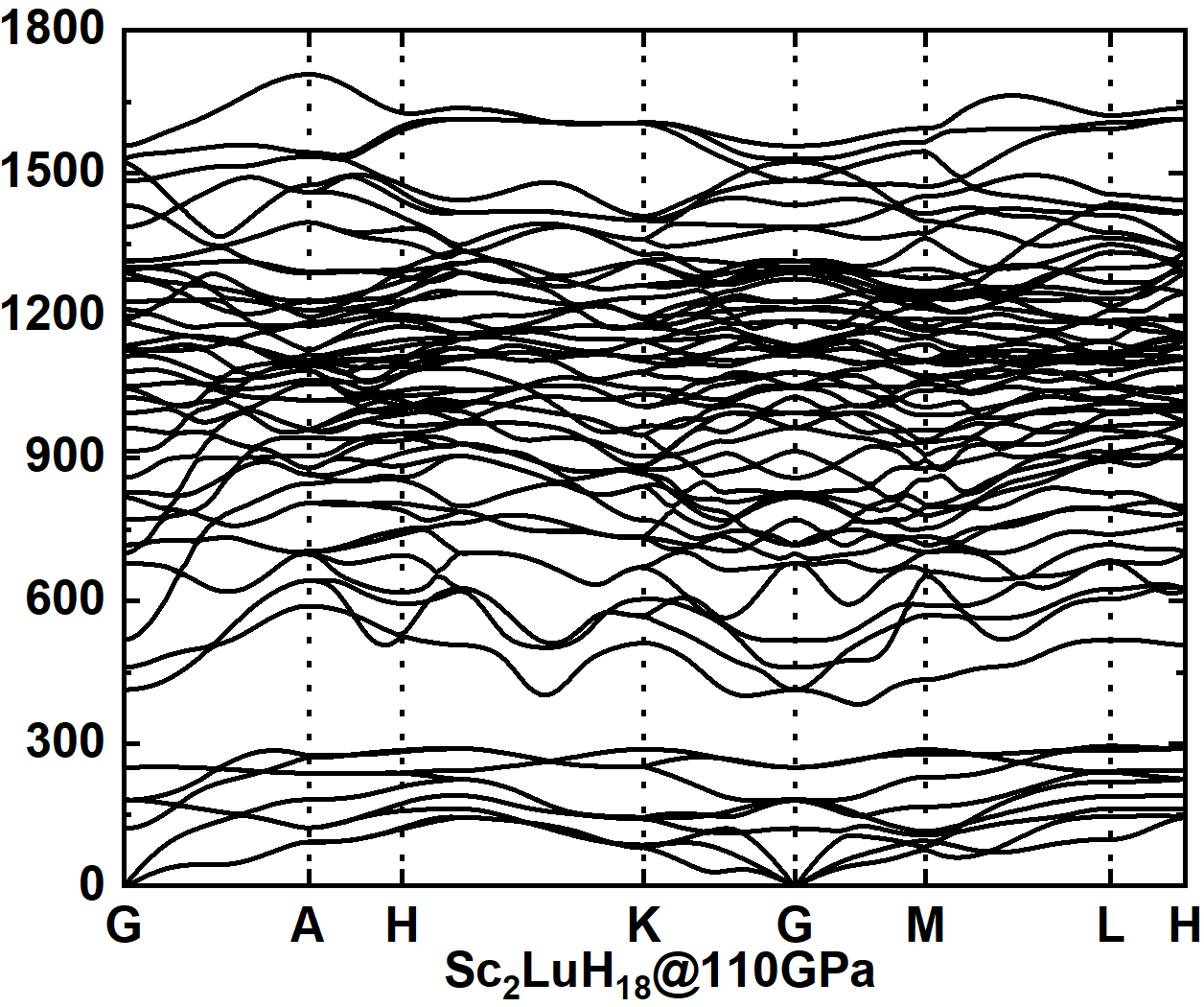


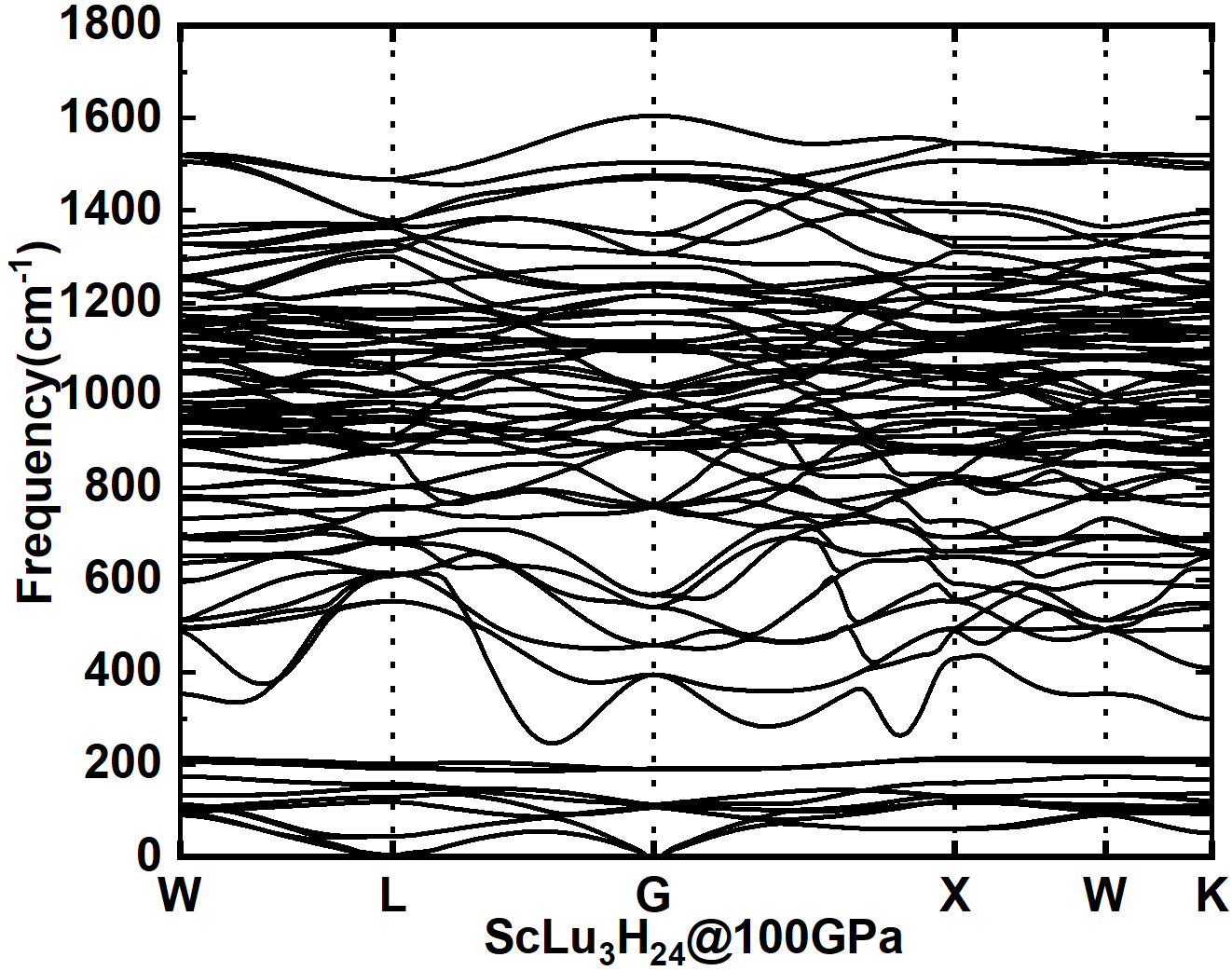

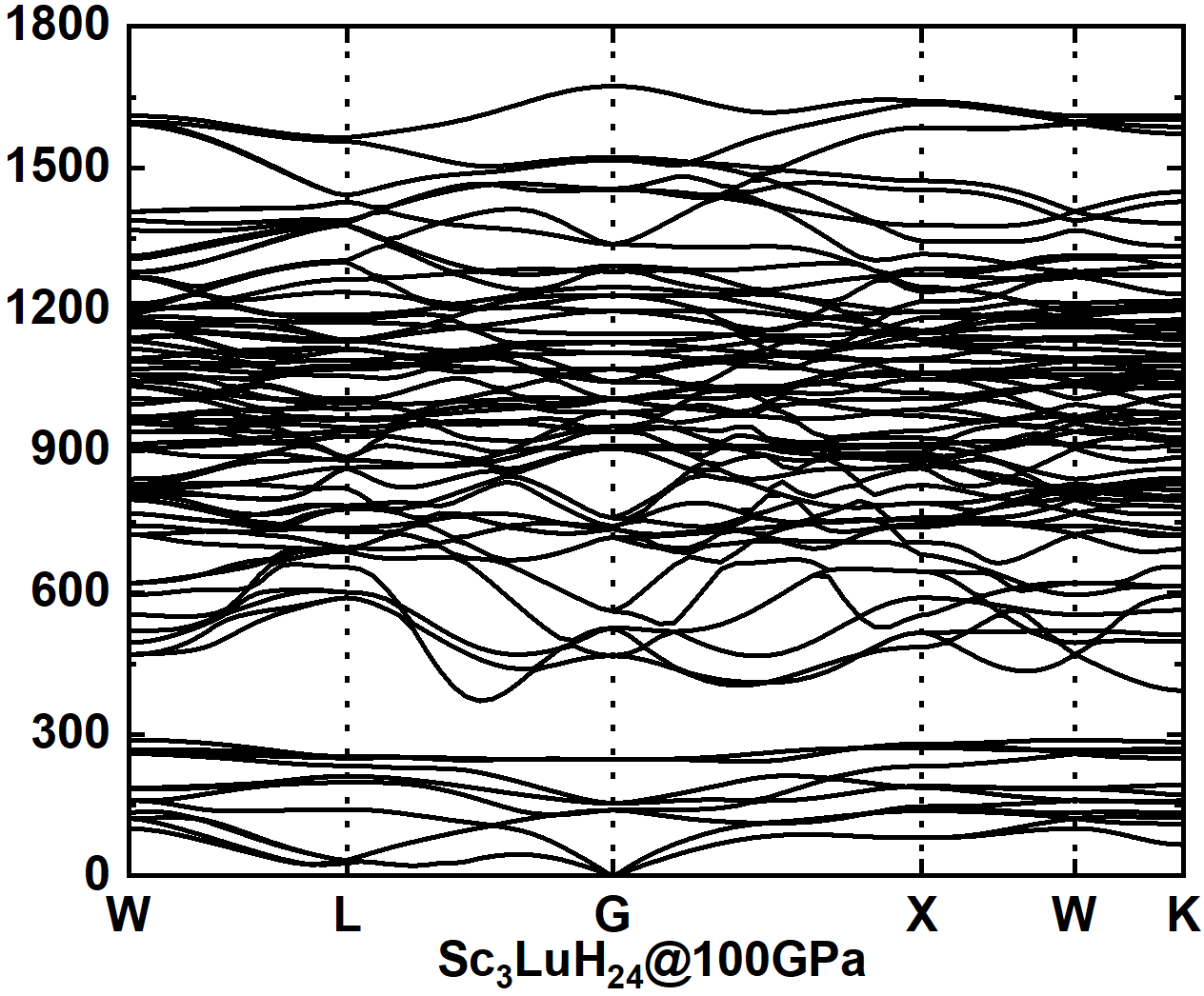


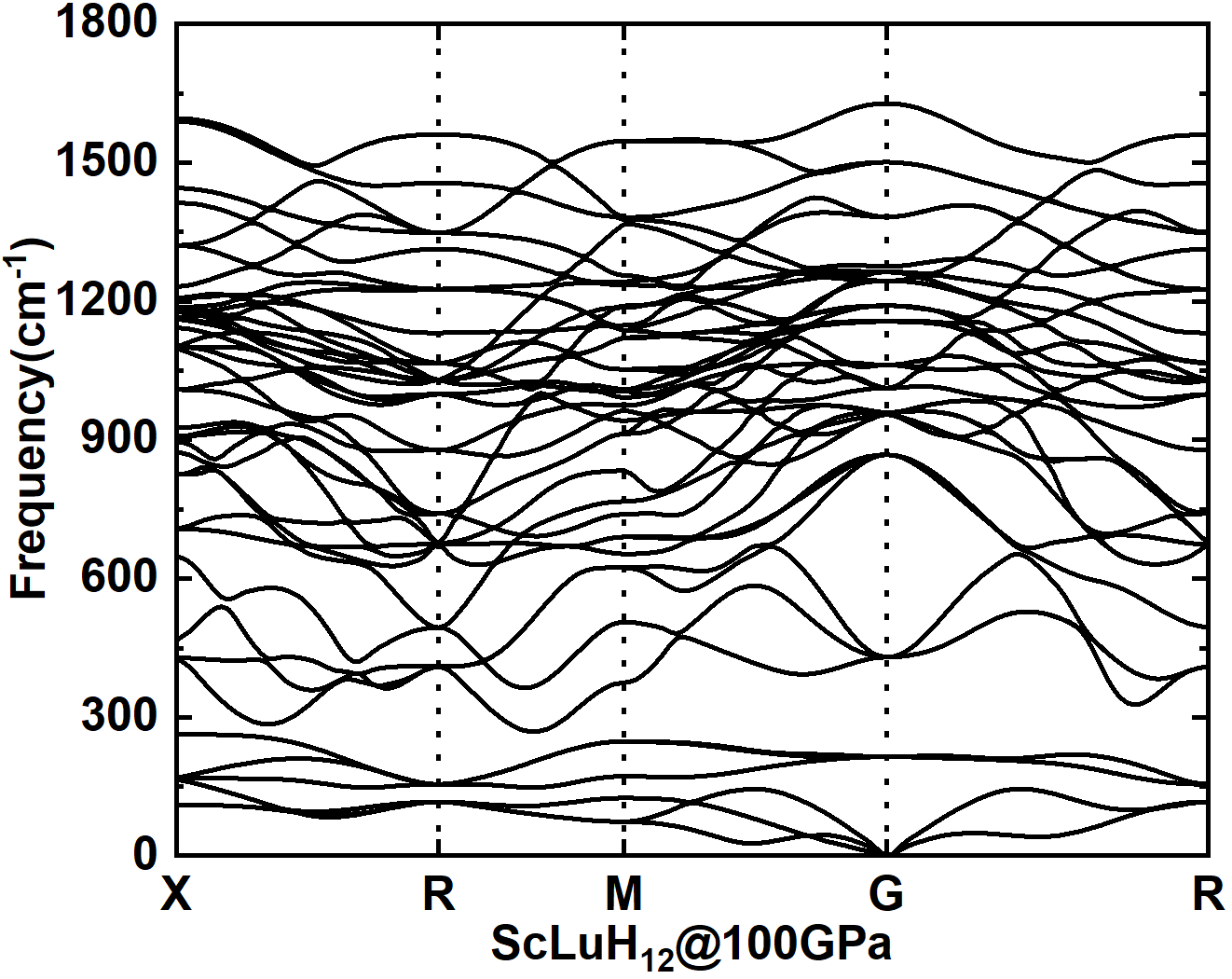

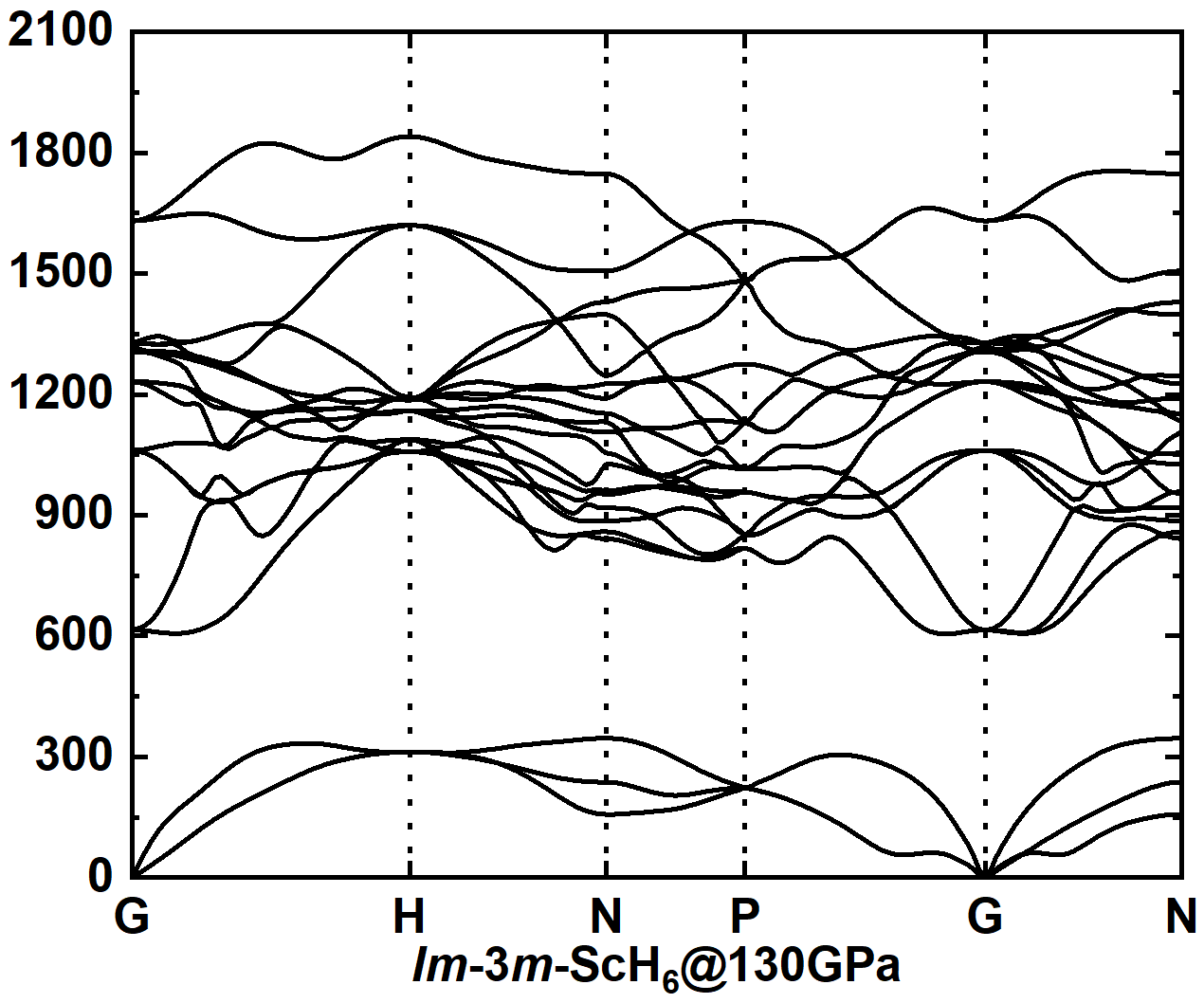


Fig. S11 The phonon band structure of ScLu_2_H_18_, Sc_2_LuH_18_, ScLu_3_H_24_, Sc_3_LuH_24_ ScLuH_12_, and ScH_6_ under their minimum dynamically stable pressures, respectively.


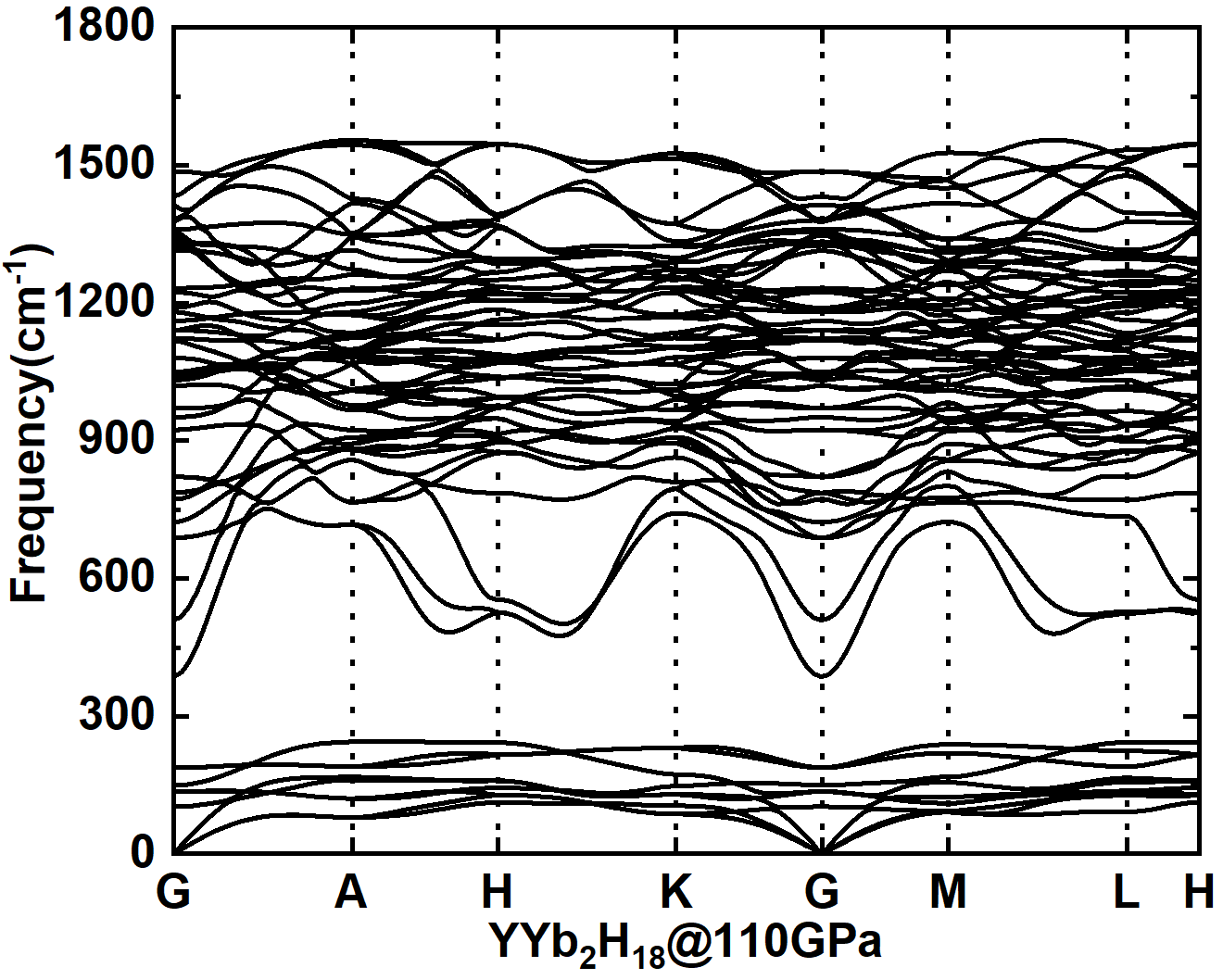

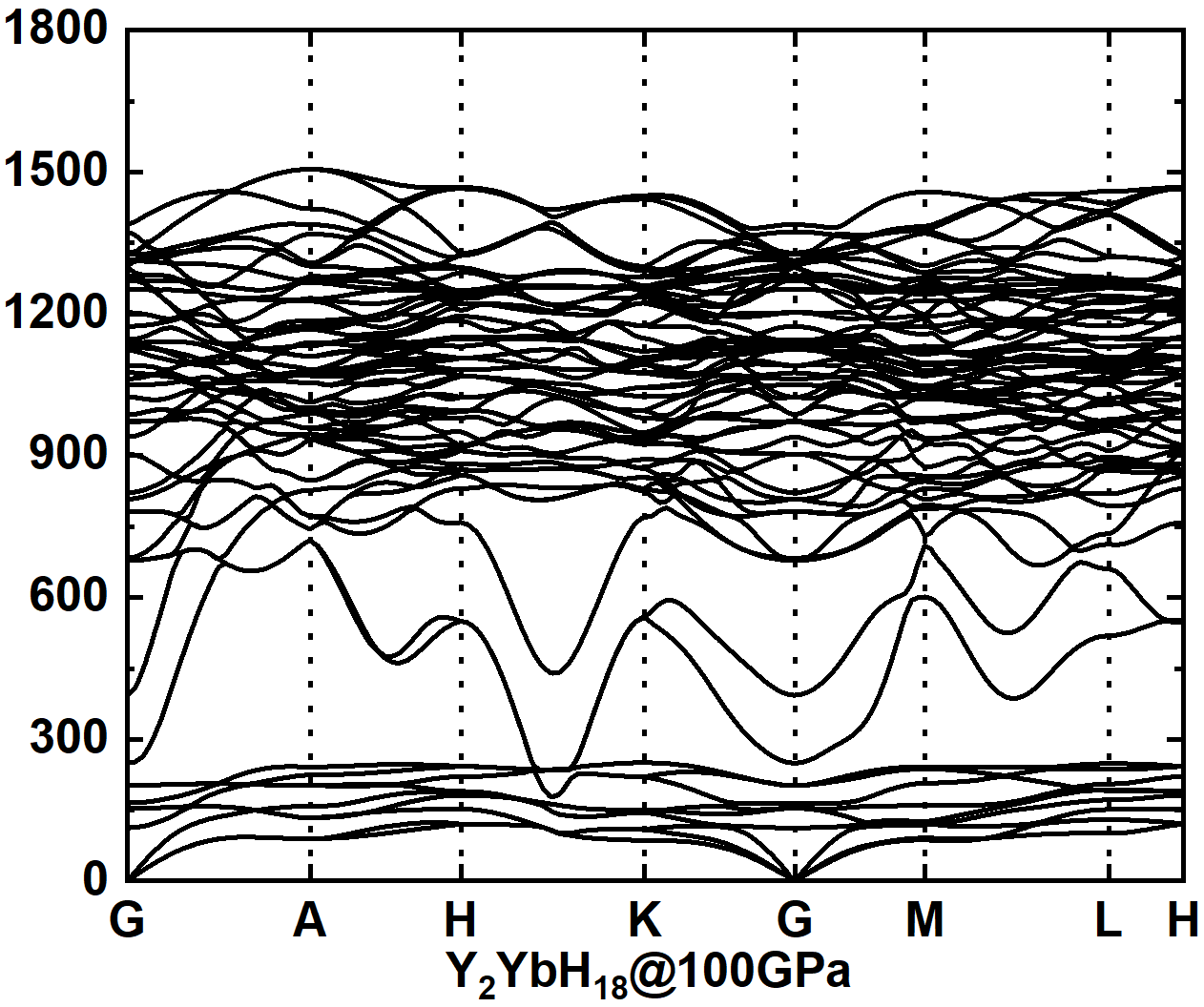


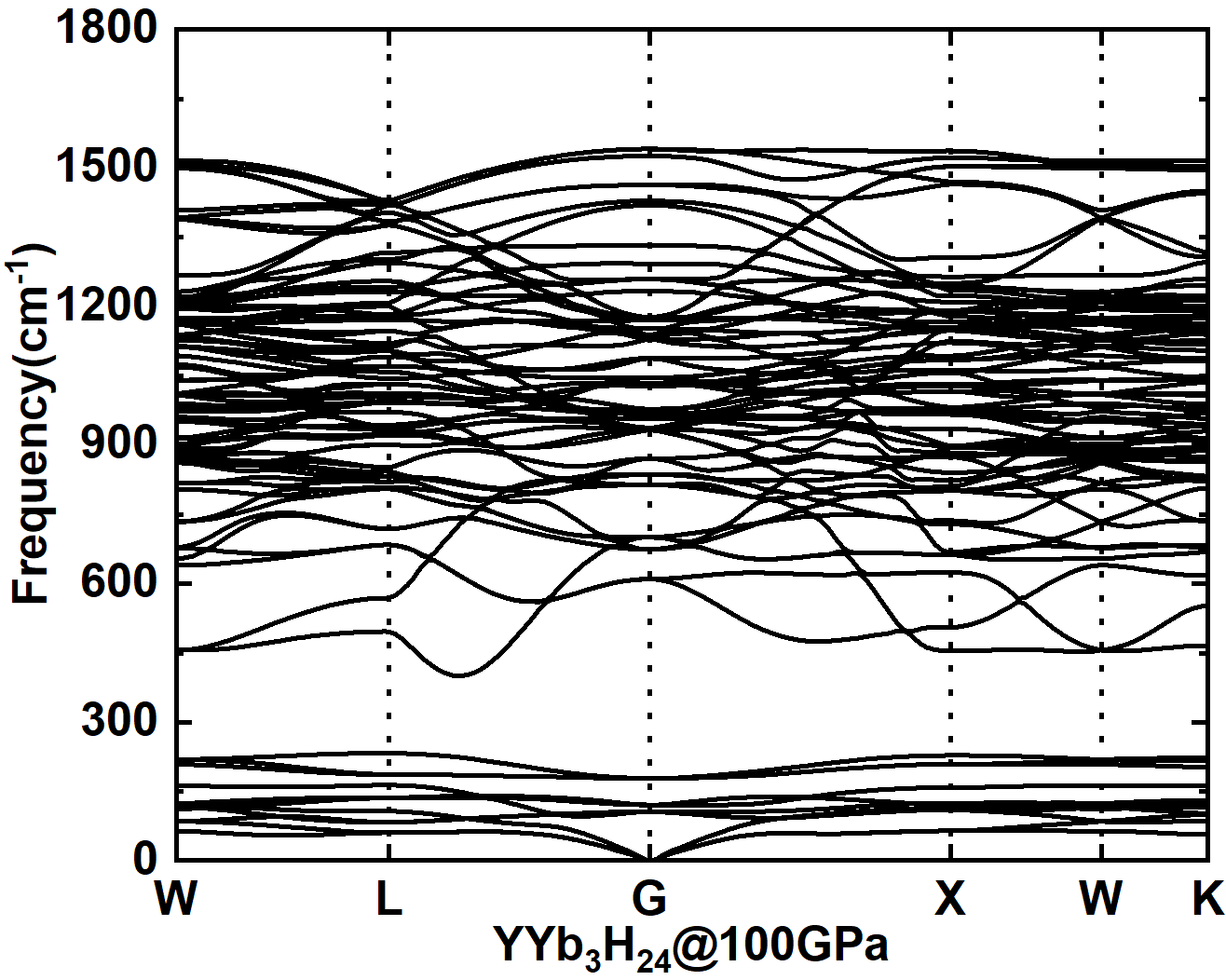

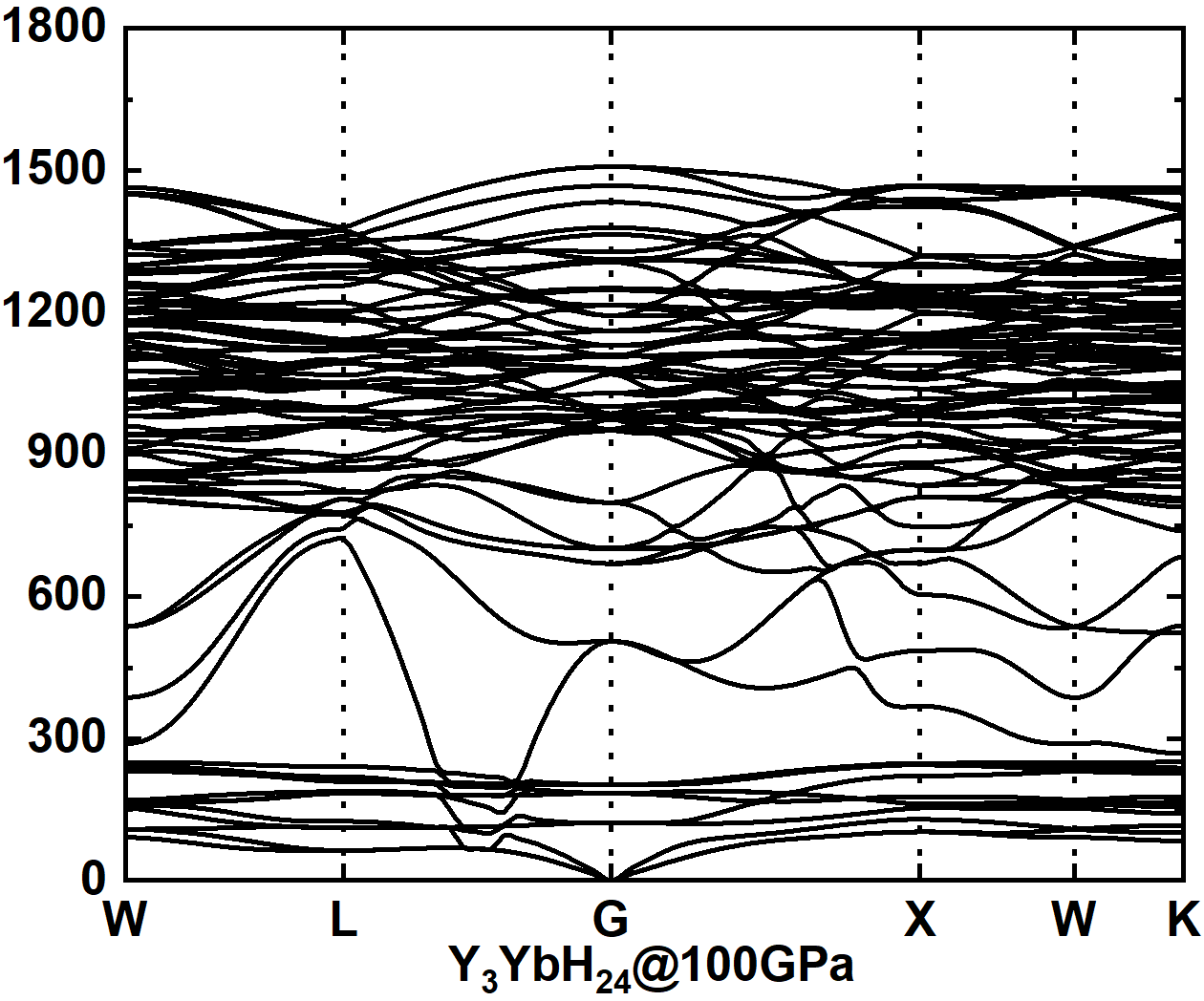


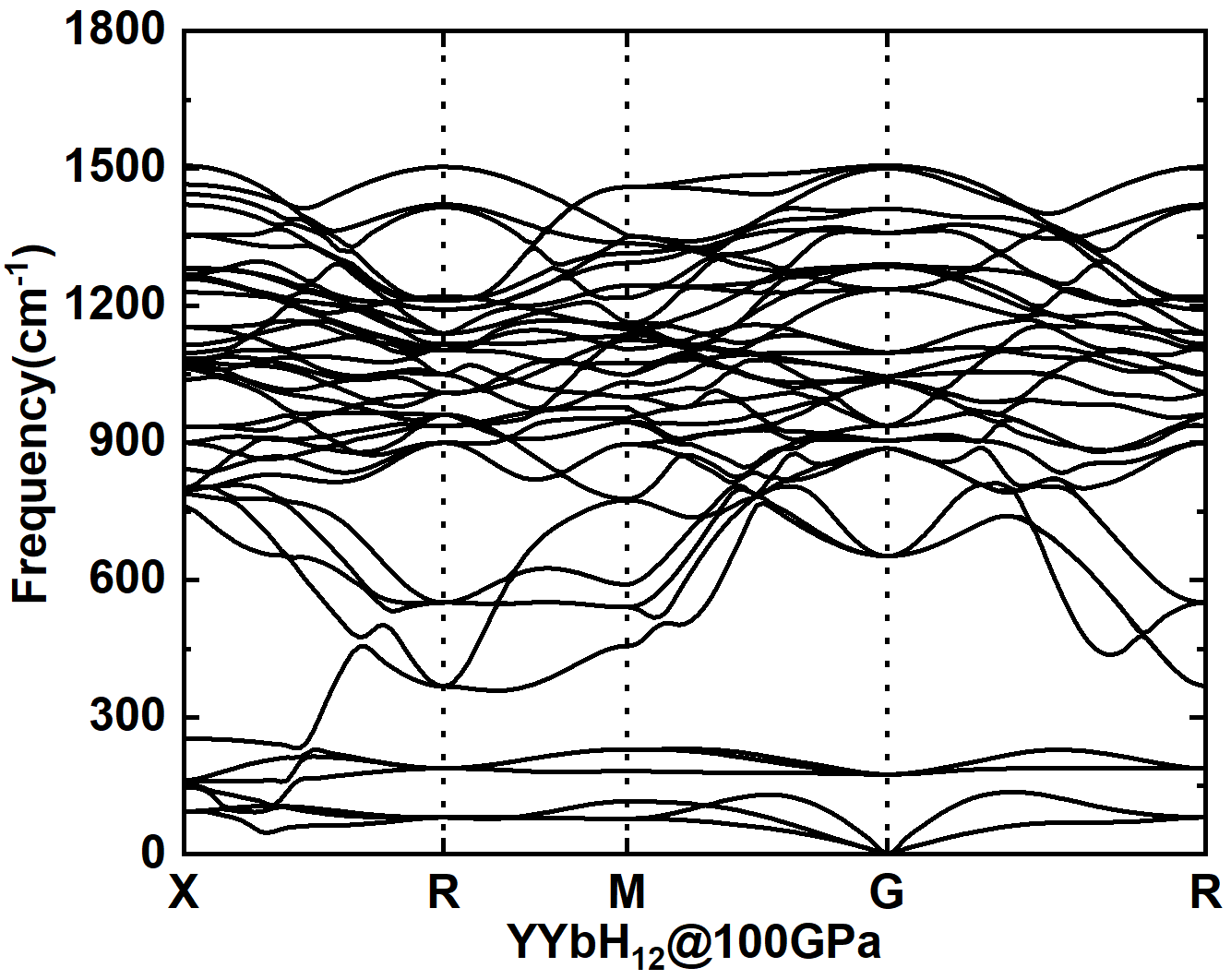


Fig. S12 The phonon band structure of YYbH_12_, YYb_2_H_18_, Y_2_YbH_18_, YYb_3_H_24_ and Y_3_YbH_24_ under their minimum dynamically stable pressures, respectively.


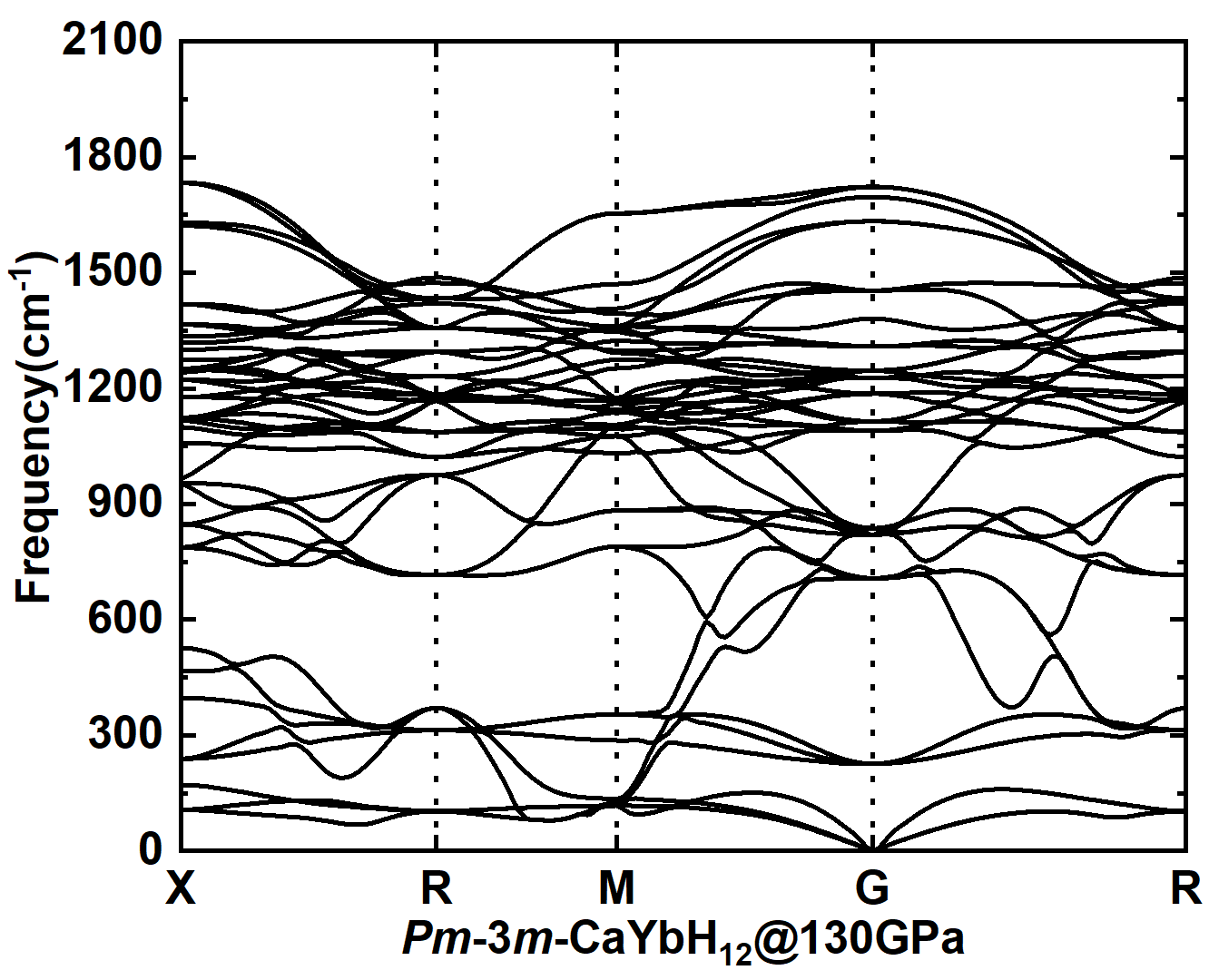

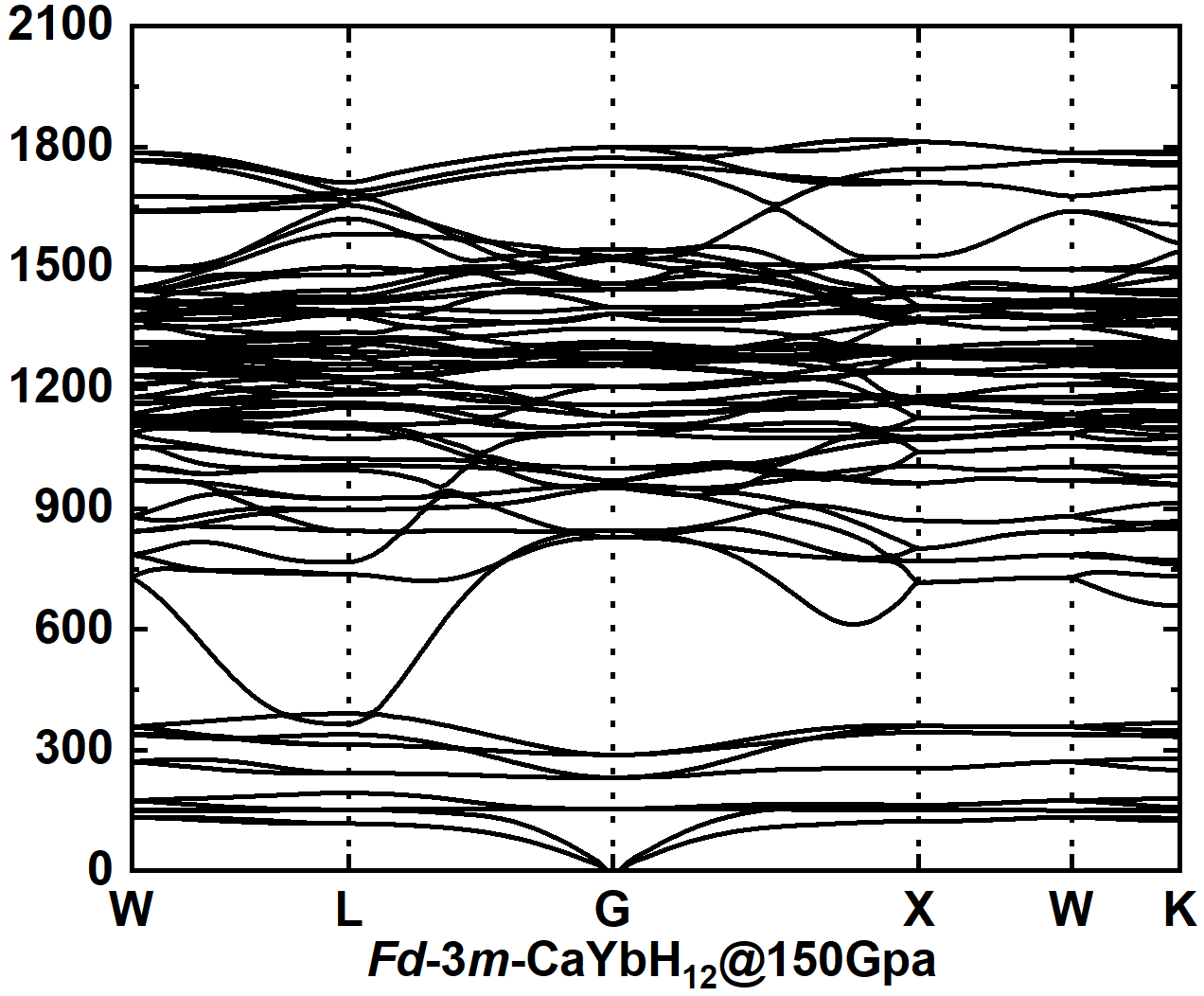


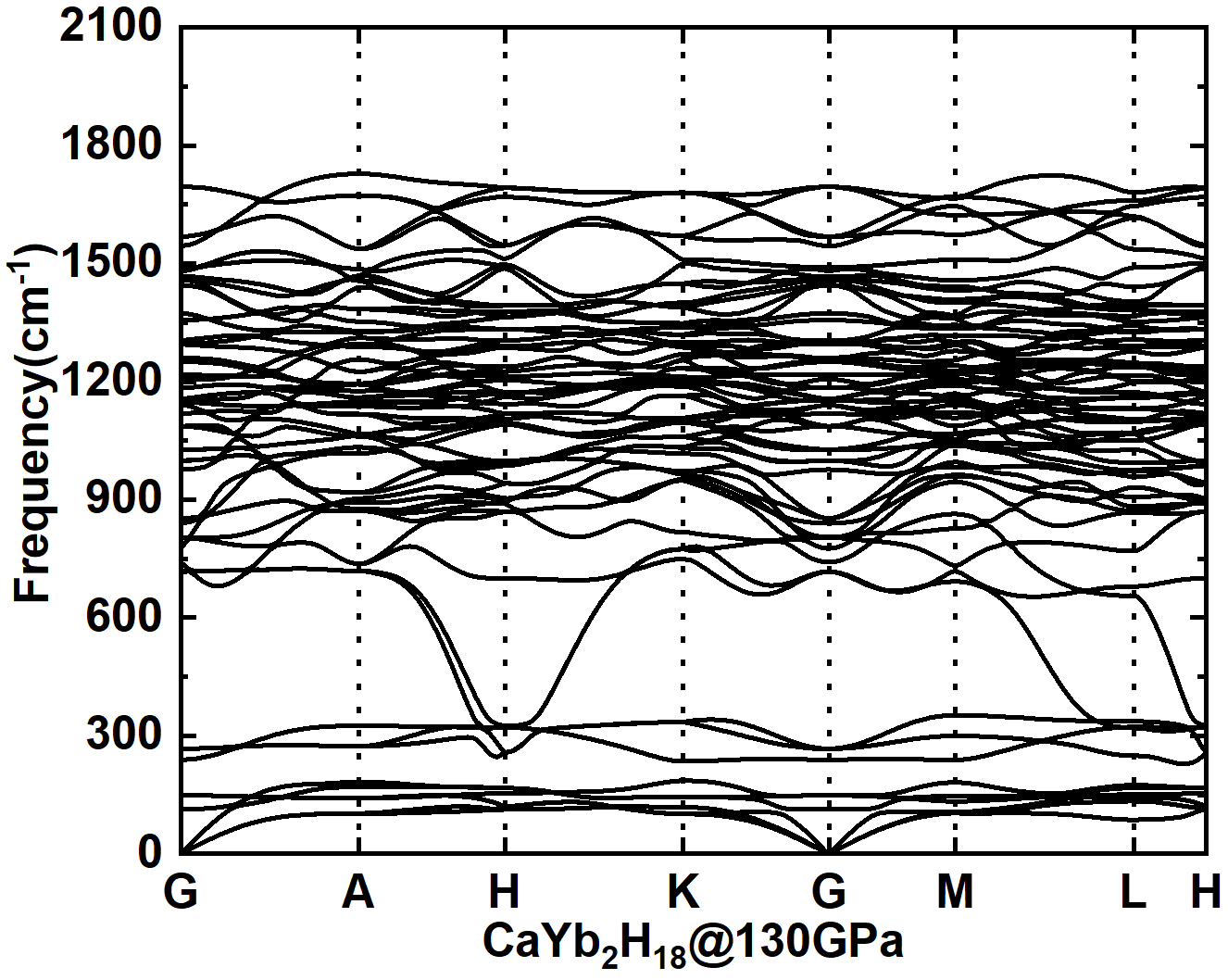

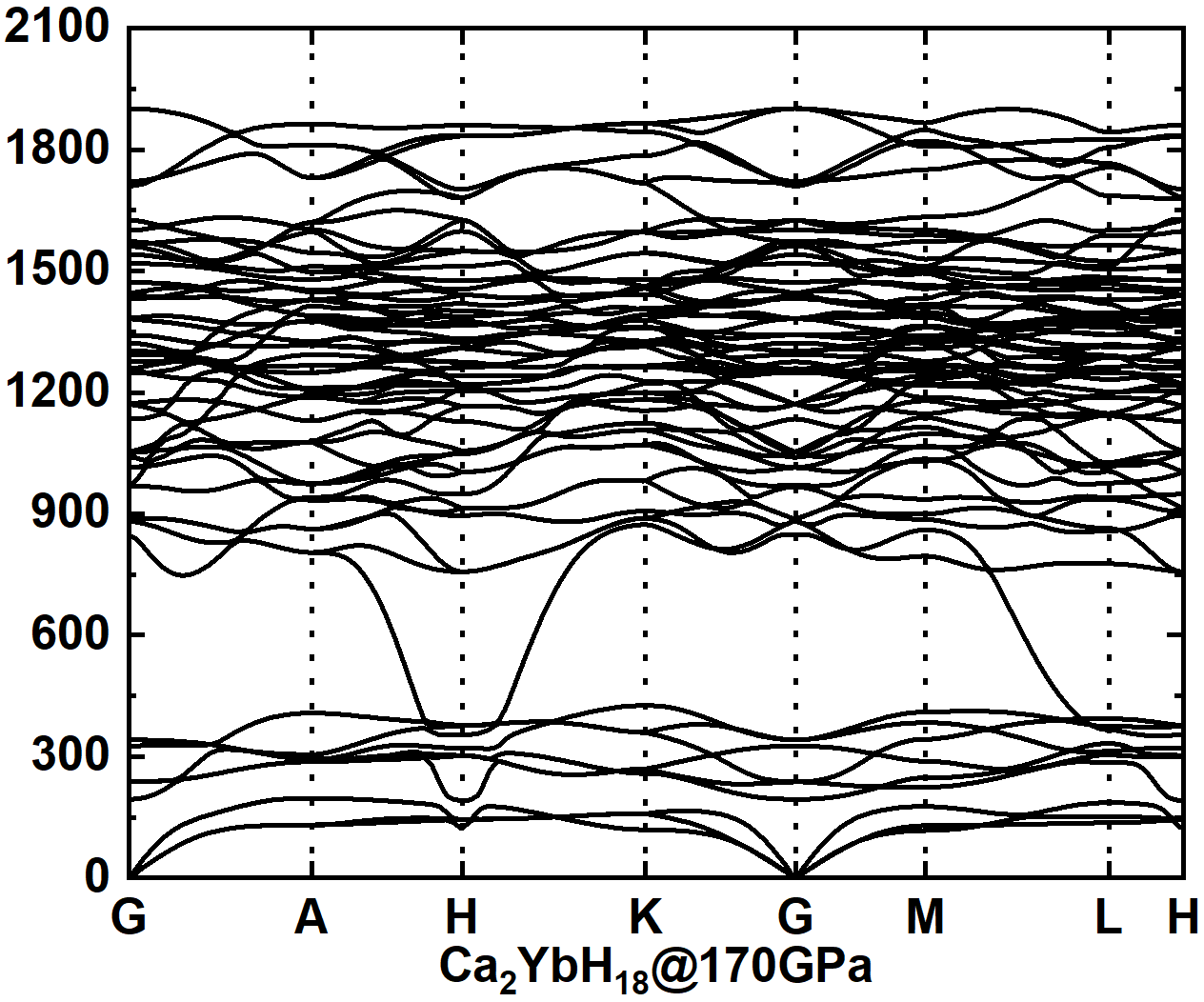


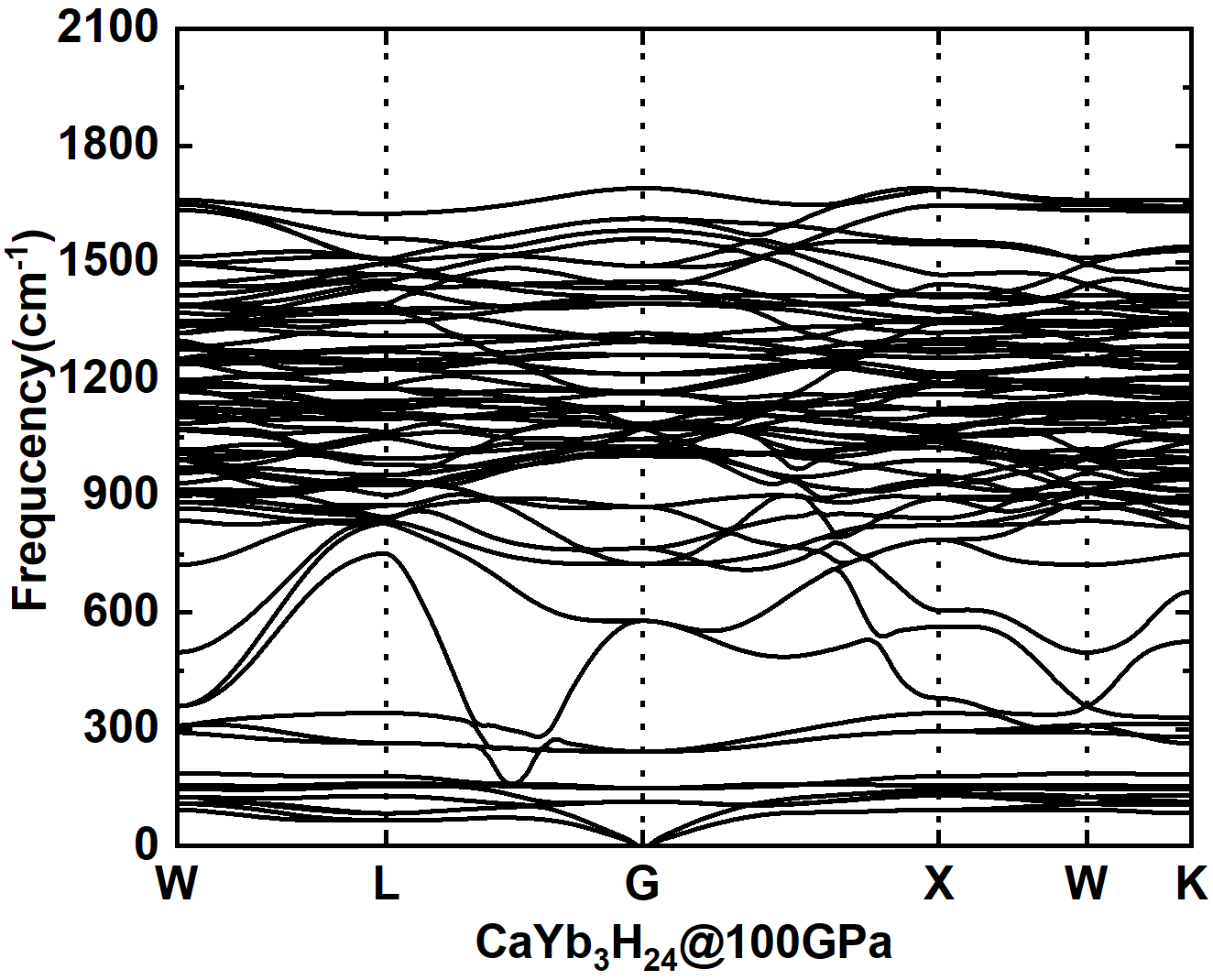

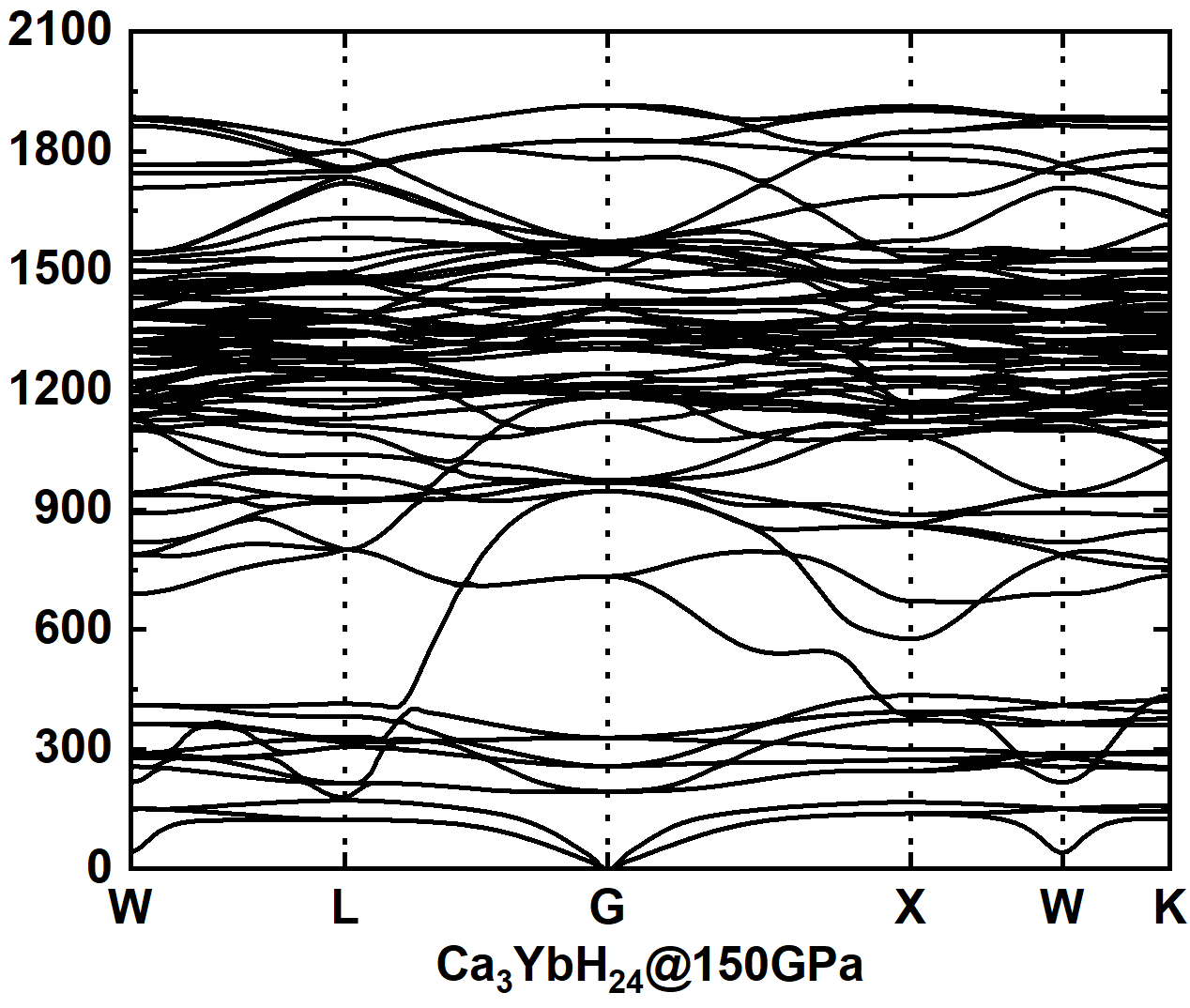


Fig. S13 The phonon band structure of *Pm*-3*m*-CaYbH_12_, *Fd*-3*m*-CaYbH_12_, CaYb_2_H_18_, Ca_2_YbH_18_, CaYb_3_H_24_ and Ca_3_YbH_24_ under their minimum dynamically stable pressures, respectively.


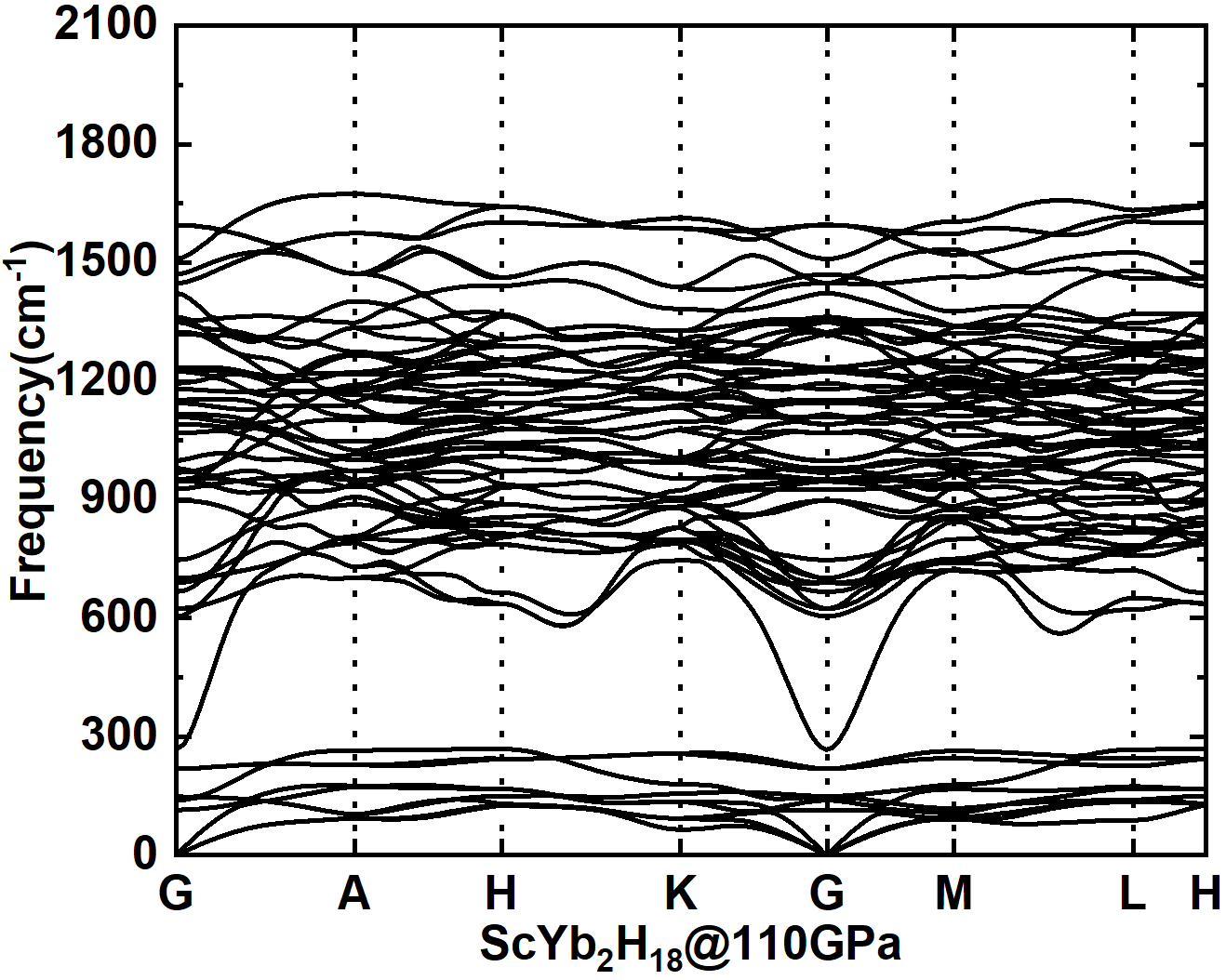

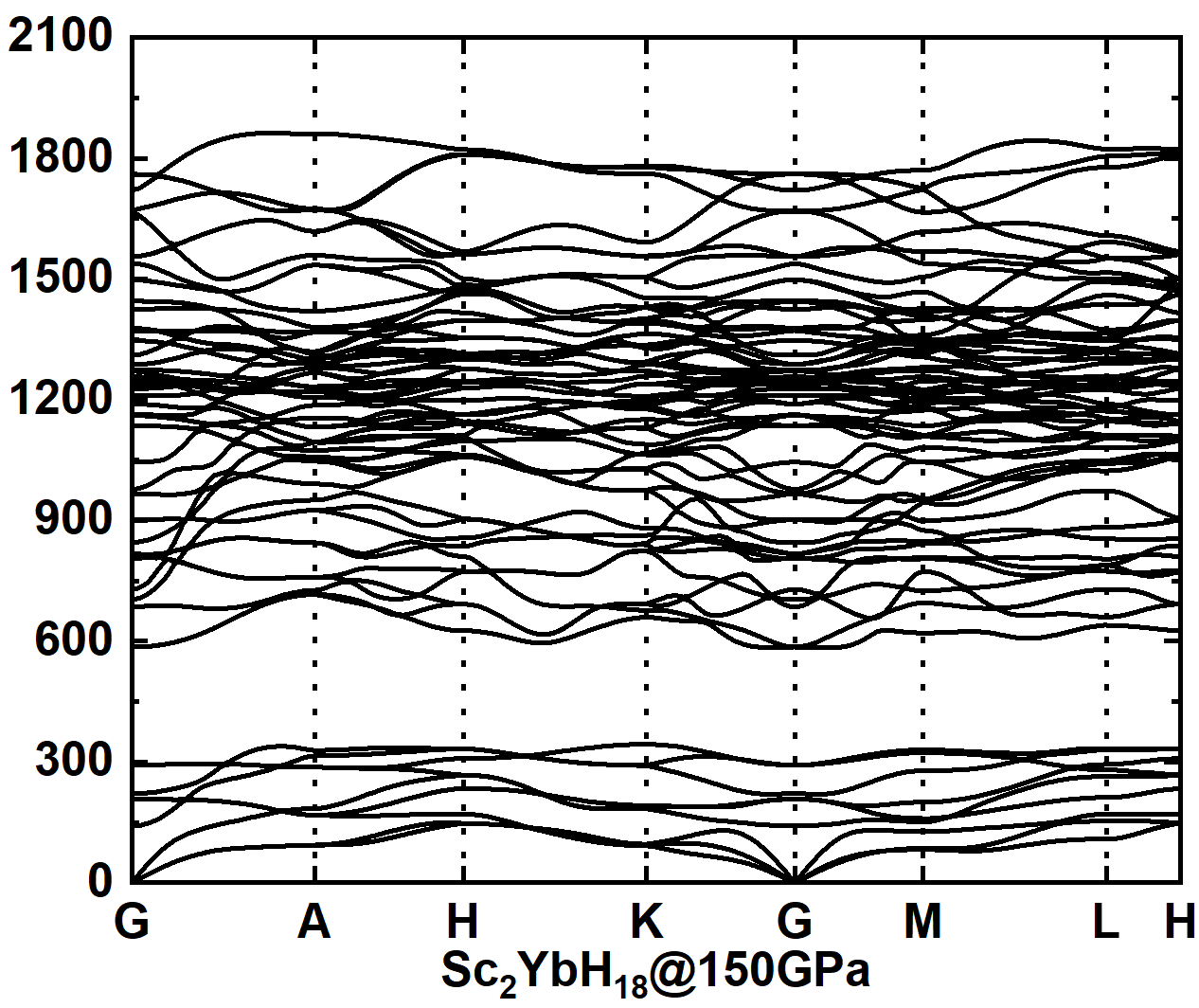


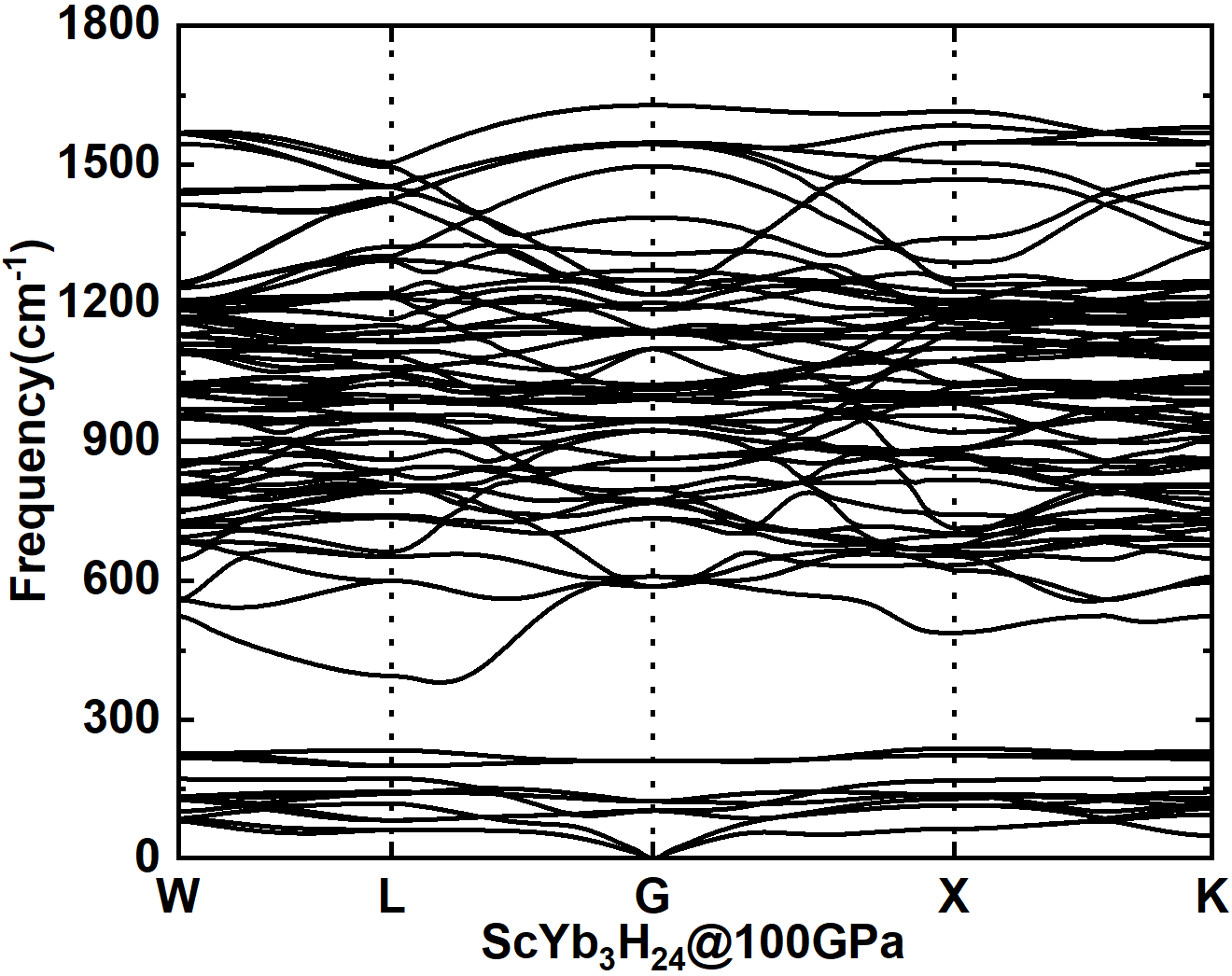

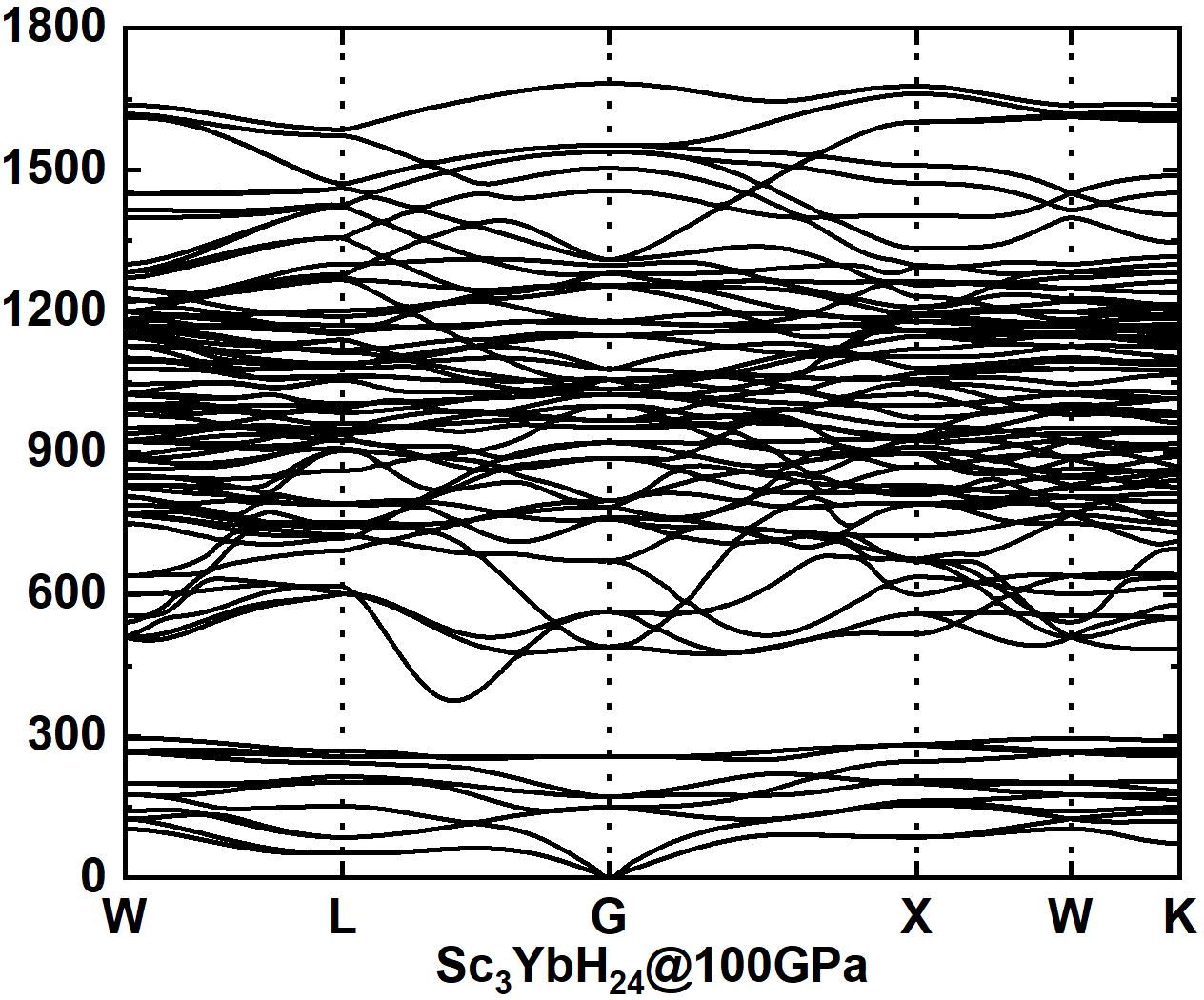


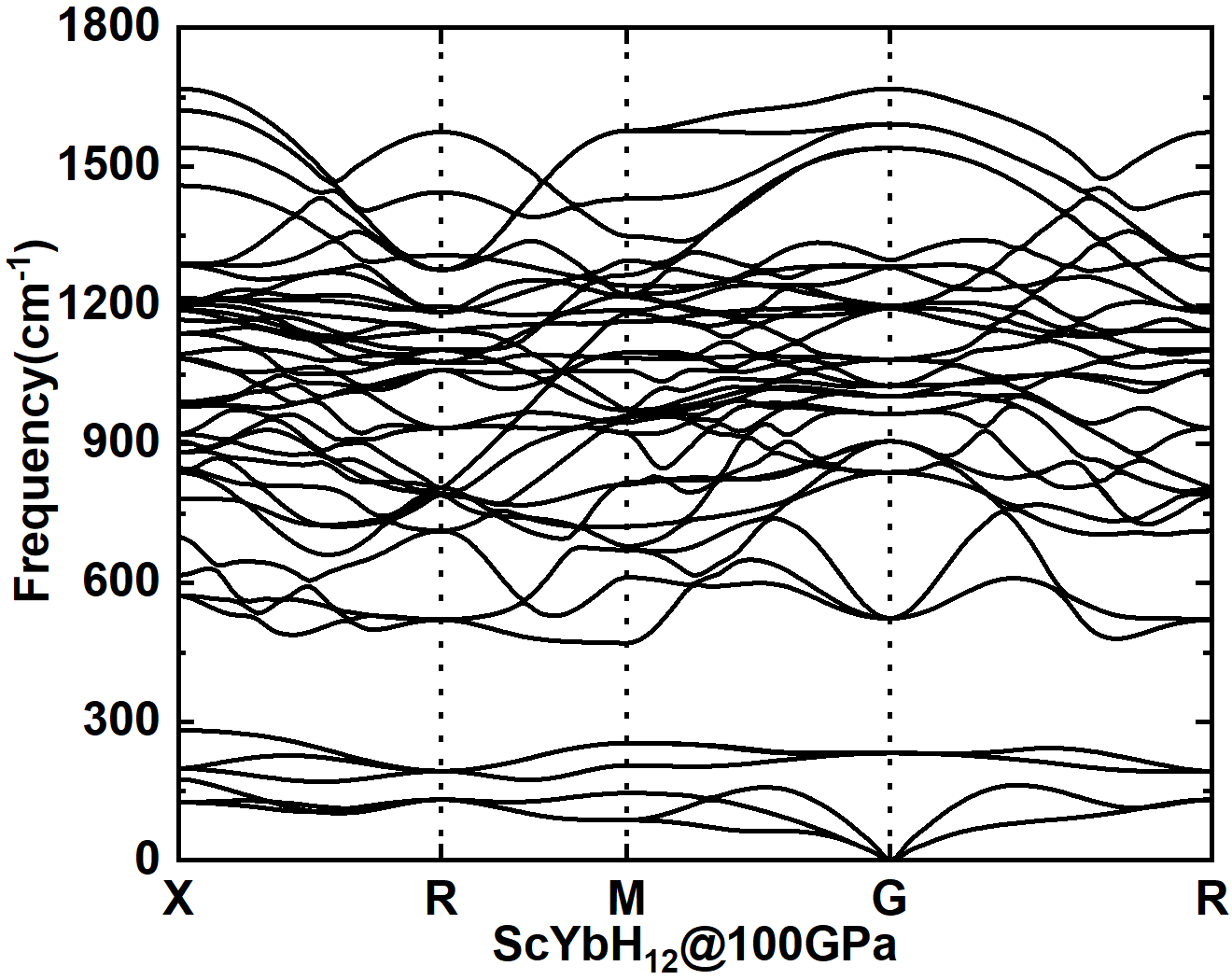


Fig. S14 The phonon band structure of ScYb_2_H_18_, Sc_2_YbH_18_, ScYb_3_H_24_, Sc_3_YbH_24_ and ScYbH_12_ under their minimum dynamically stable pressures, respectively.


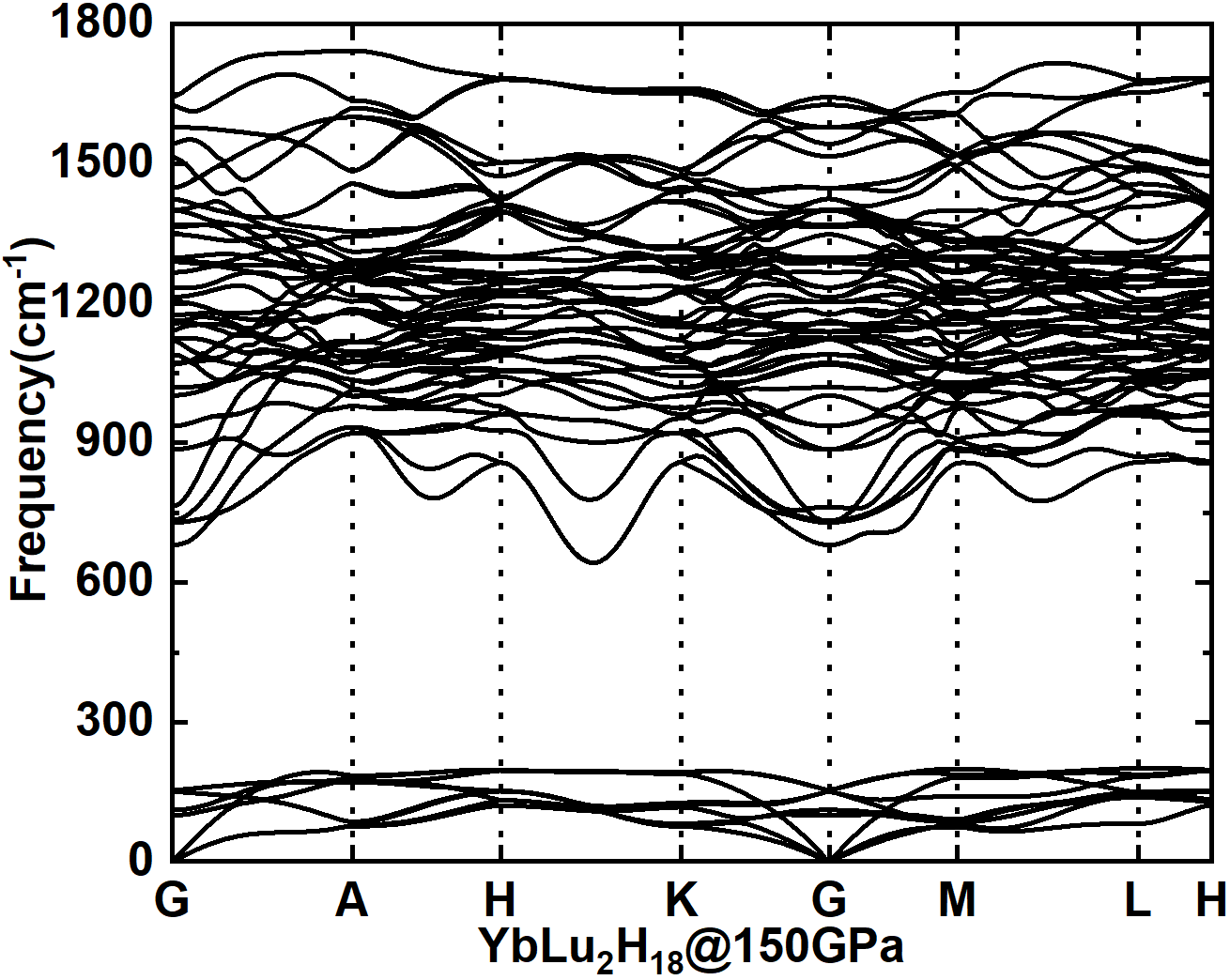

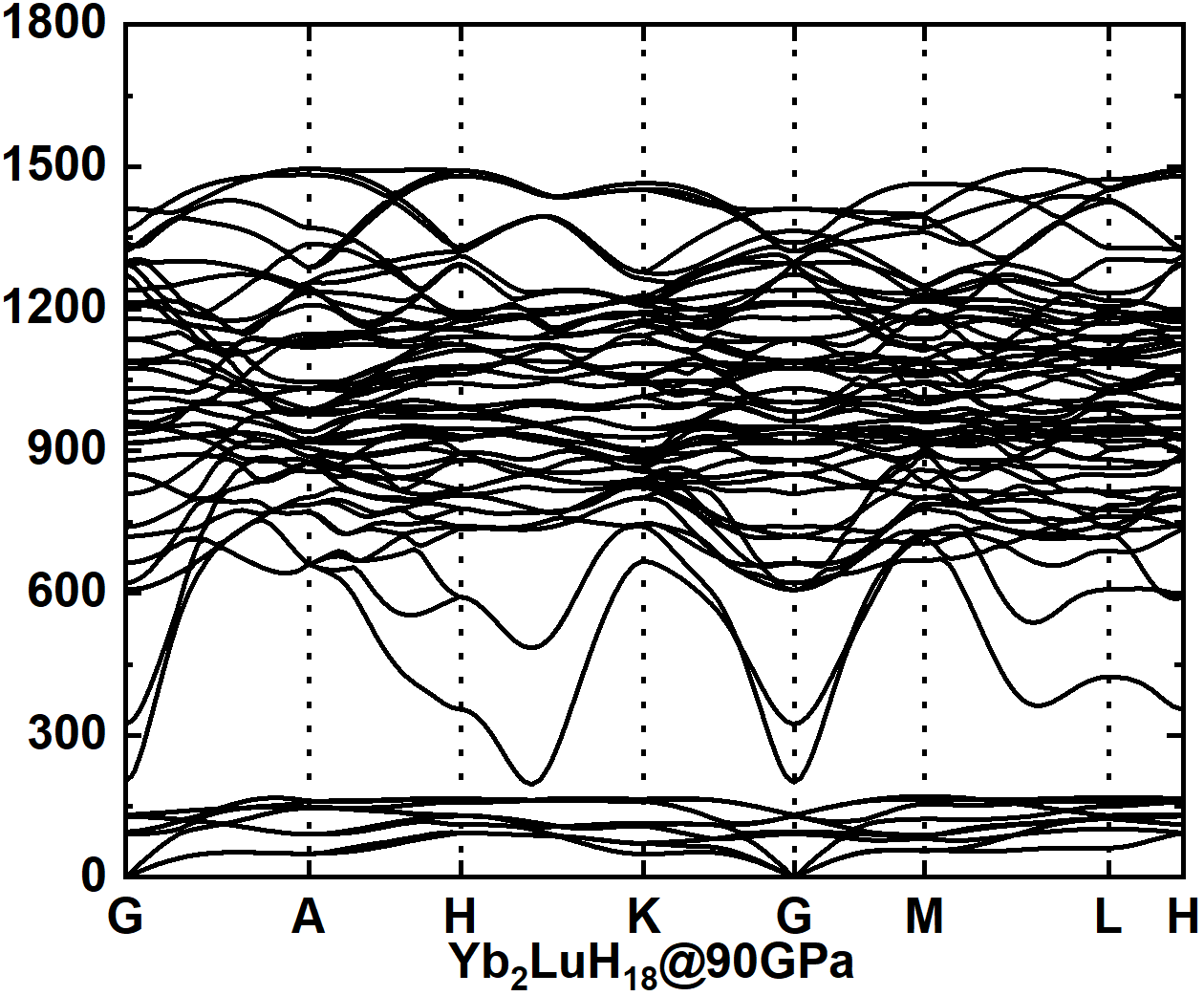


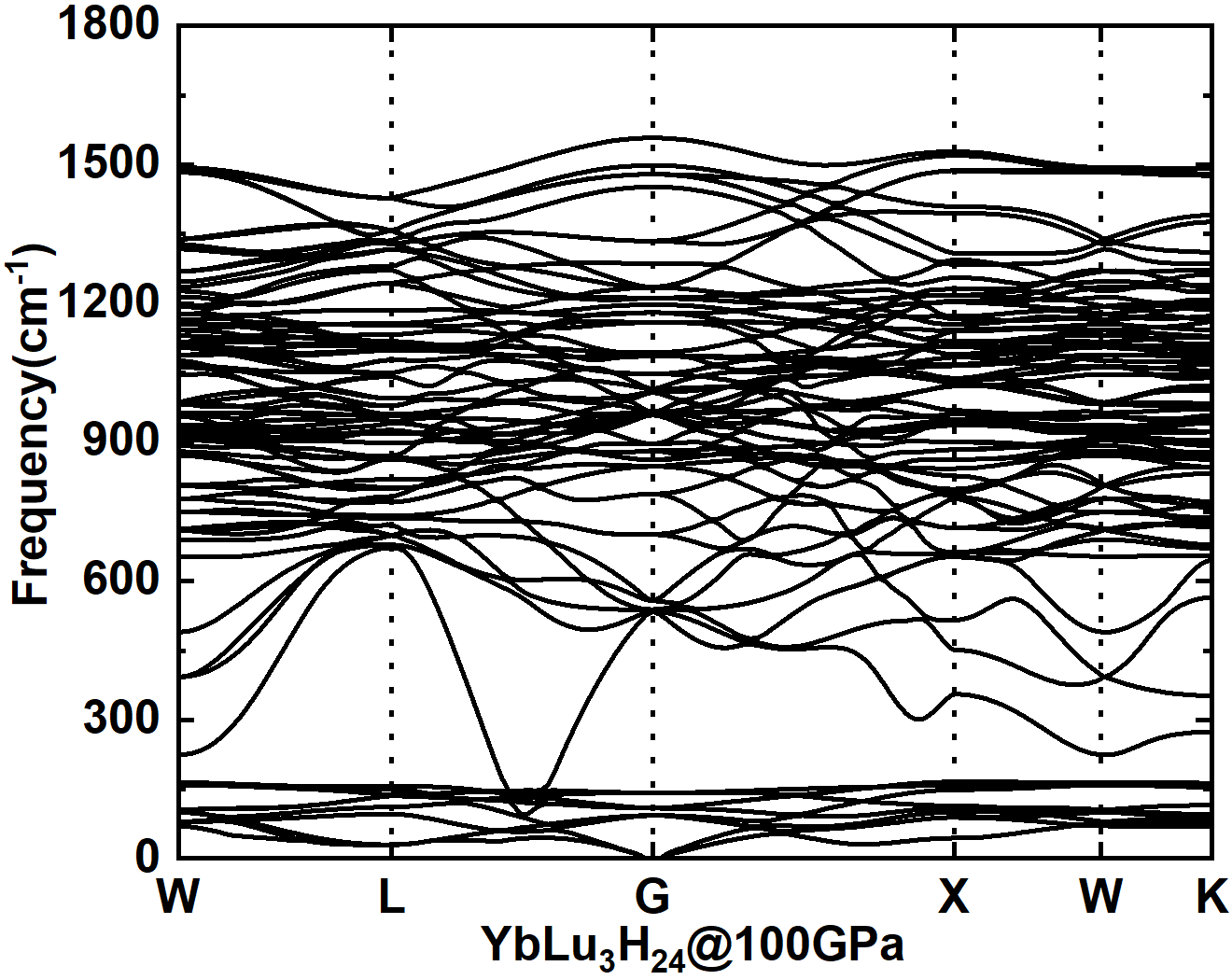

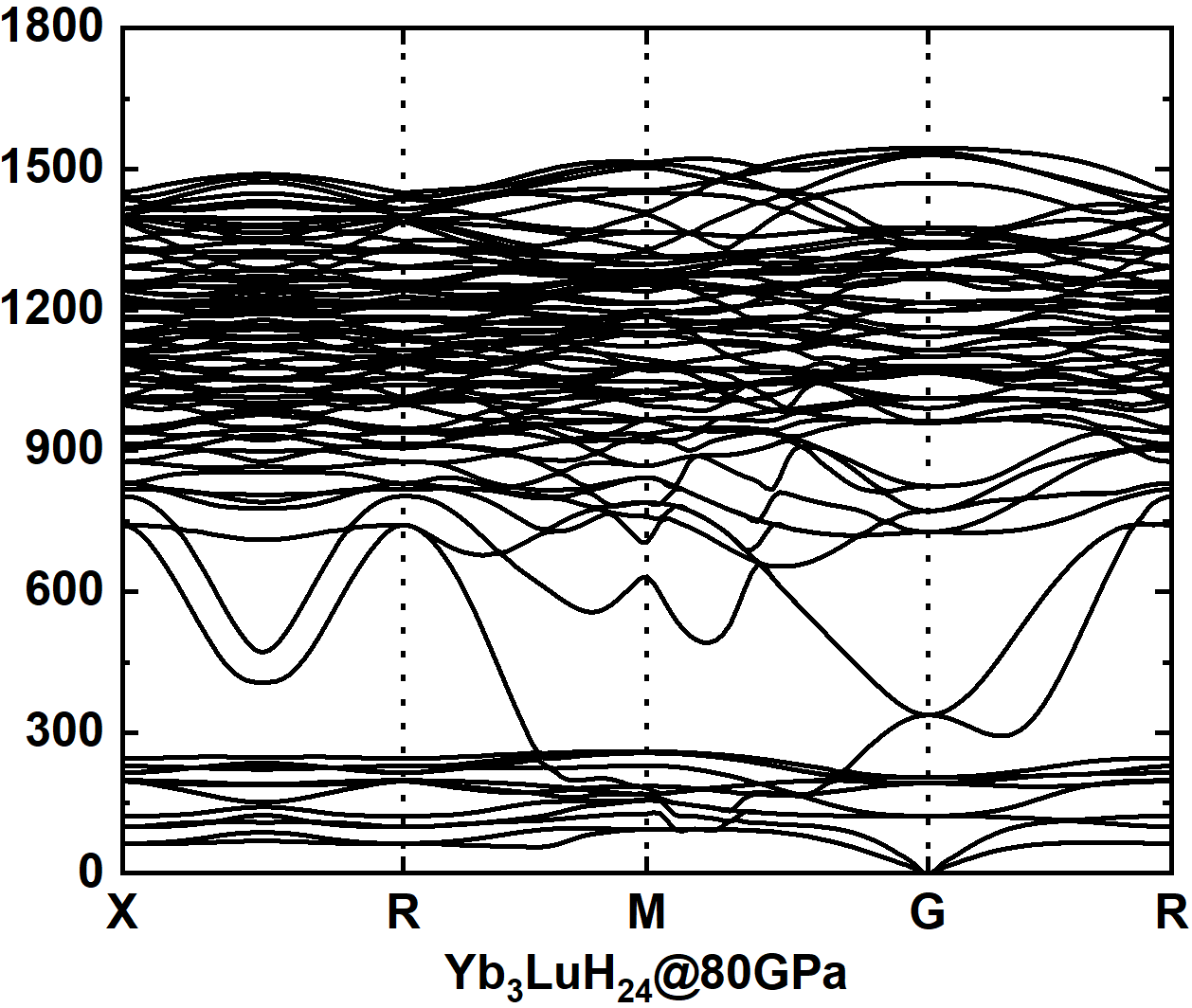


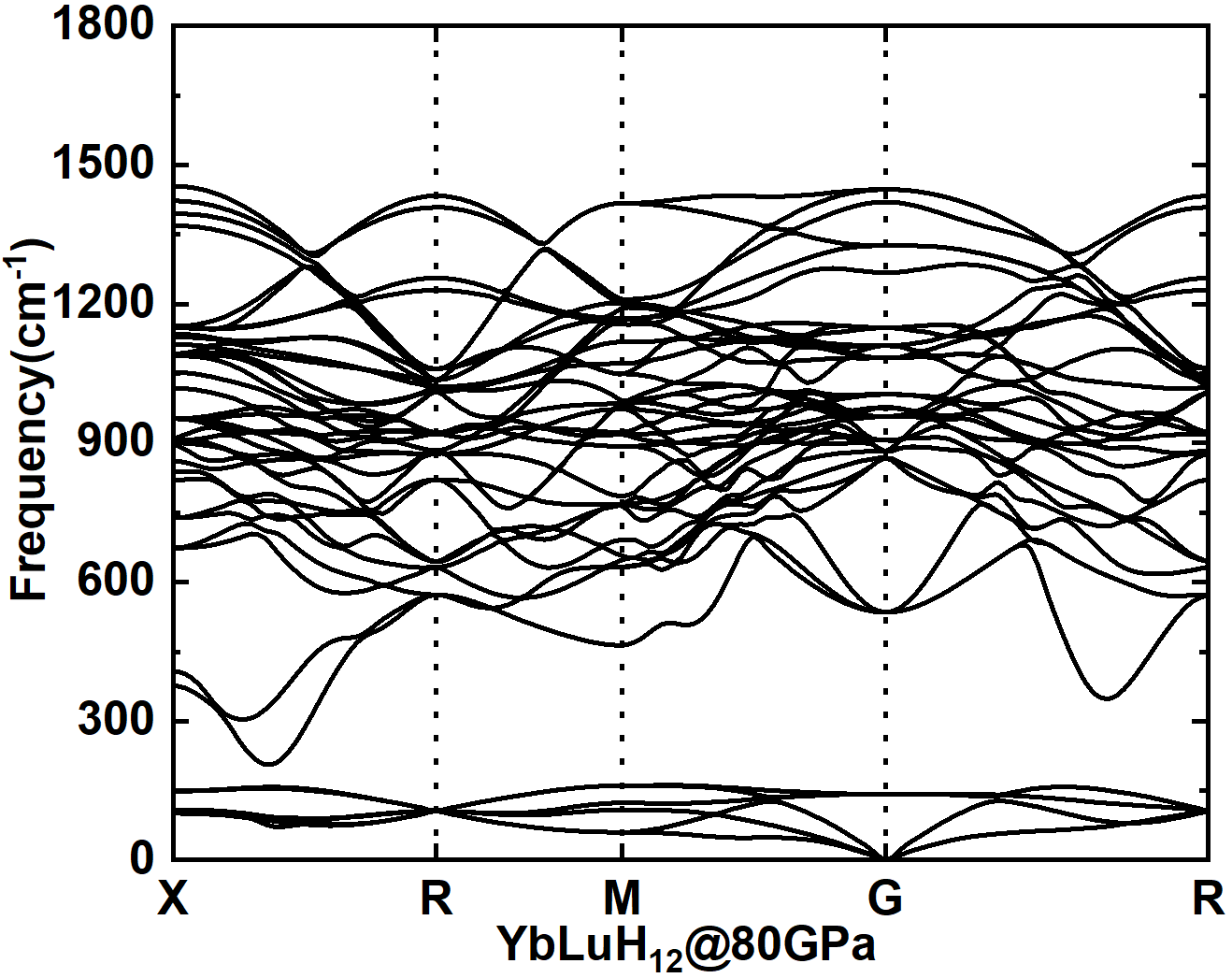

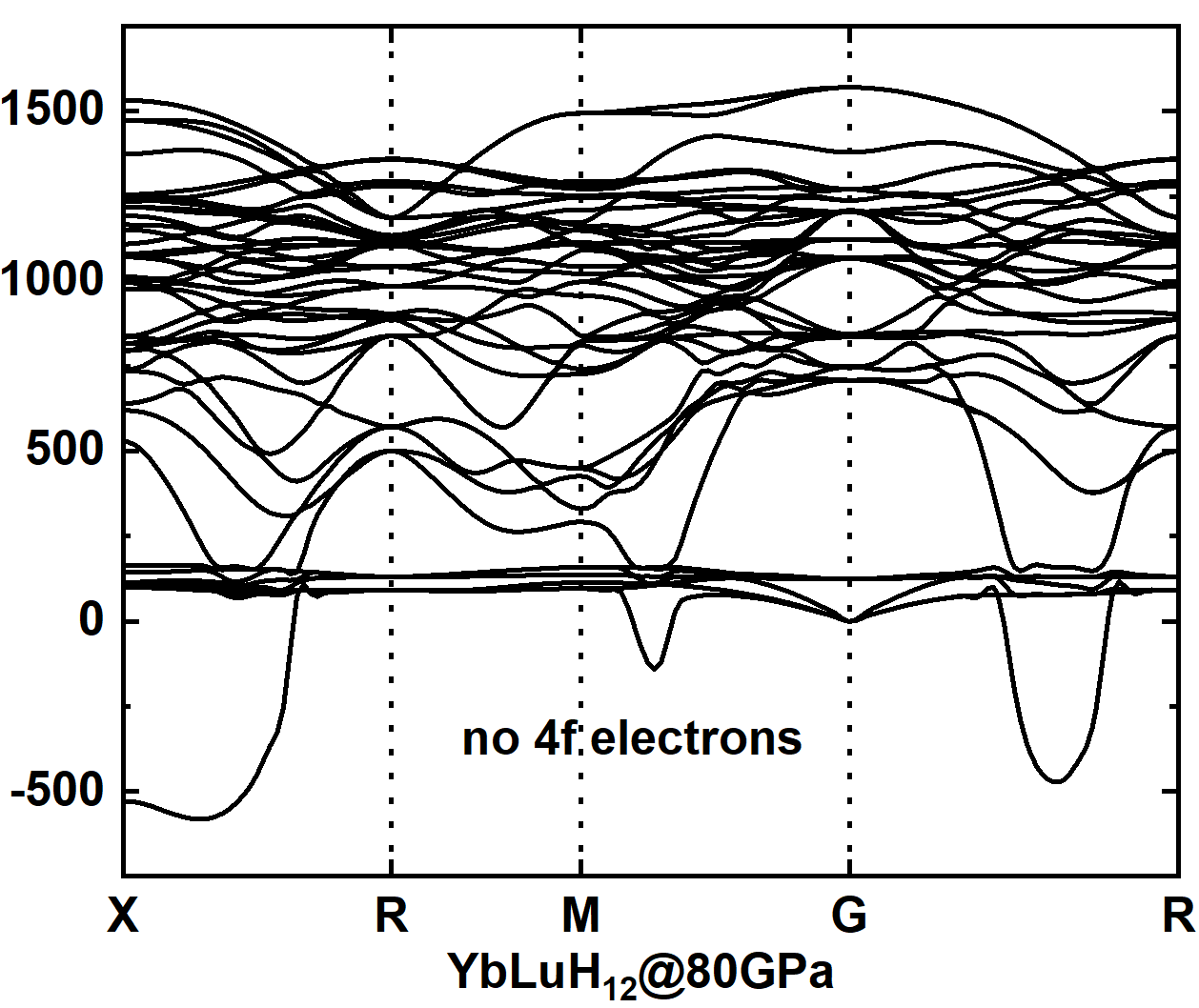


Fig. S15 The phonon band structure of YbLu_2_H_18_, Yb_2_LuH_18_, YbLu_3_H_24_, Yb_3_LuH_24_ and YbLuH_12_ under their minimum dynamically stable pressures, respectively. The last phonon band structure of YbLuH_12_ is calculated without considering the interactions of 4f electrons with valence electrons.


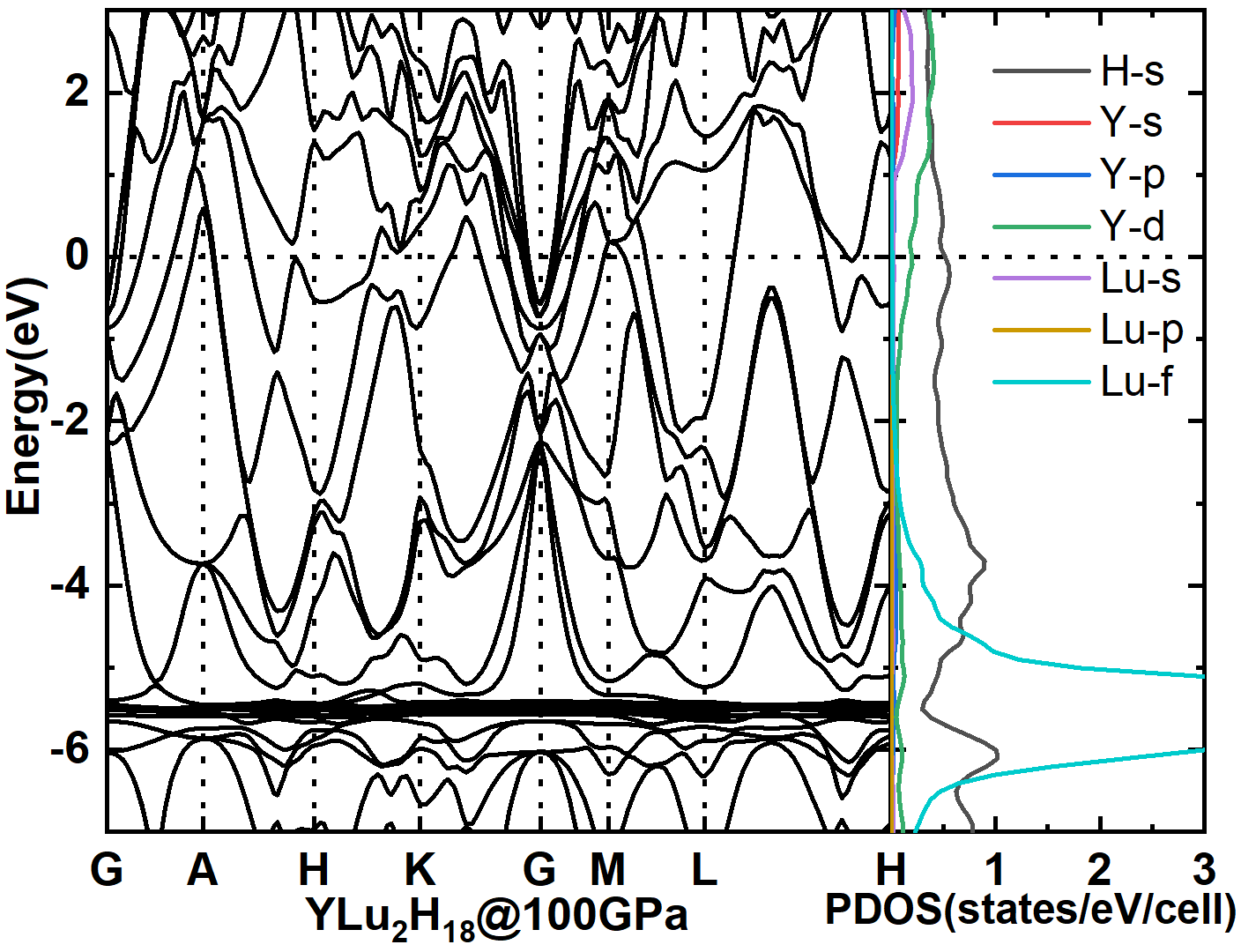

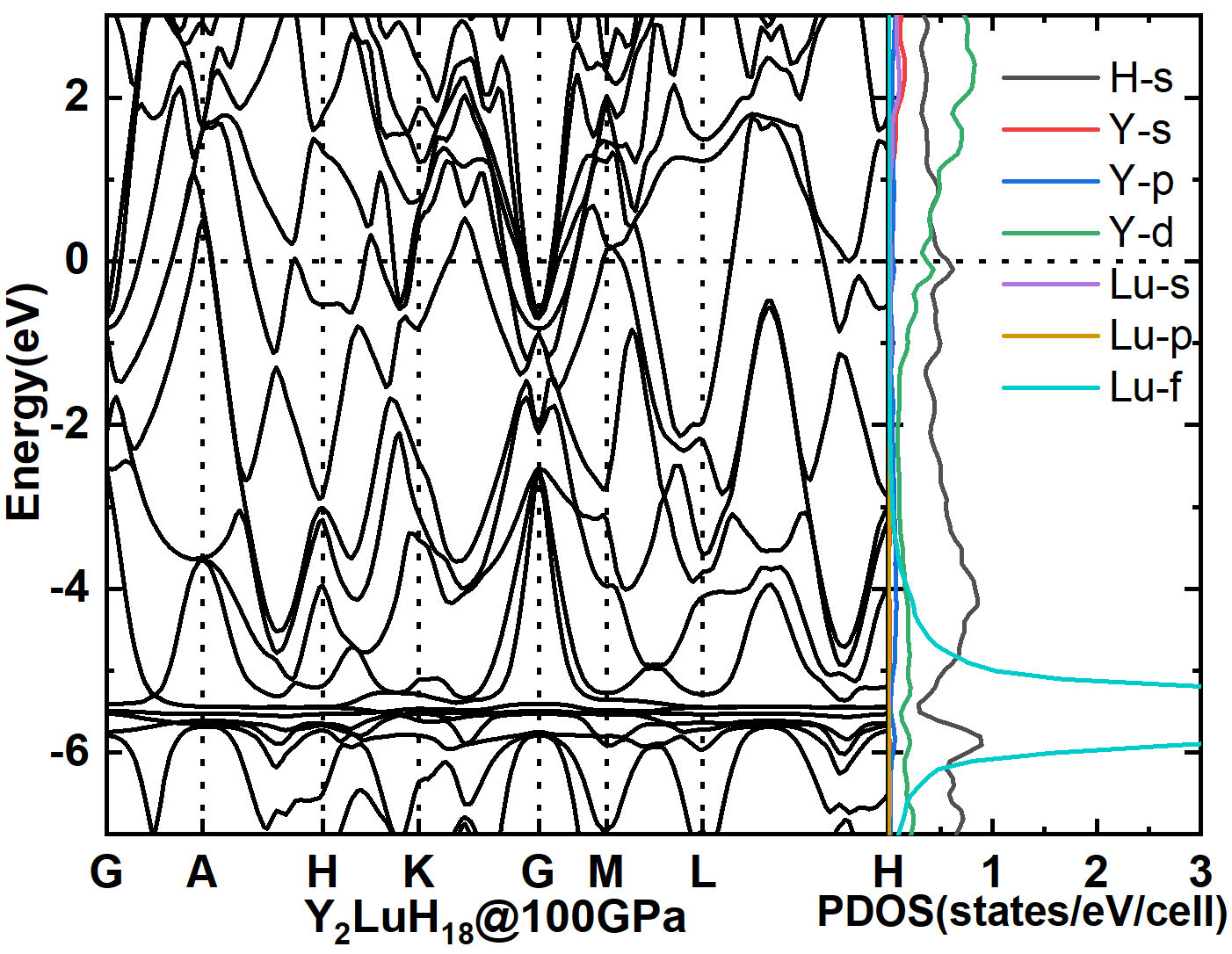


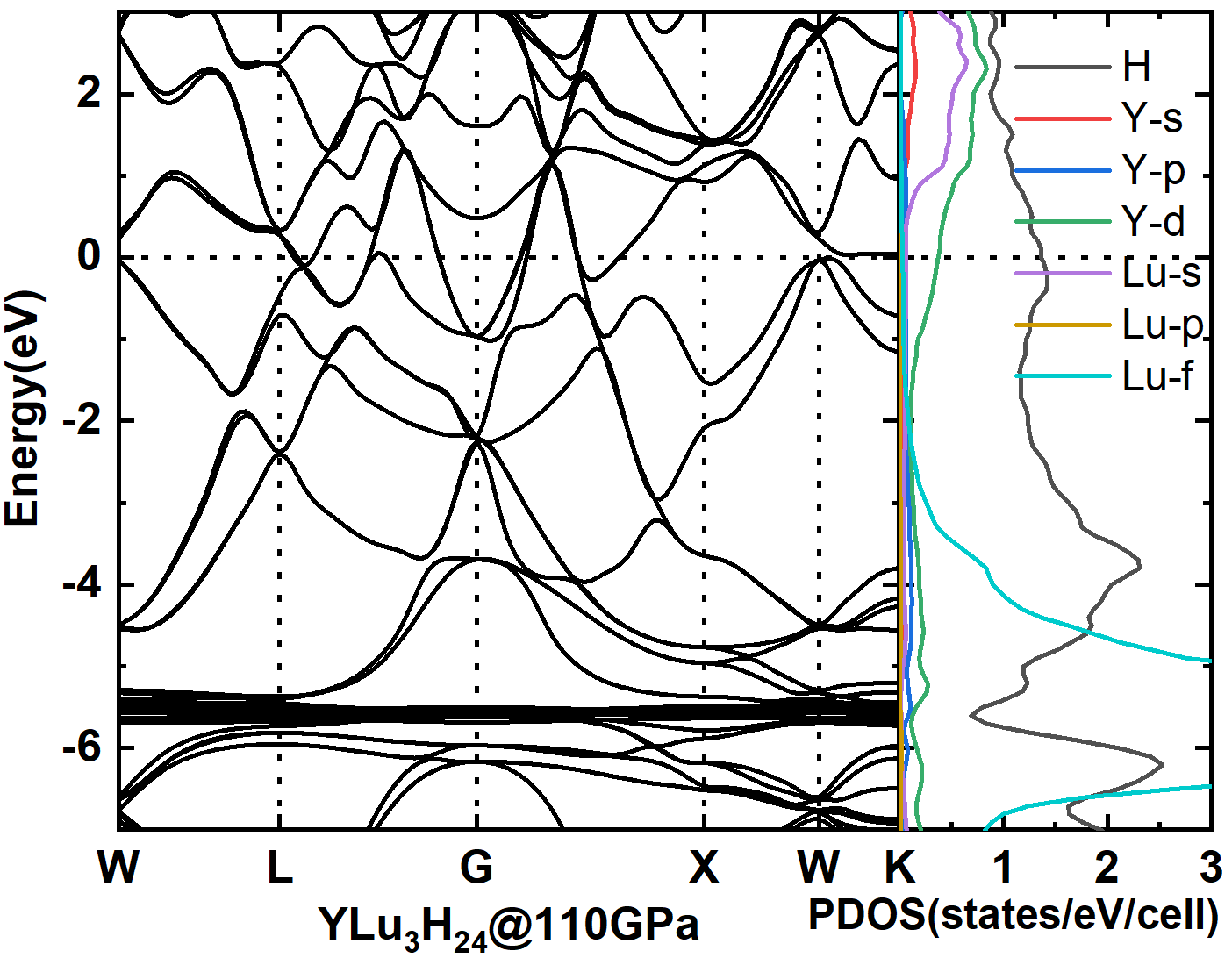

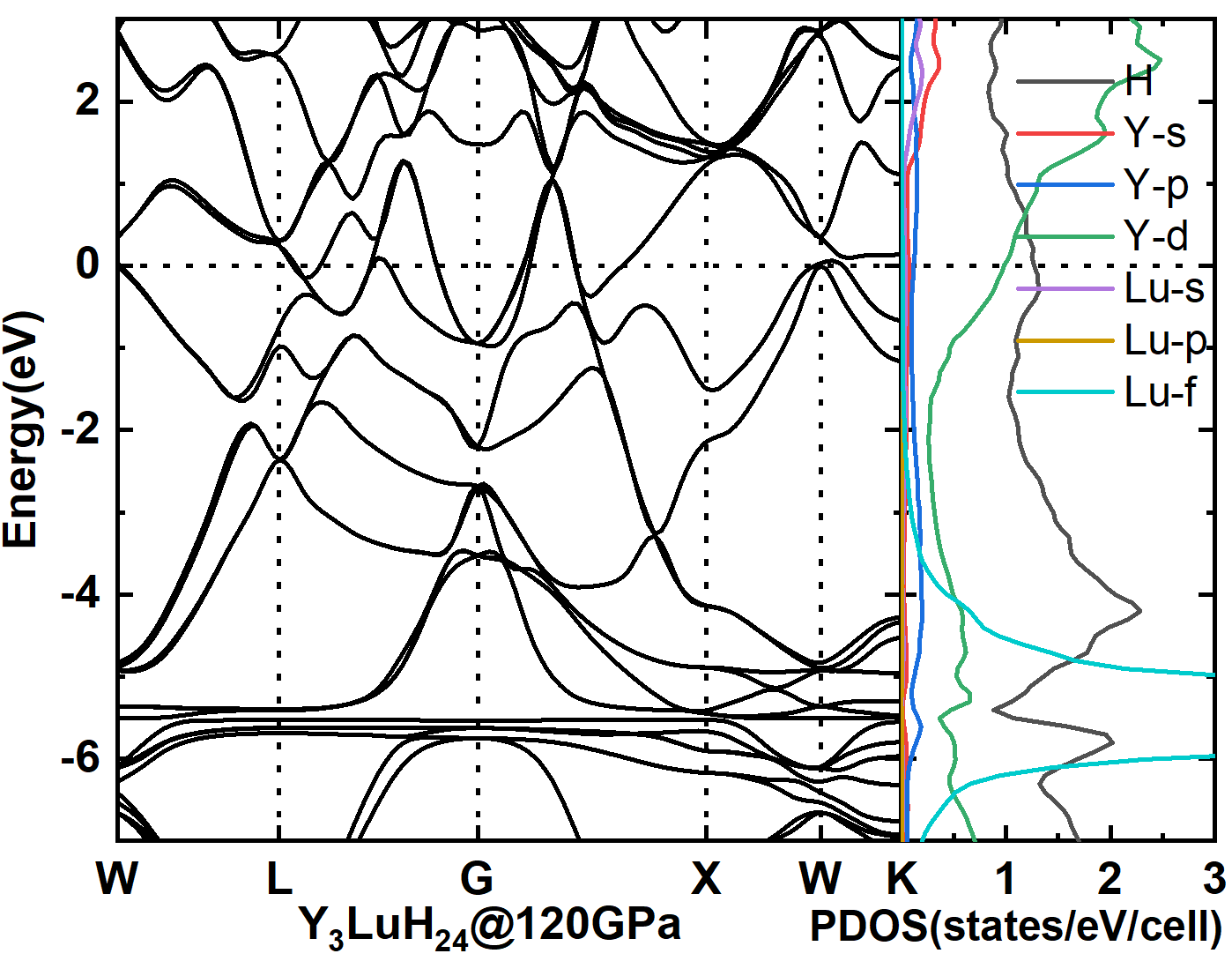


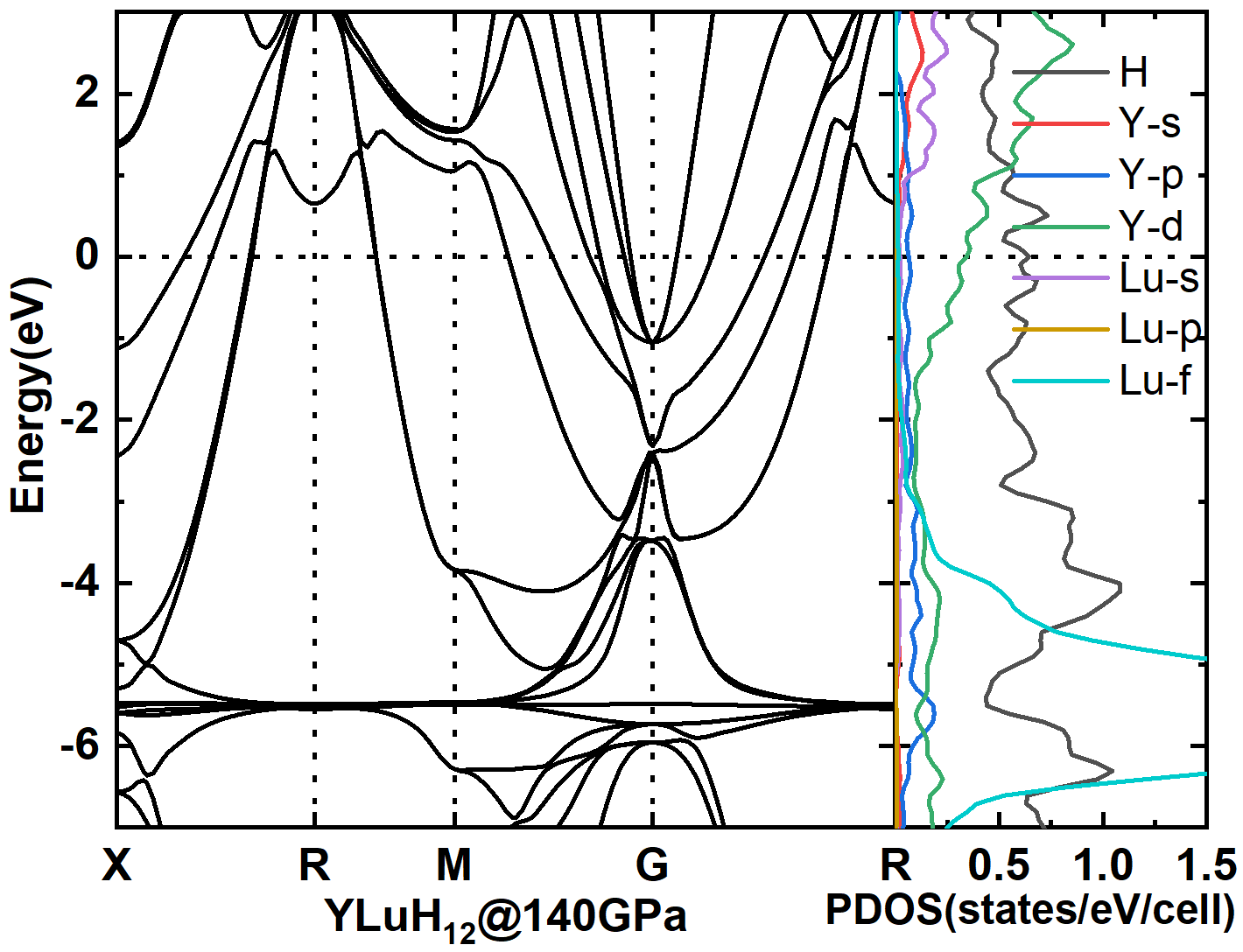


Fig. S16 Electronic band structures and projected density of electronic states of YLu_2_H_18_, Y_2_LuH_18_, YLu_3_H_24_, Y_3_LuH_24_ and YLuH_12_ under their minimum dynamically stable pressures, respectively.


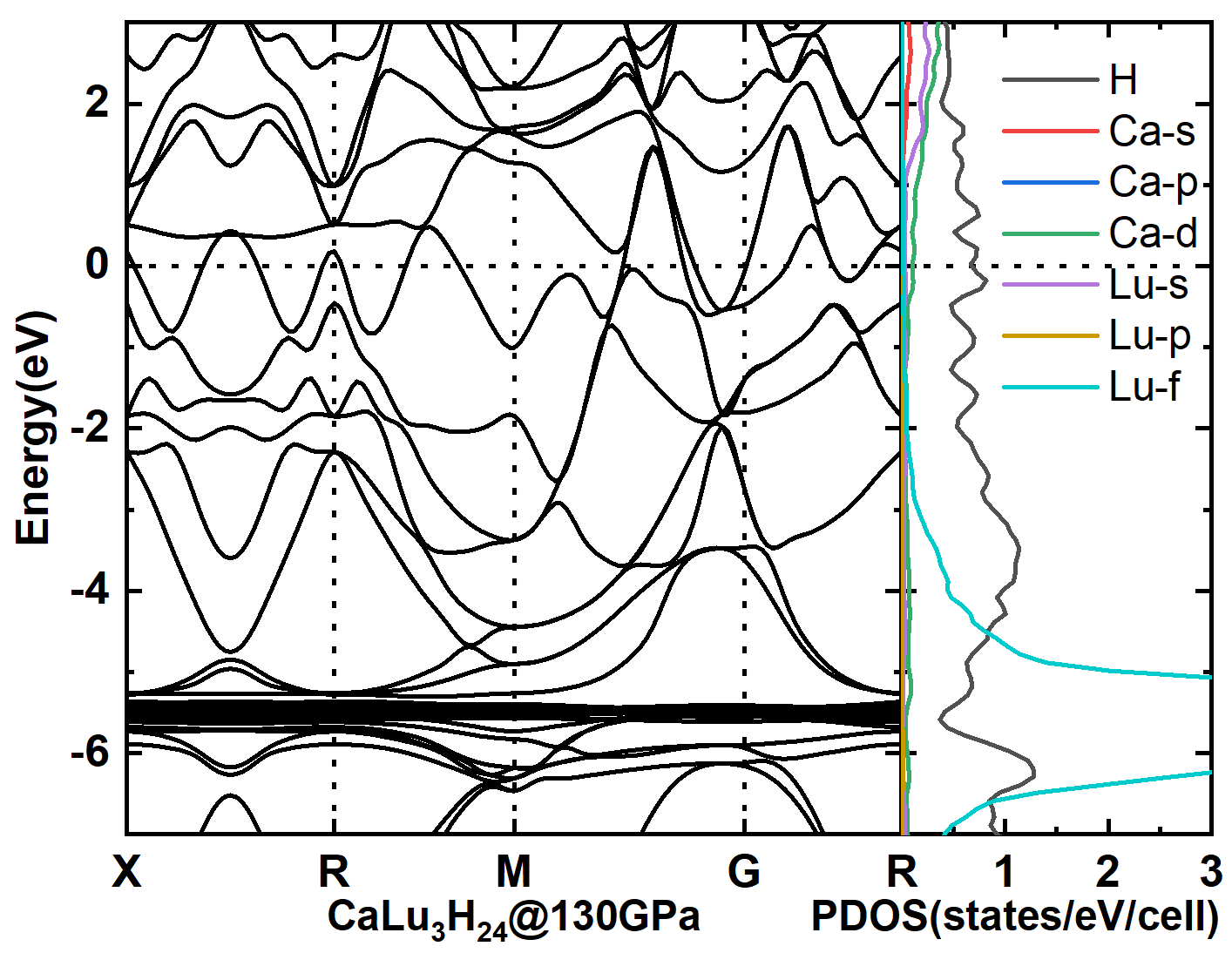

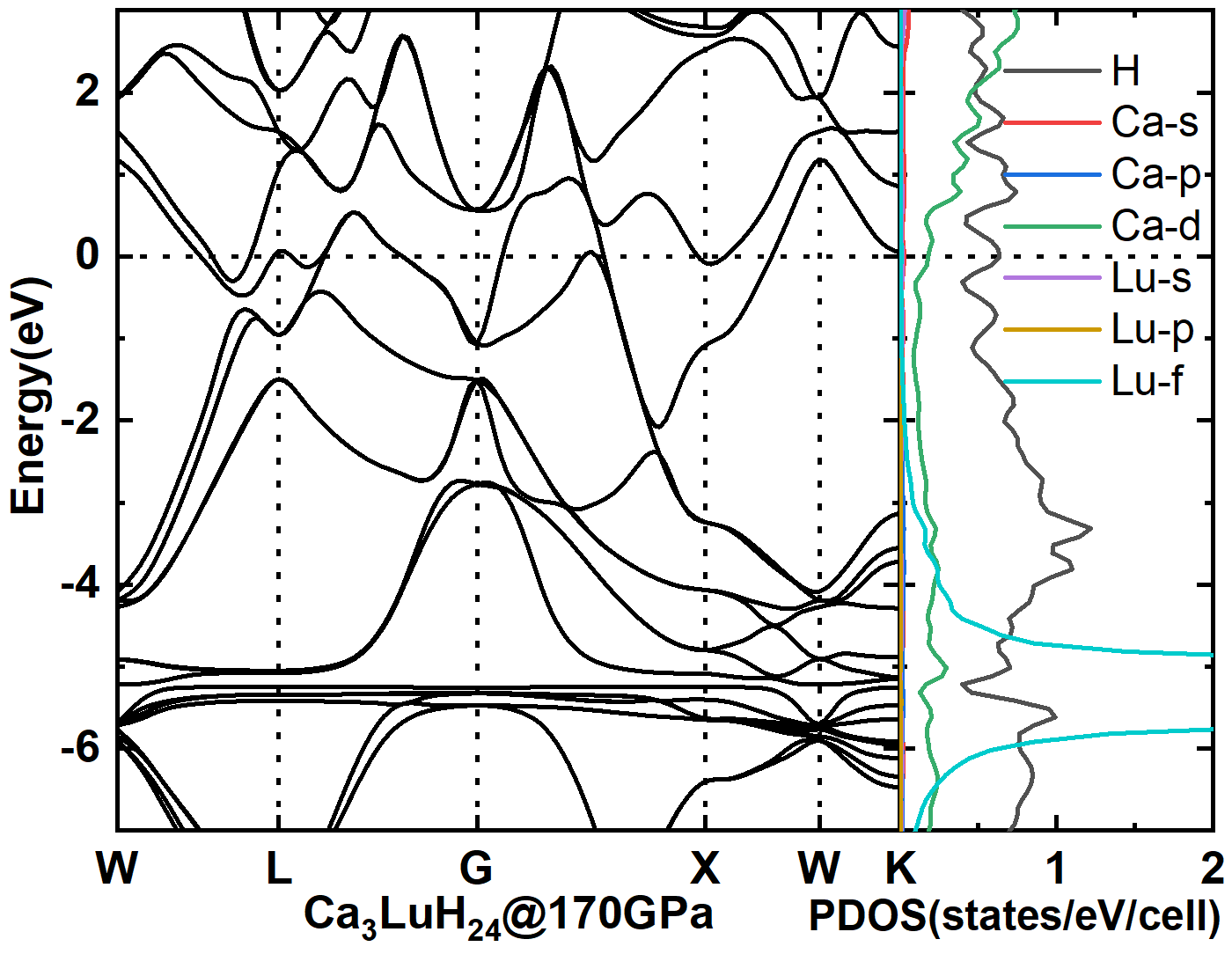


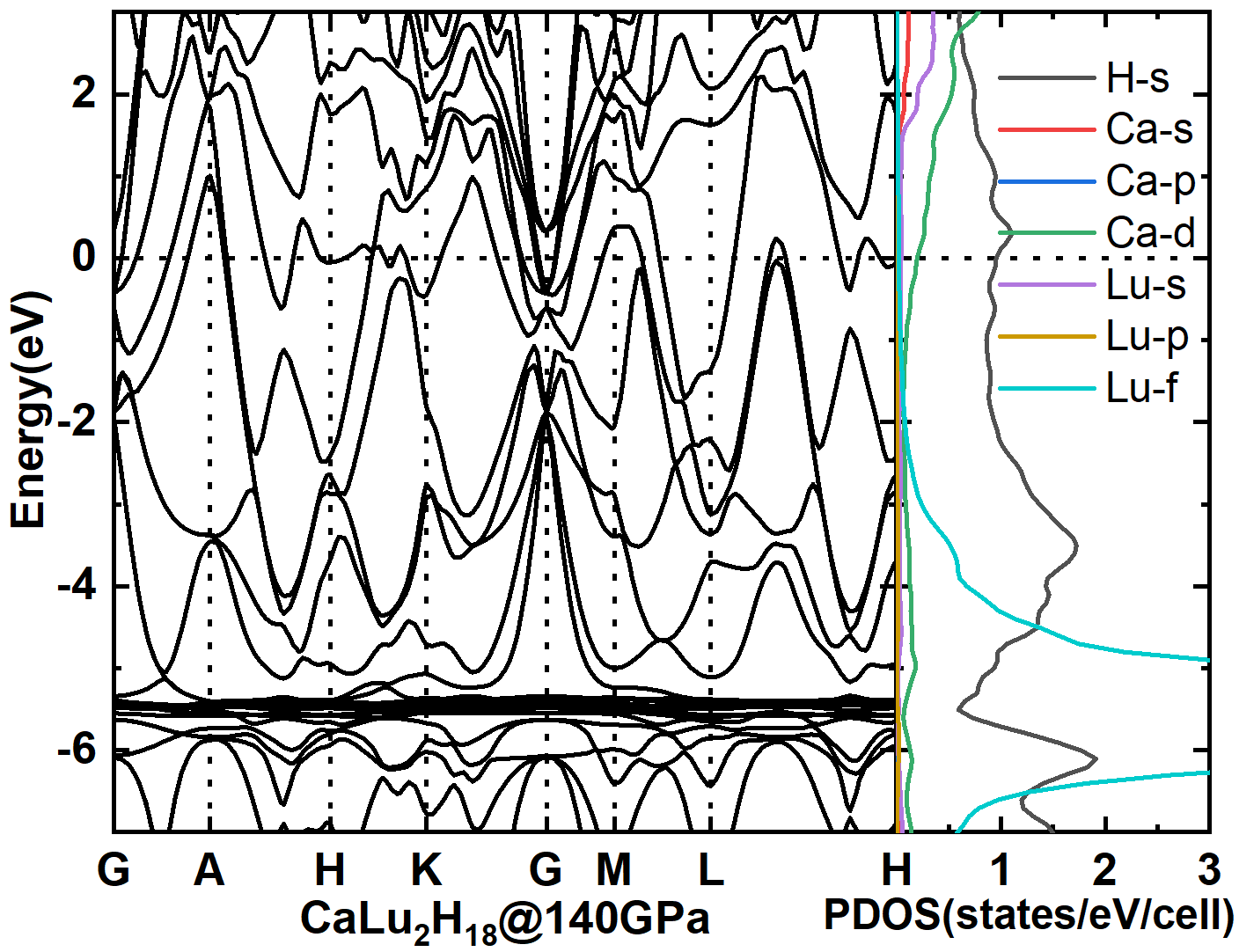

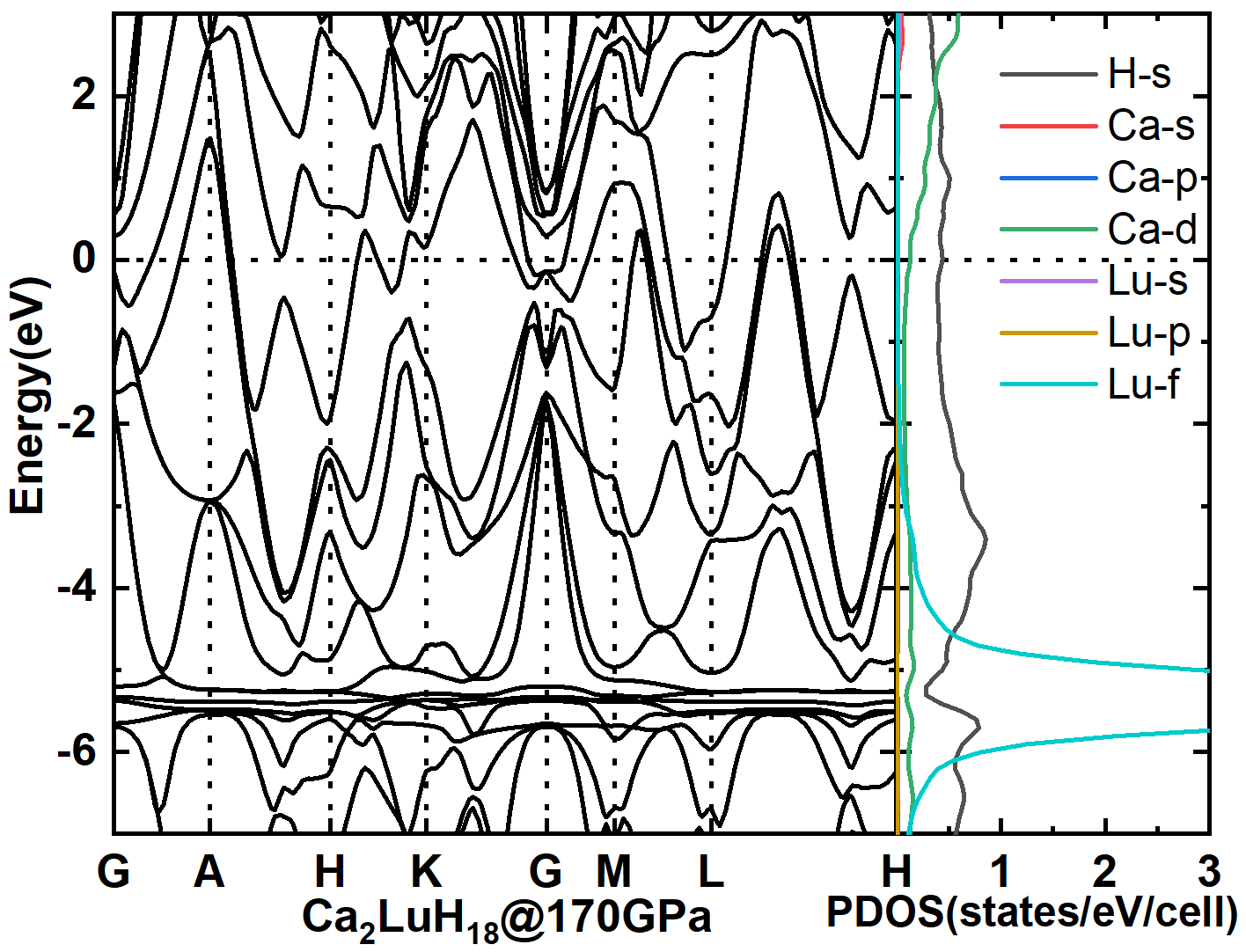


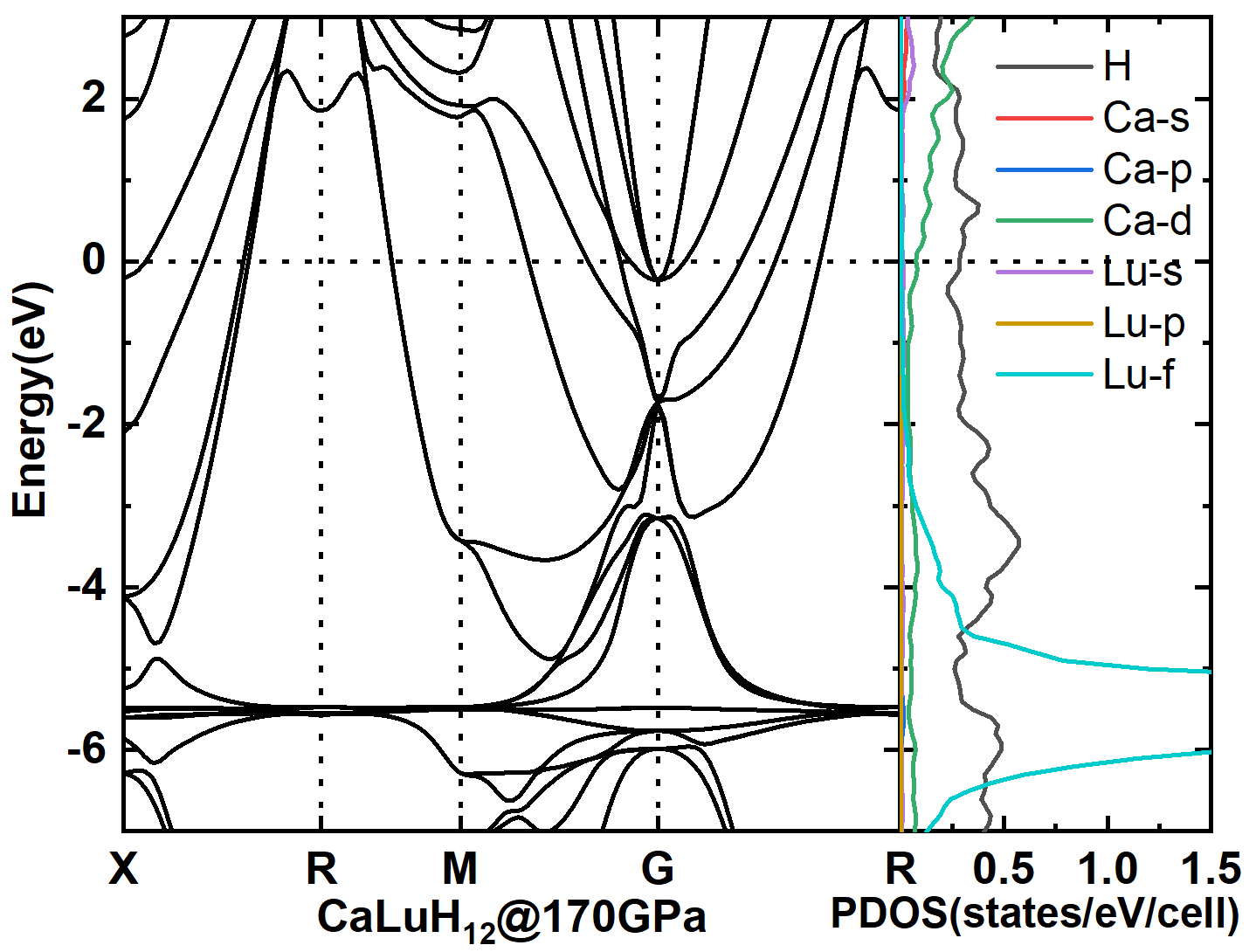


Fig. S17 Electronic band structures and projected density of electronic states of Ca_3_LuH_24_, CaLu_3_H_24_, CaLu_2_H_18_, Ca_2_LuH_18_ and CaLuH_12_ under their minimum dynamically stable pressures, respectively.


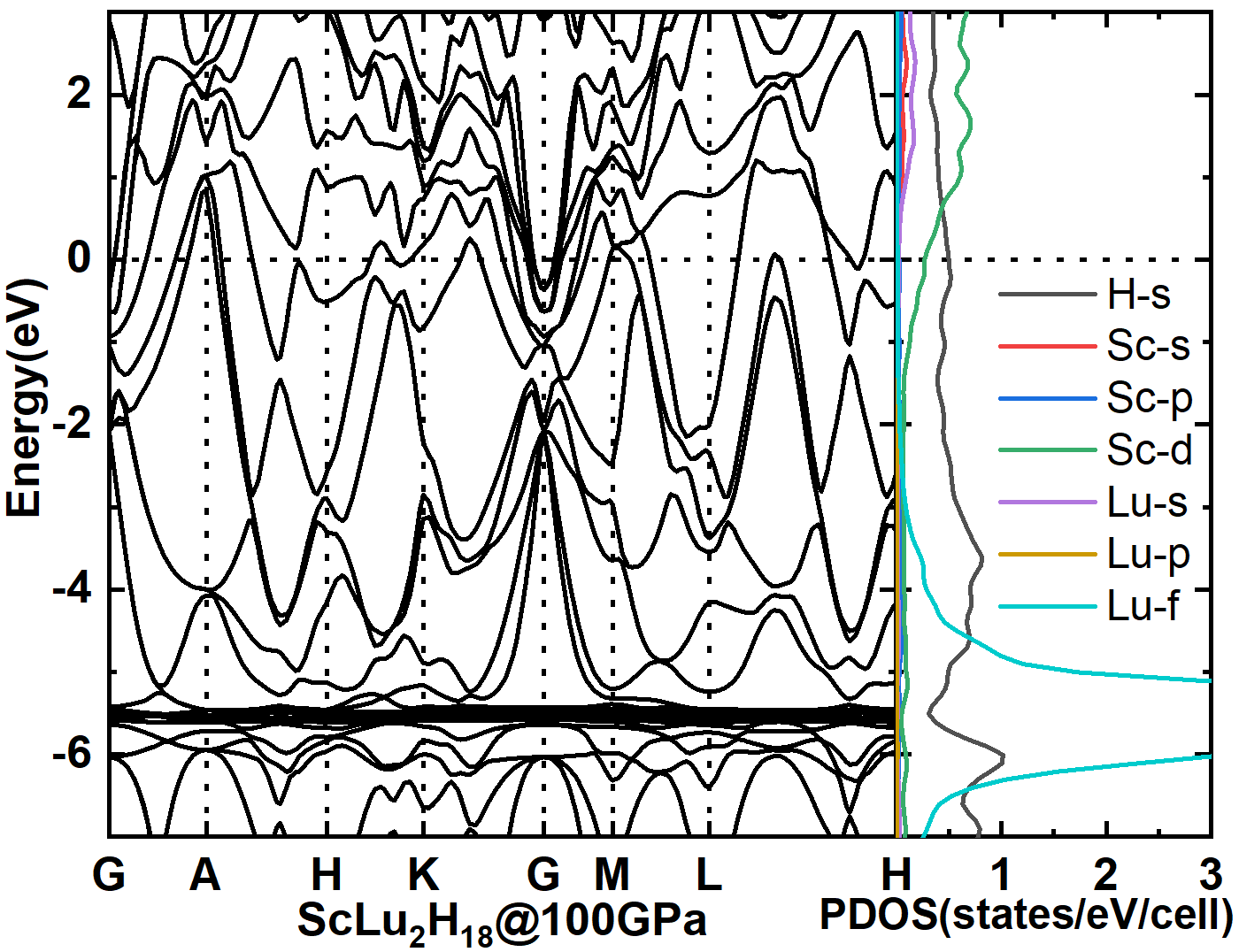

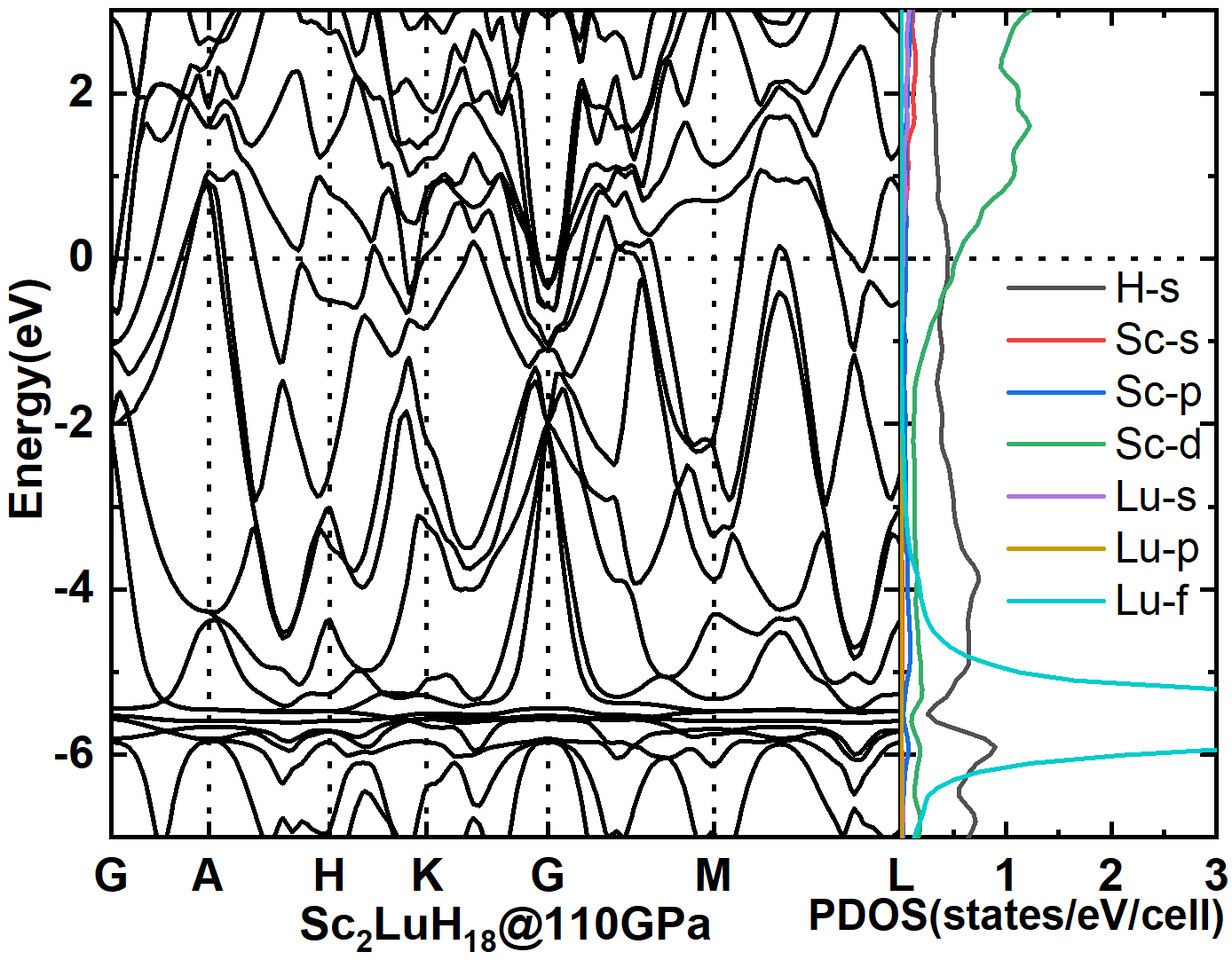


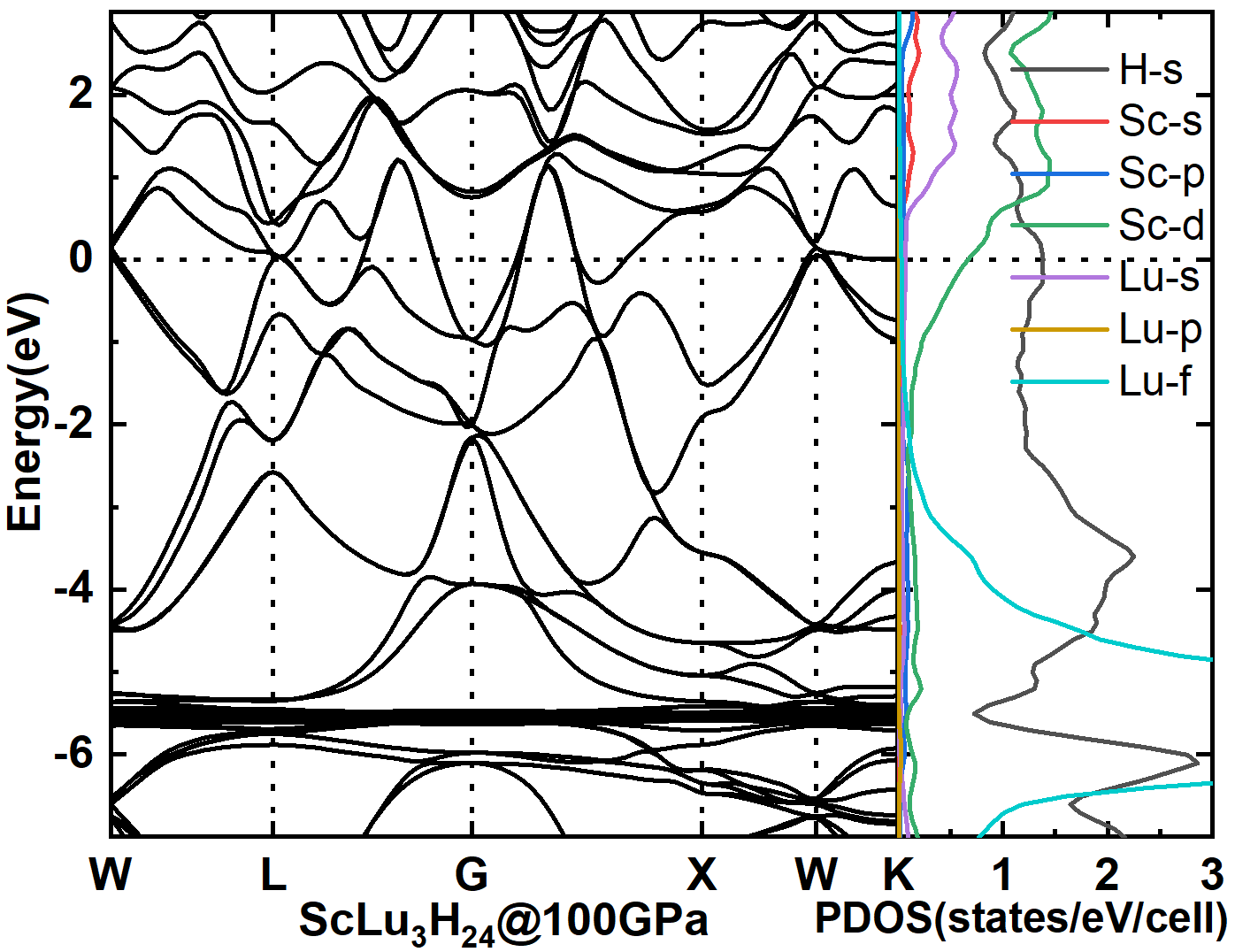

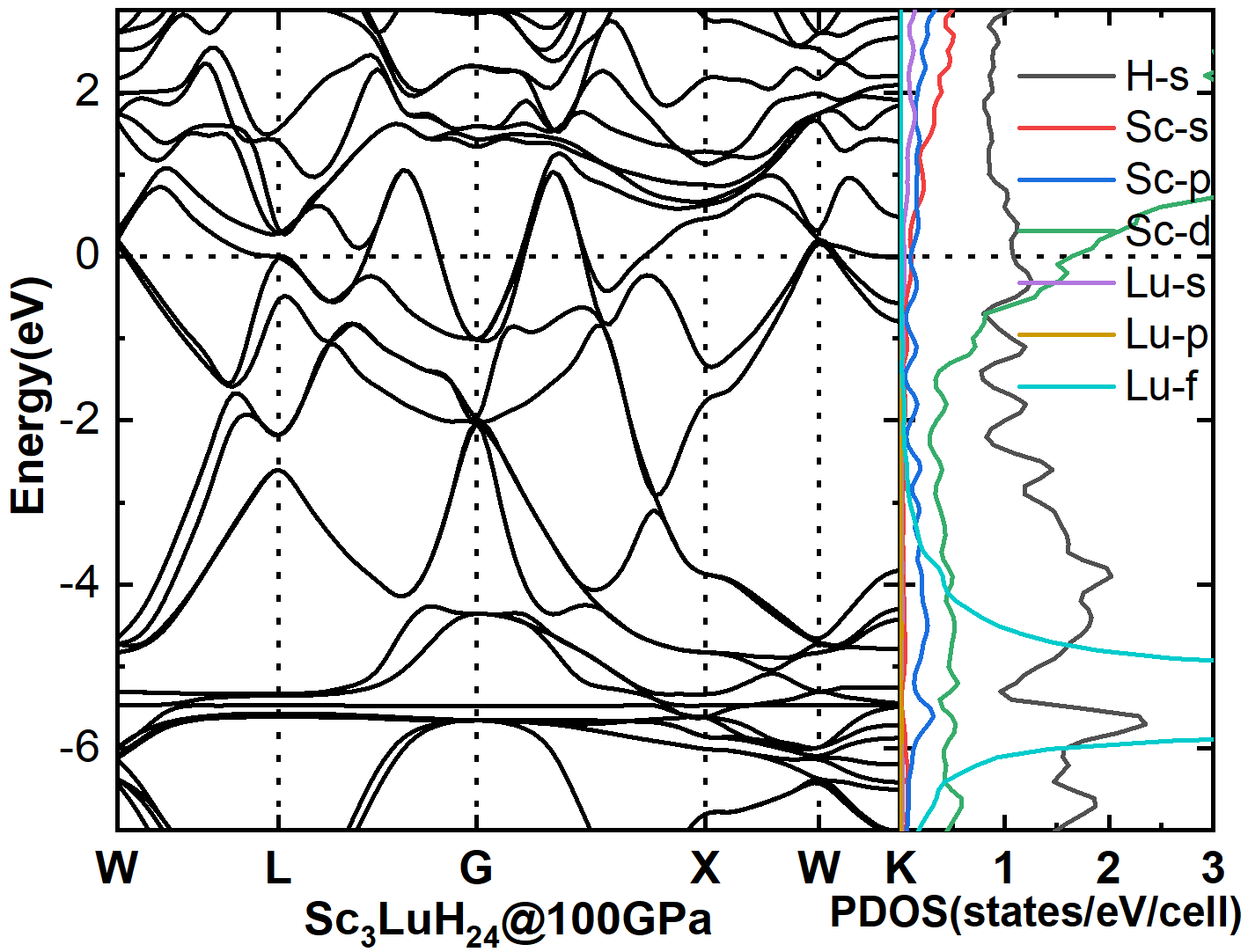


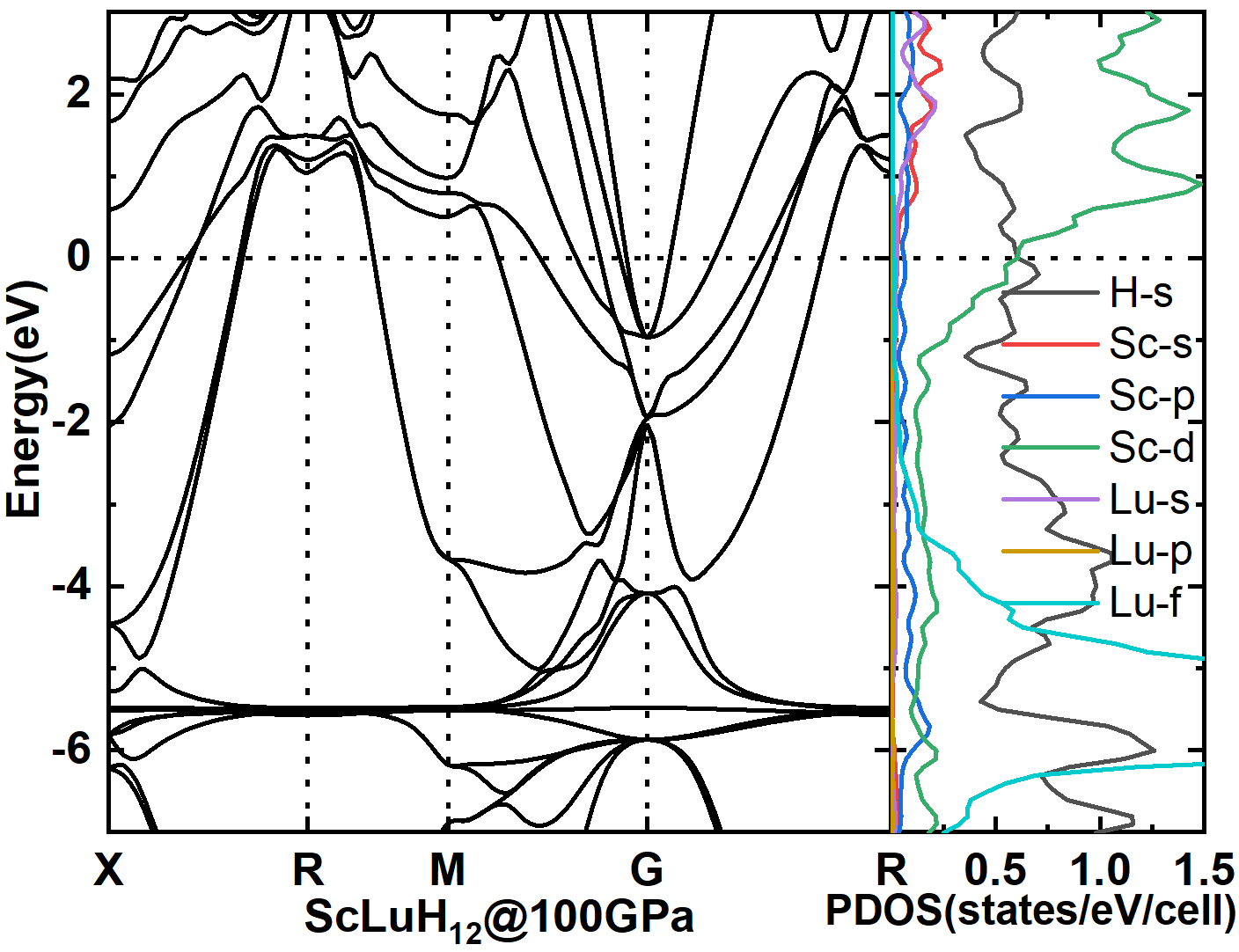


Fig. S18 Electronic band structures and projected density of electronic states of ScLu_2_H_18_, Sc_2_LuH_18_, ScLu_3_H_24_, Sc_3_LuH_24_ and ScLuH_12_ under their minimum dynamically stable pressures, respectively.


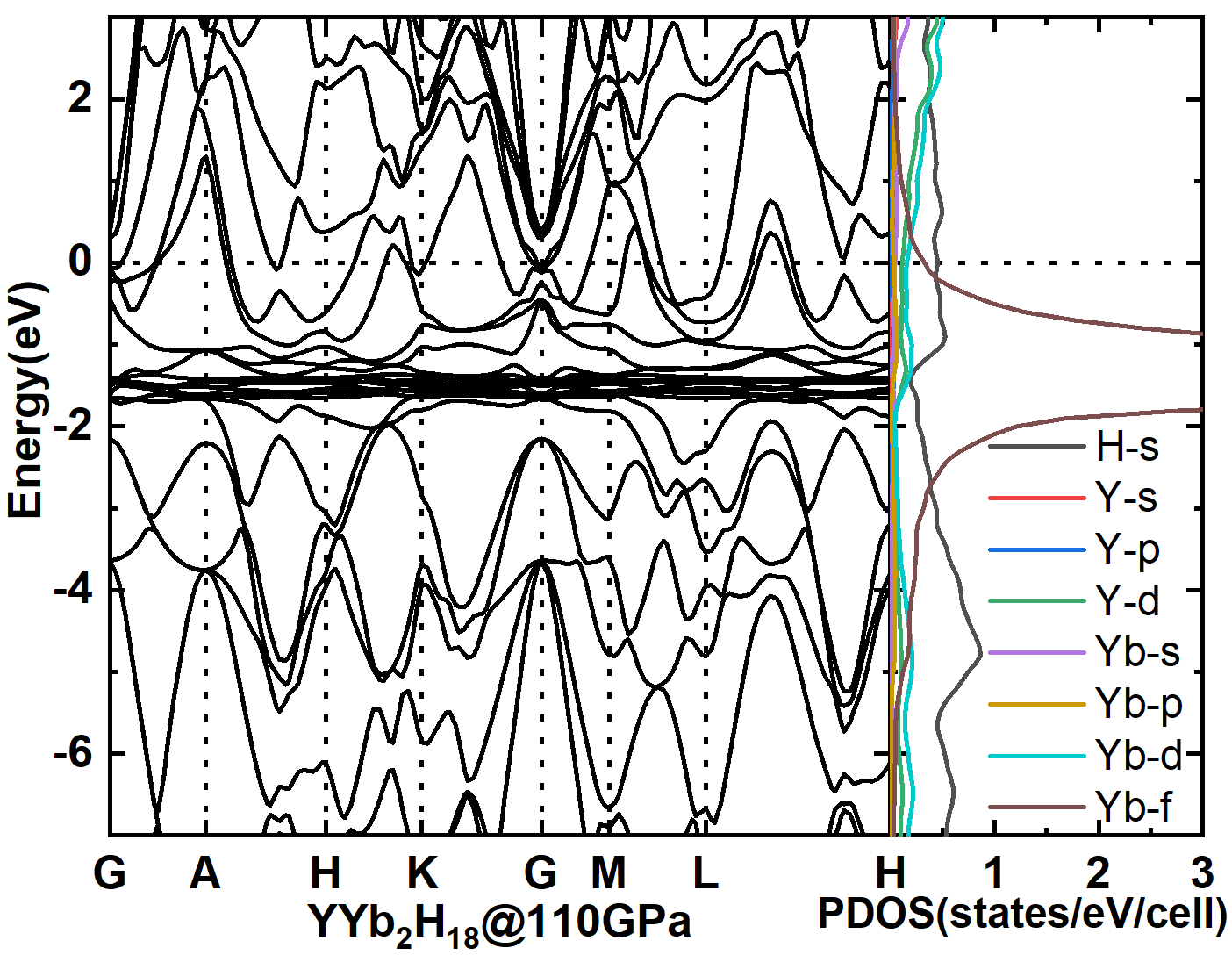

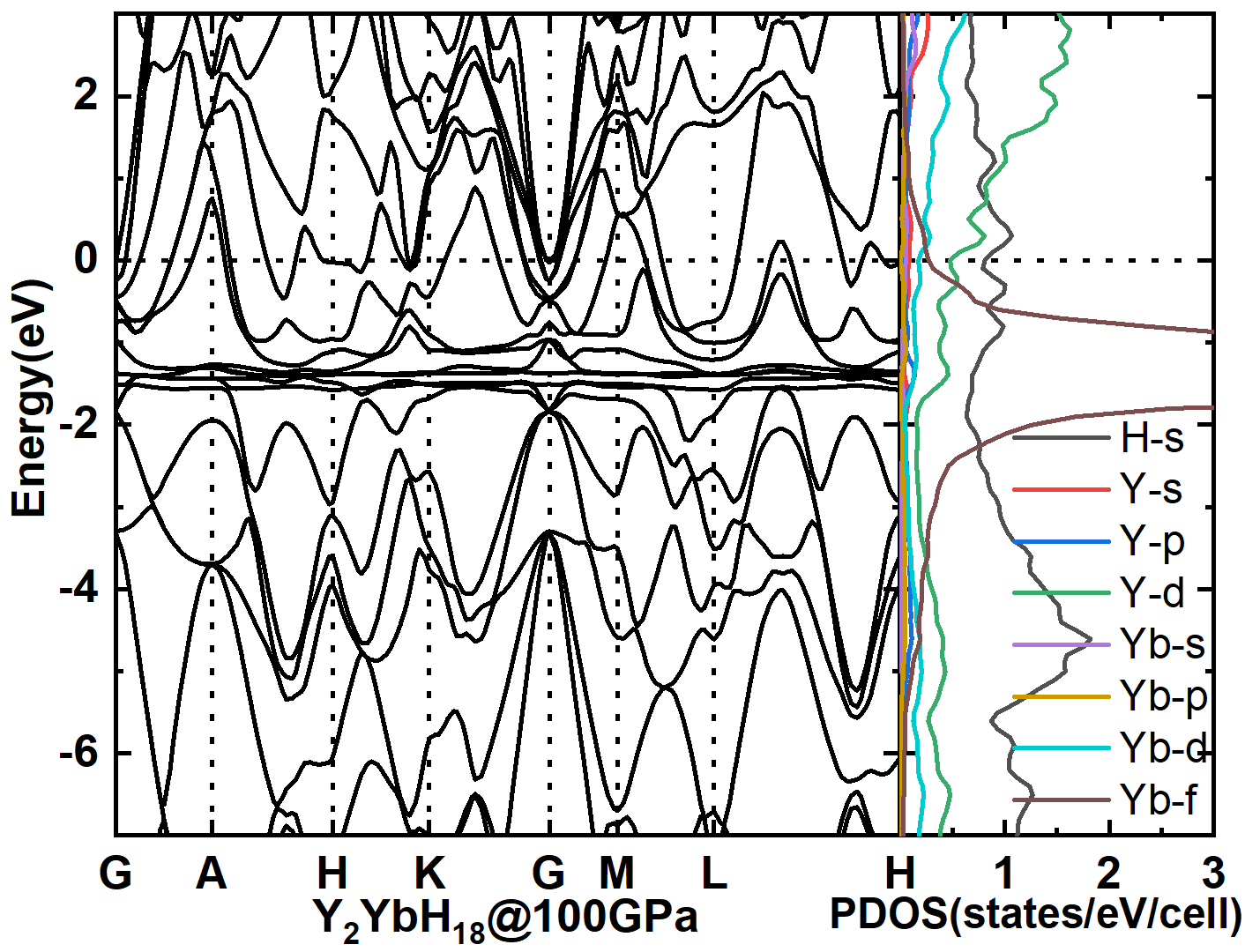


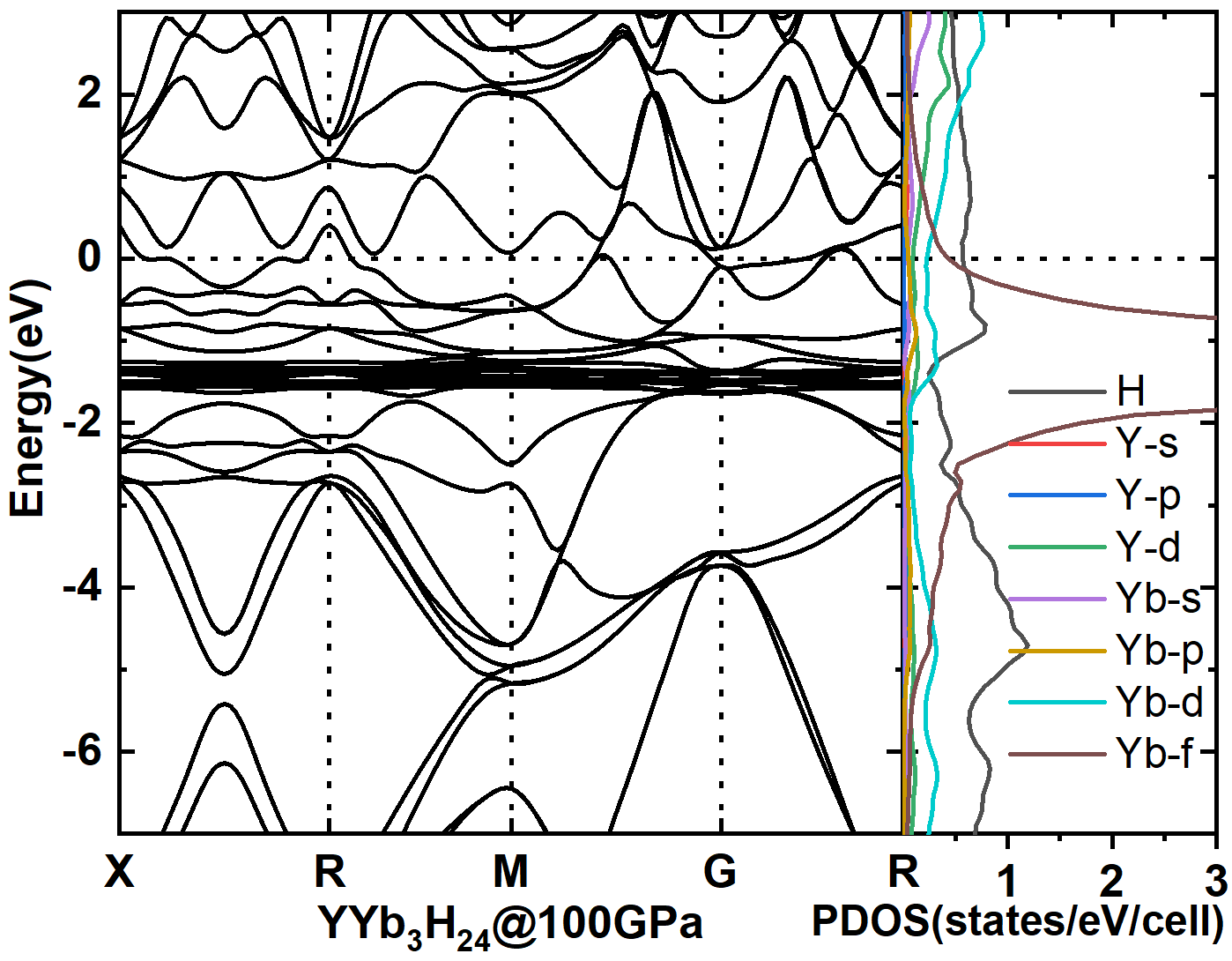

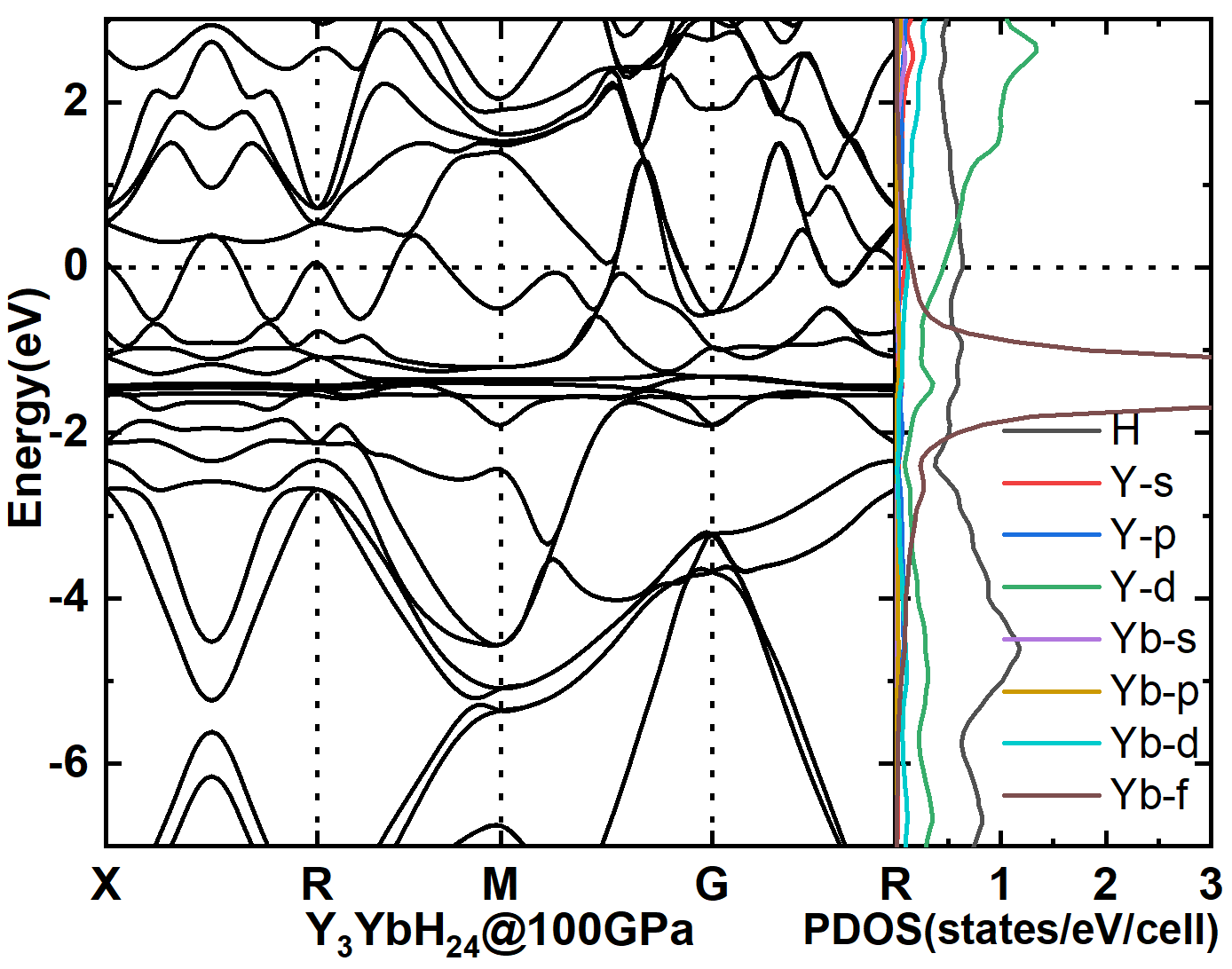


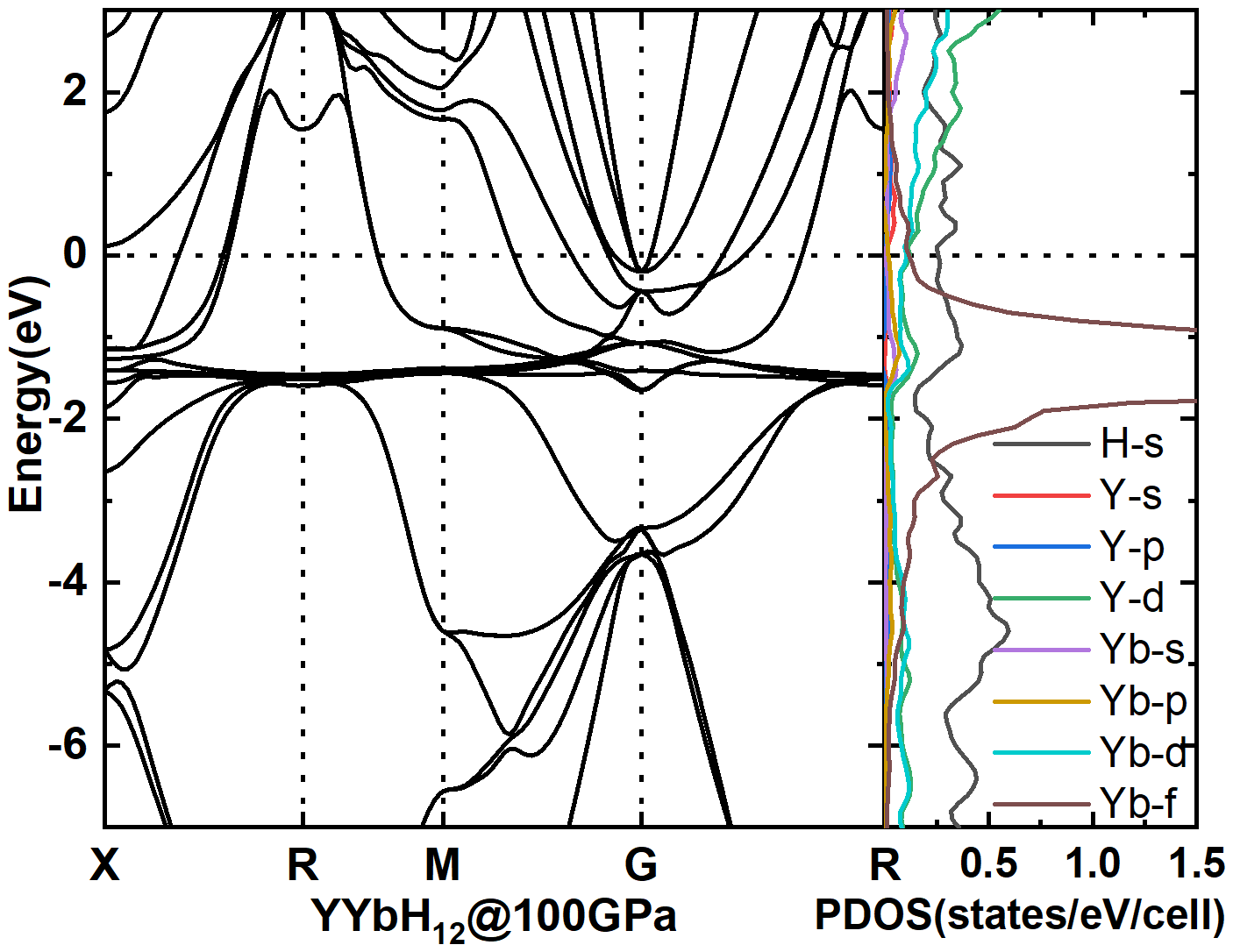


Fig. S19 Electronic band structures and projected density of electronic states of YYb_2_H_18_, Y_2_YbH_18_, YYb_3_H_24_, Y_3_YbH_24_ and YYbH_12_ under their minimum dynamically stable pressures, respectively.


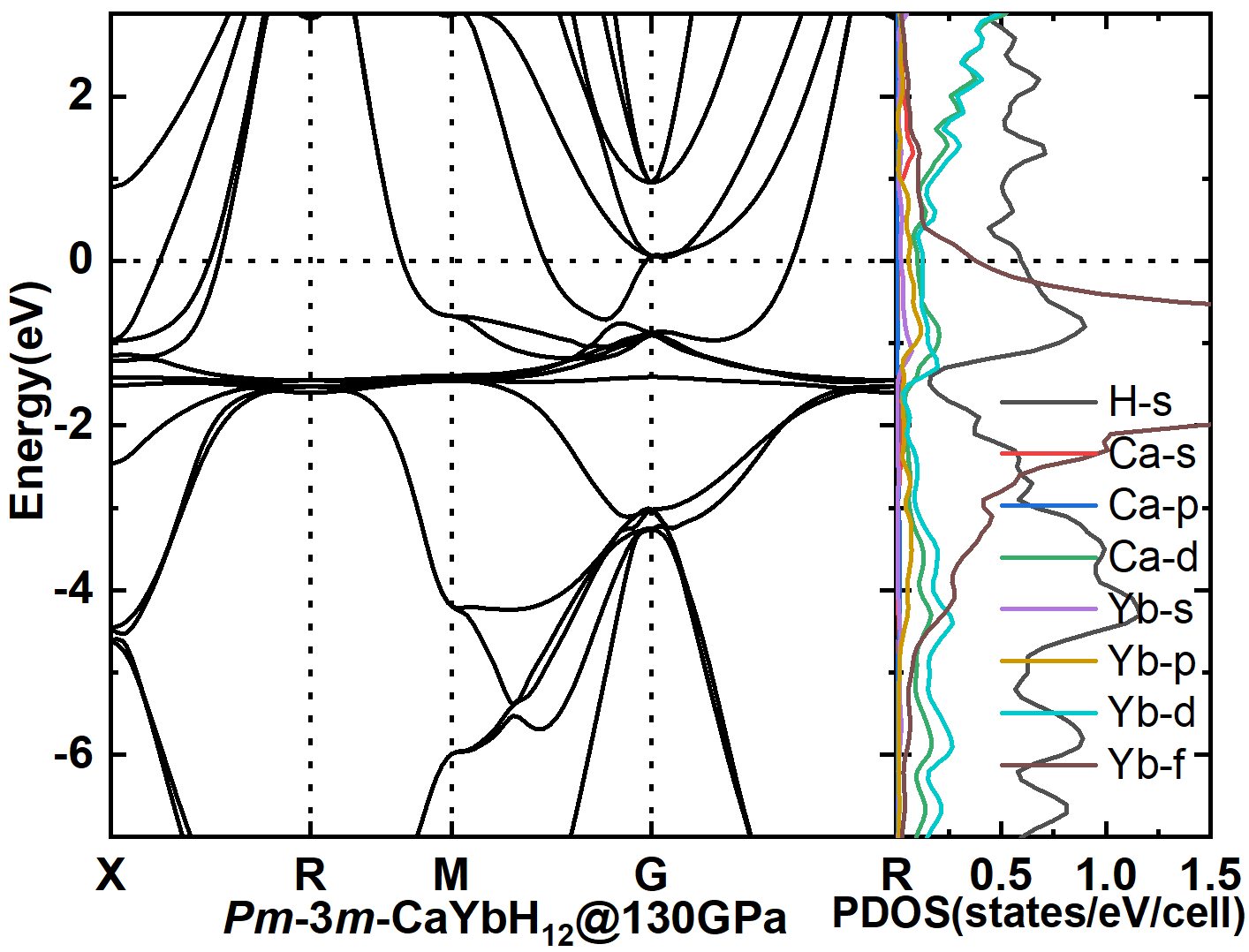

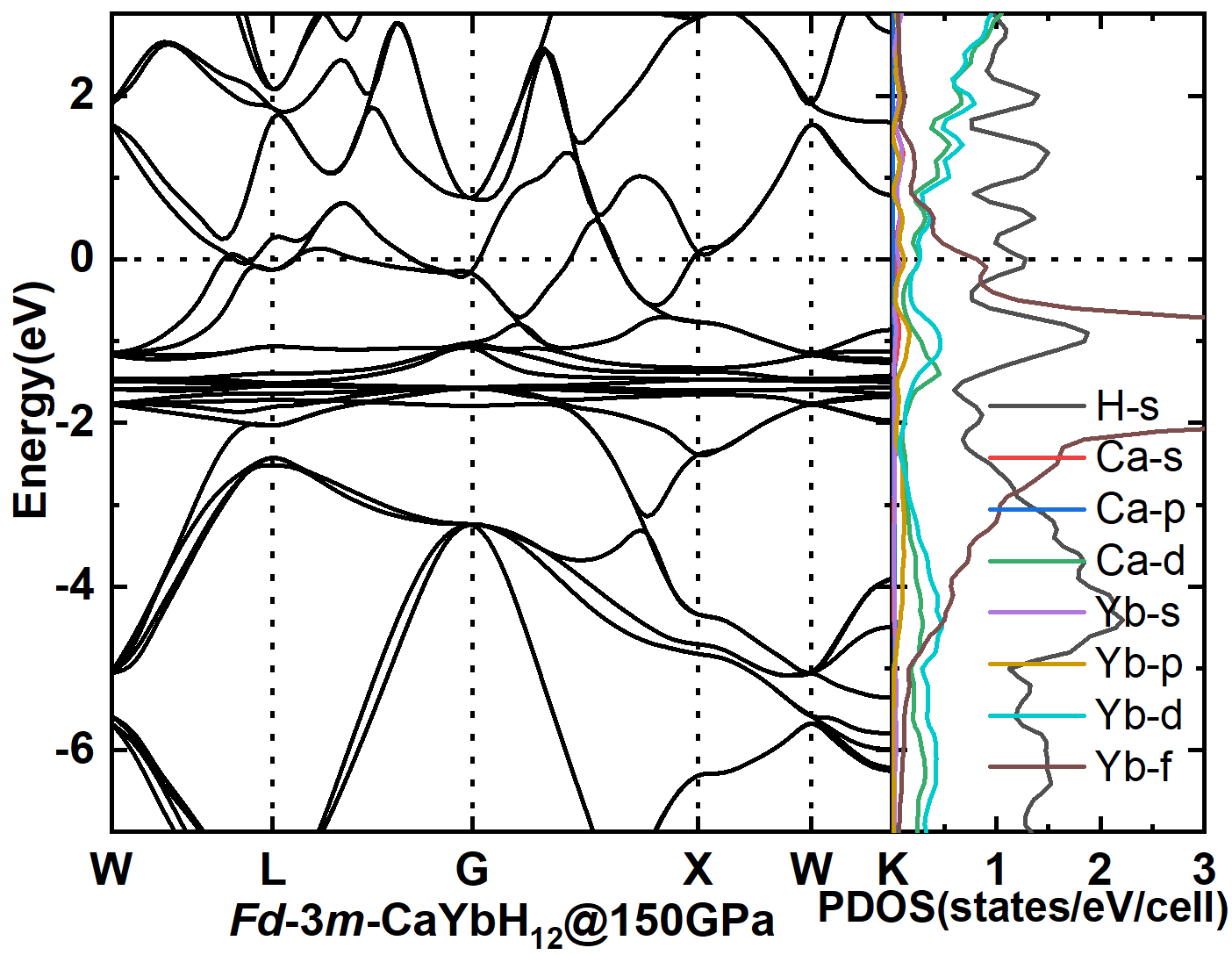


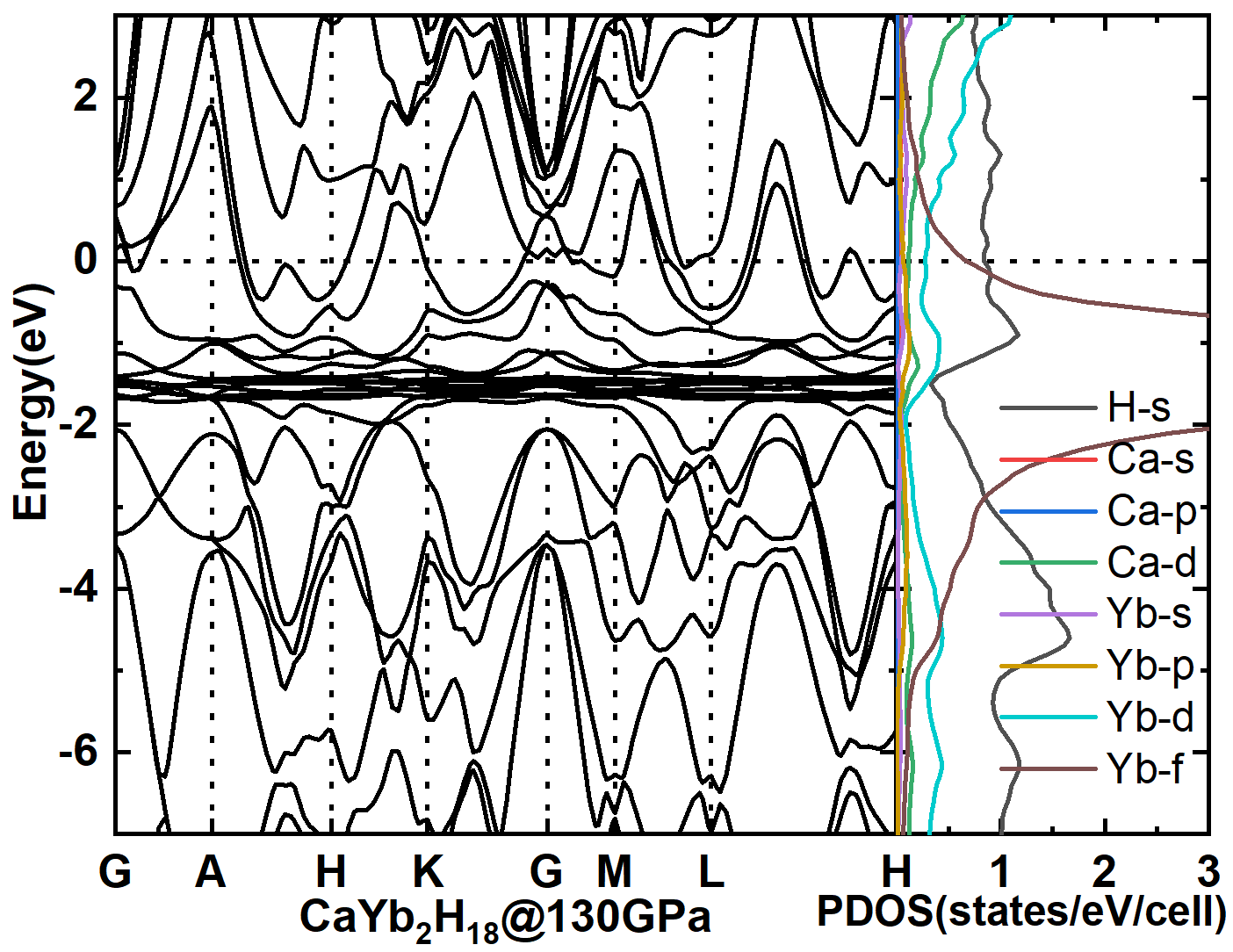

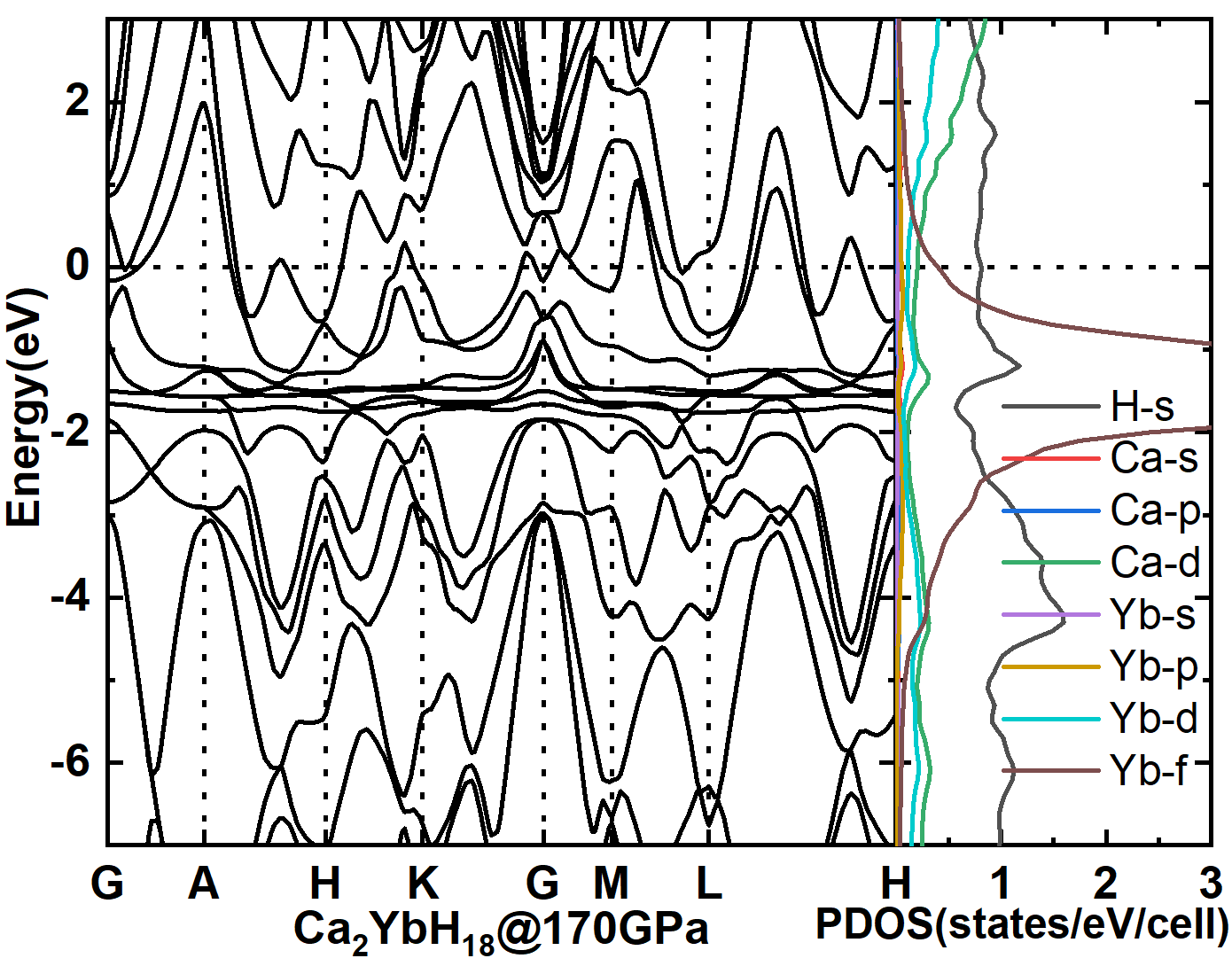


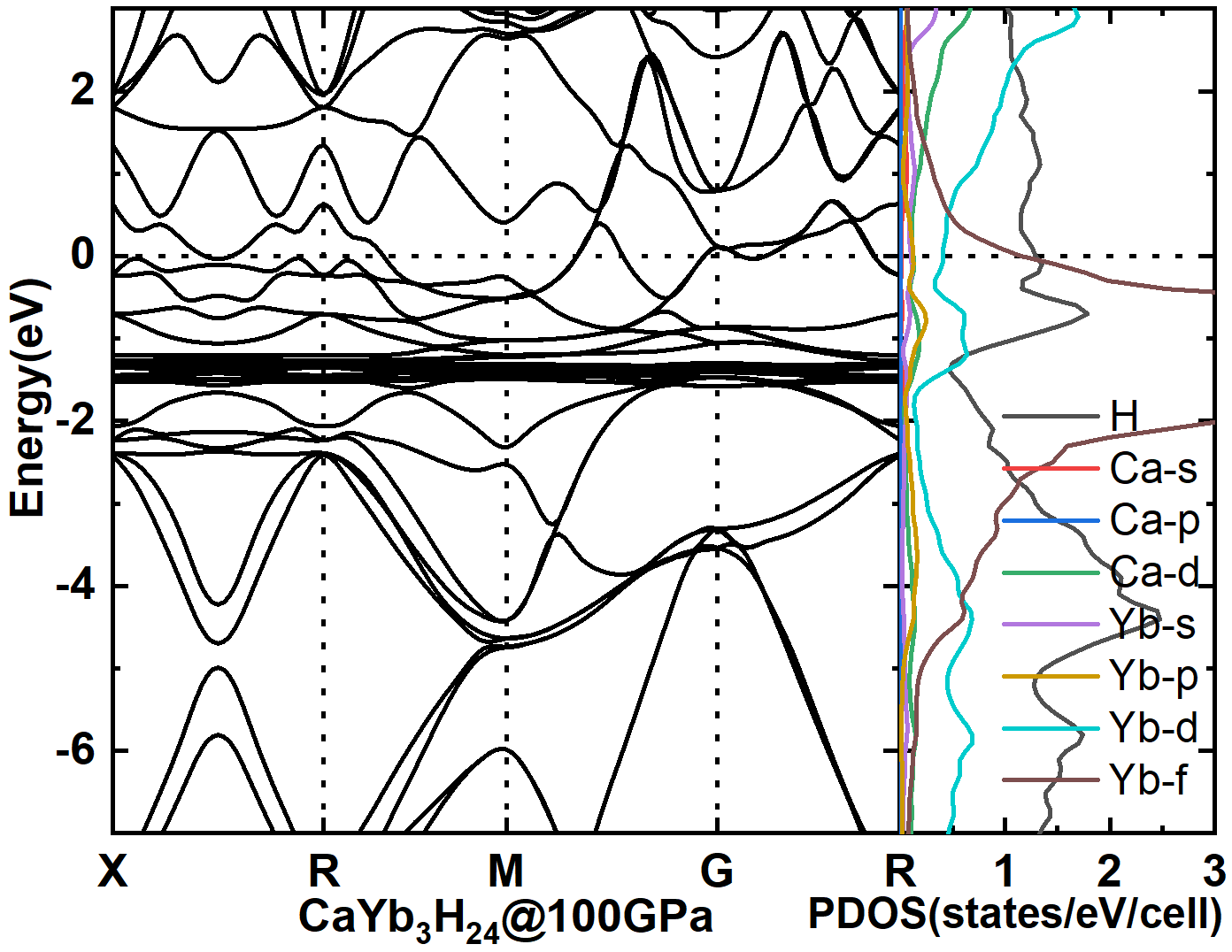

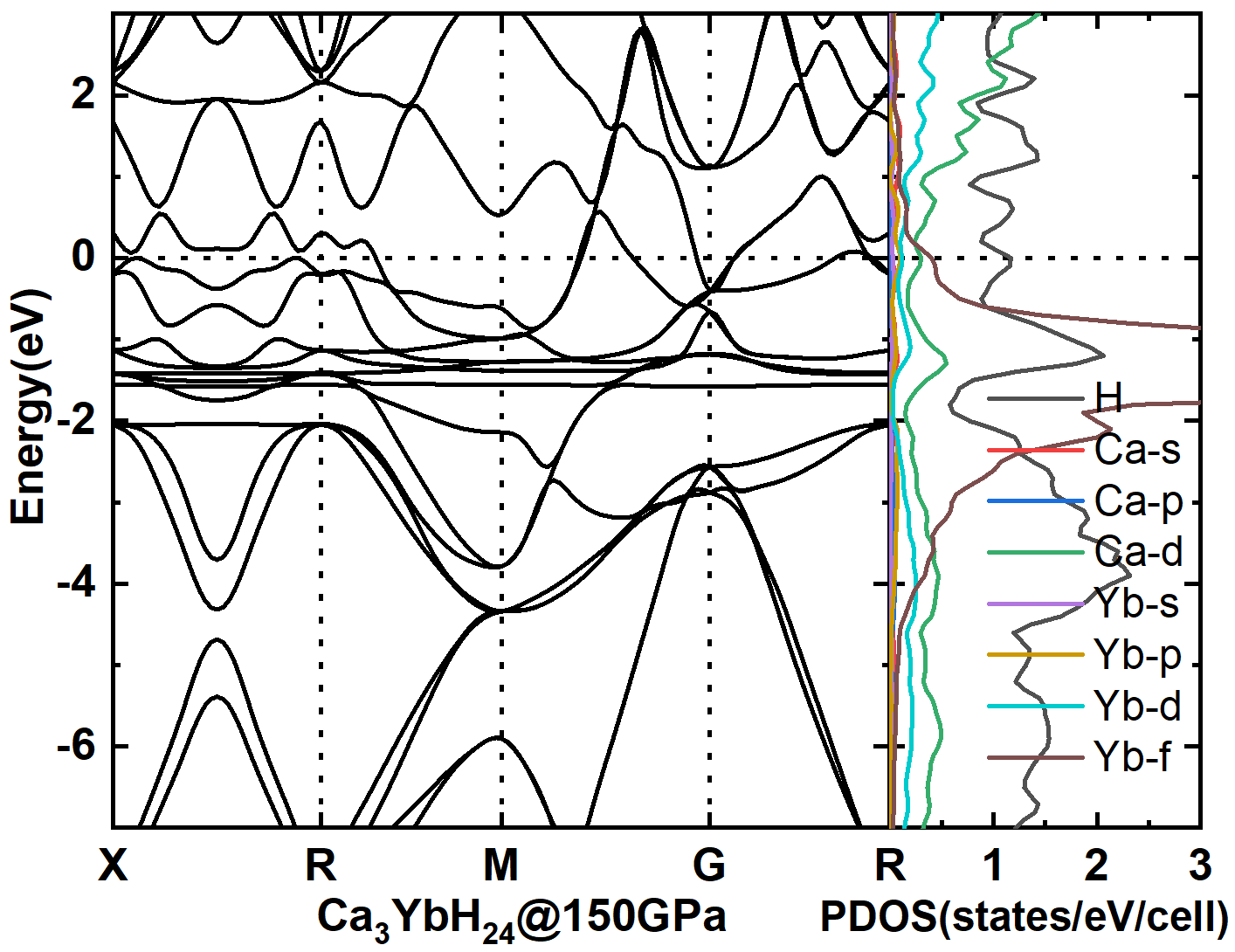


Fig. S20 Electronic band structures and projected density of electronic states of *Pm*-3*m*-CaYbH_12_, *Fd*-3*m*-CaYbH_12_, CaYb_2_H_18_, Ca_2_YbH_18_, CaYb_3_H_24_ and Ca_3_YbH_24_ under their minimum dynamically stable pressures, respectively.


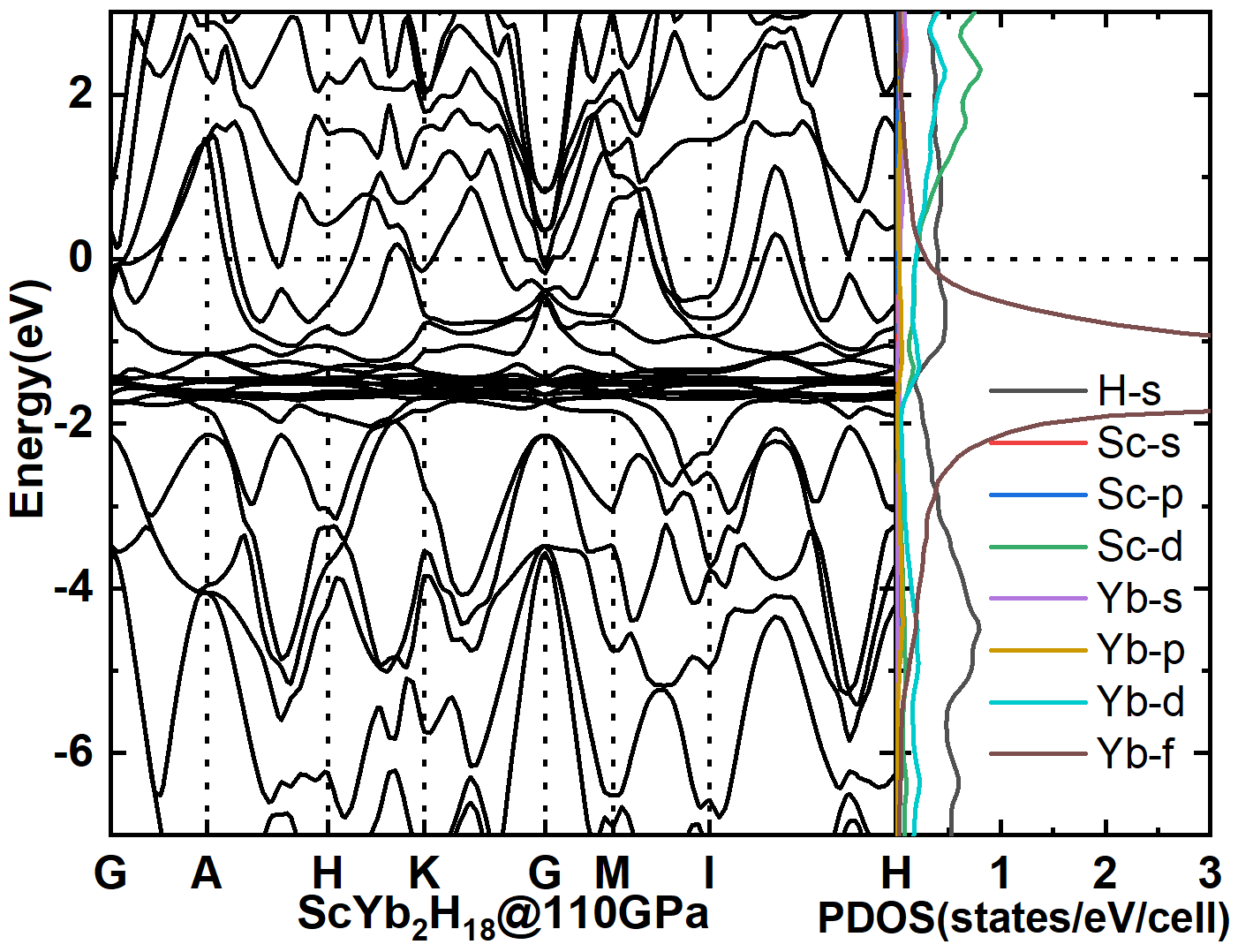

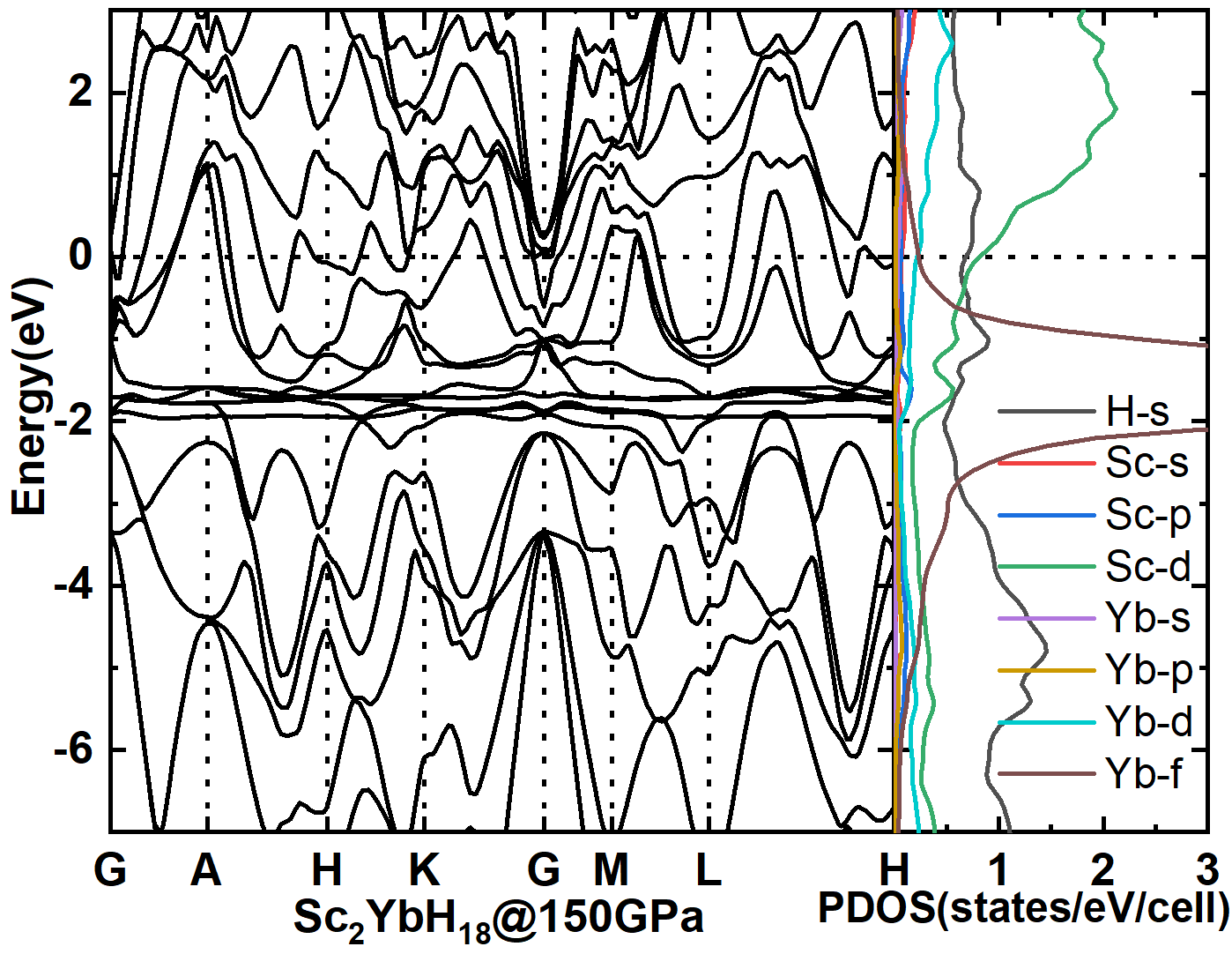


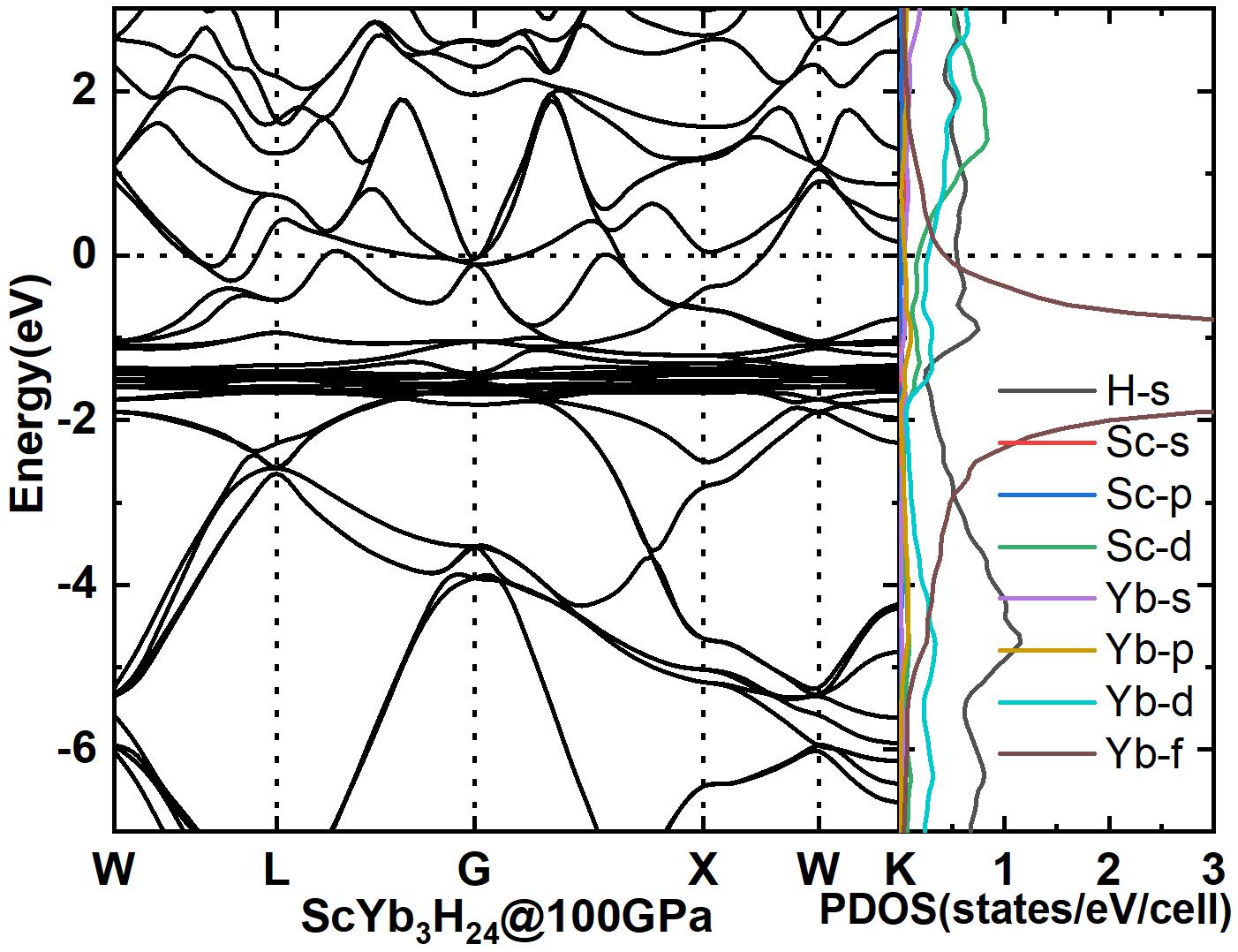

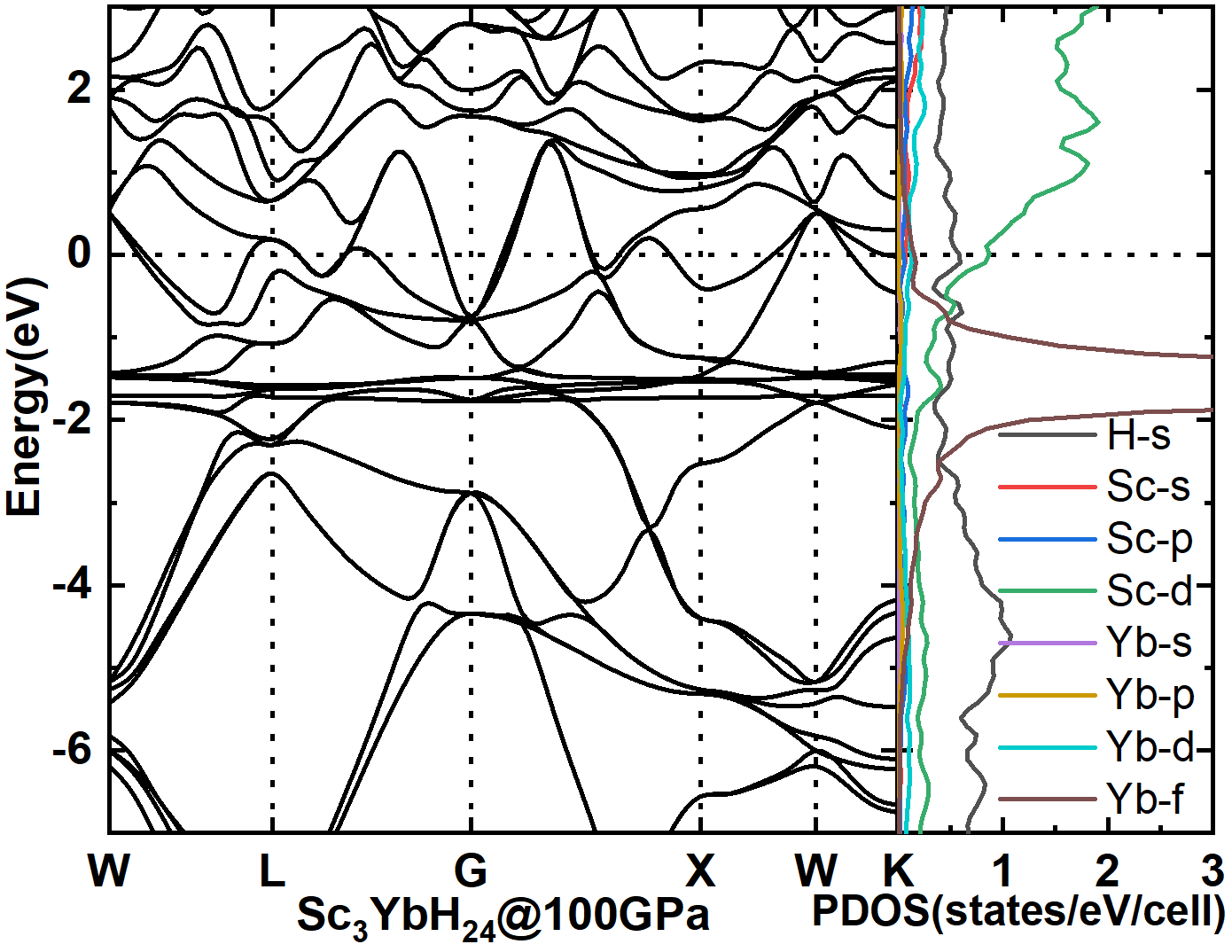


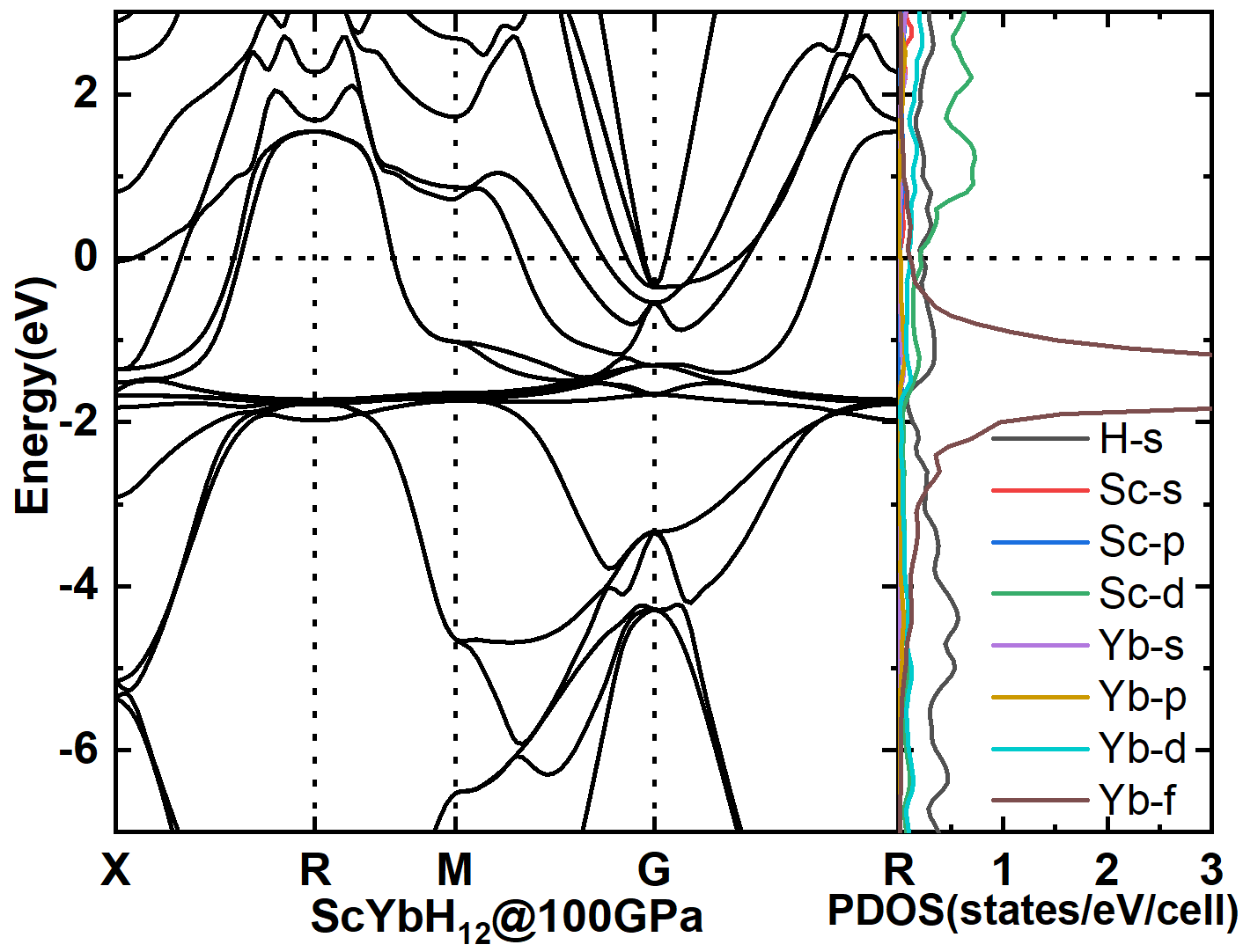


Fig. S21 Electronic band structures and projected density of electronic states of ScYb_2_H_18_, Sc_2_YbH_18_, ScYb_3_H_24_, Sc_3_YbH_24_ and ScYbH_12_ under their minimum dynamically stable pressures, respectively.


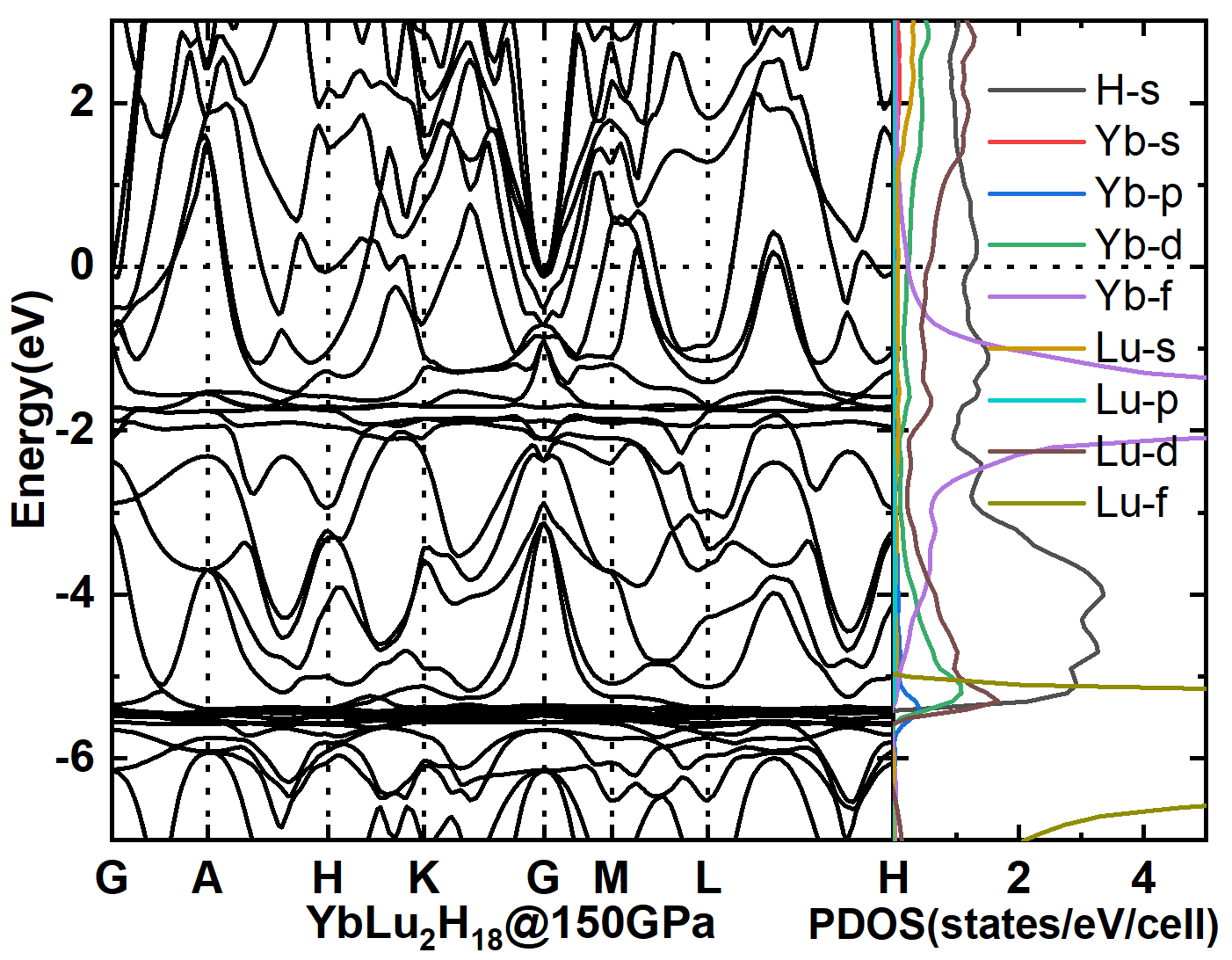

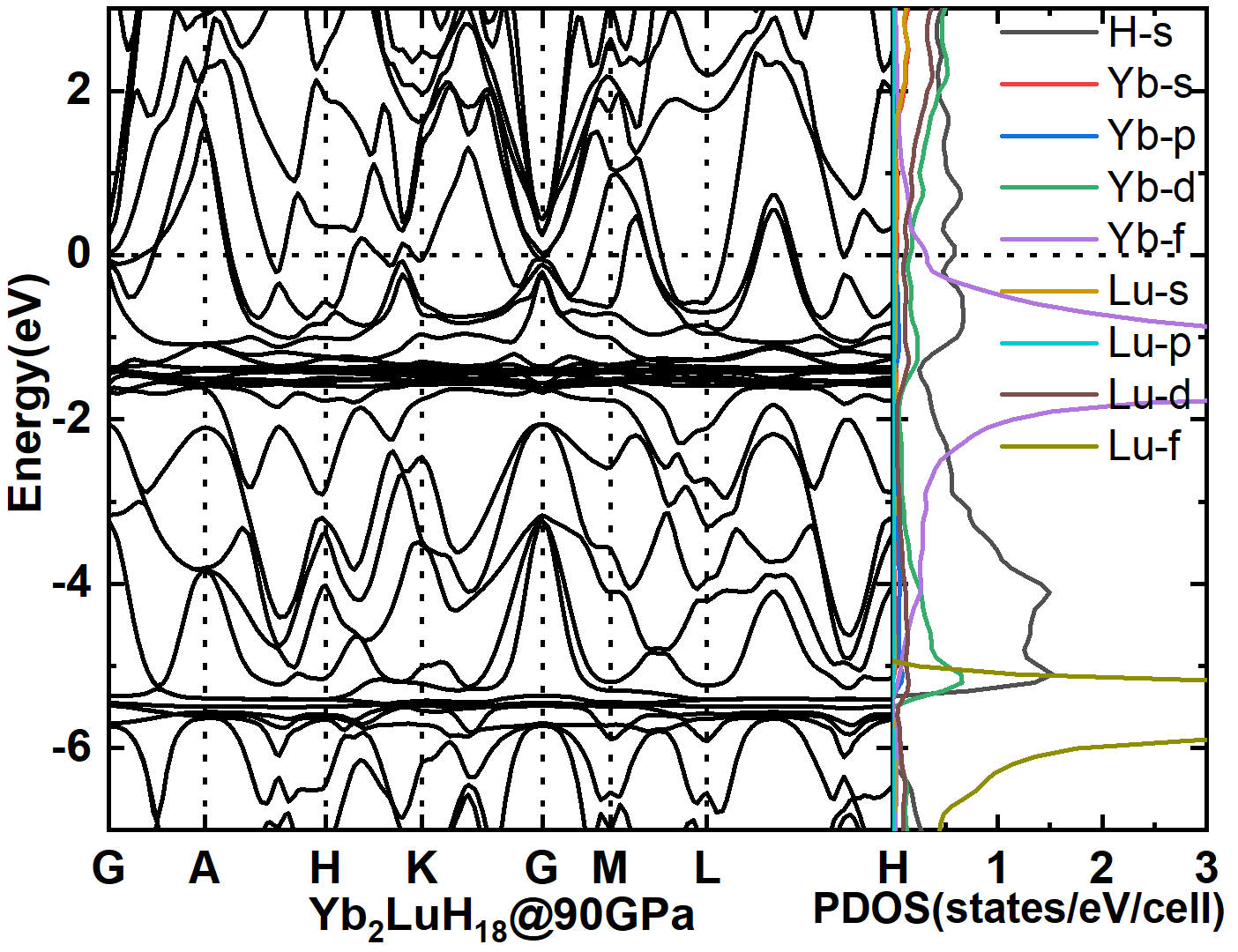


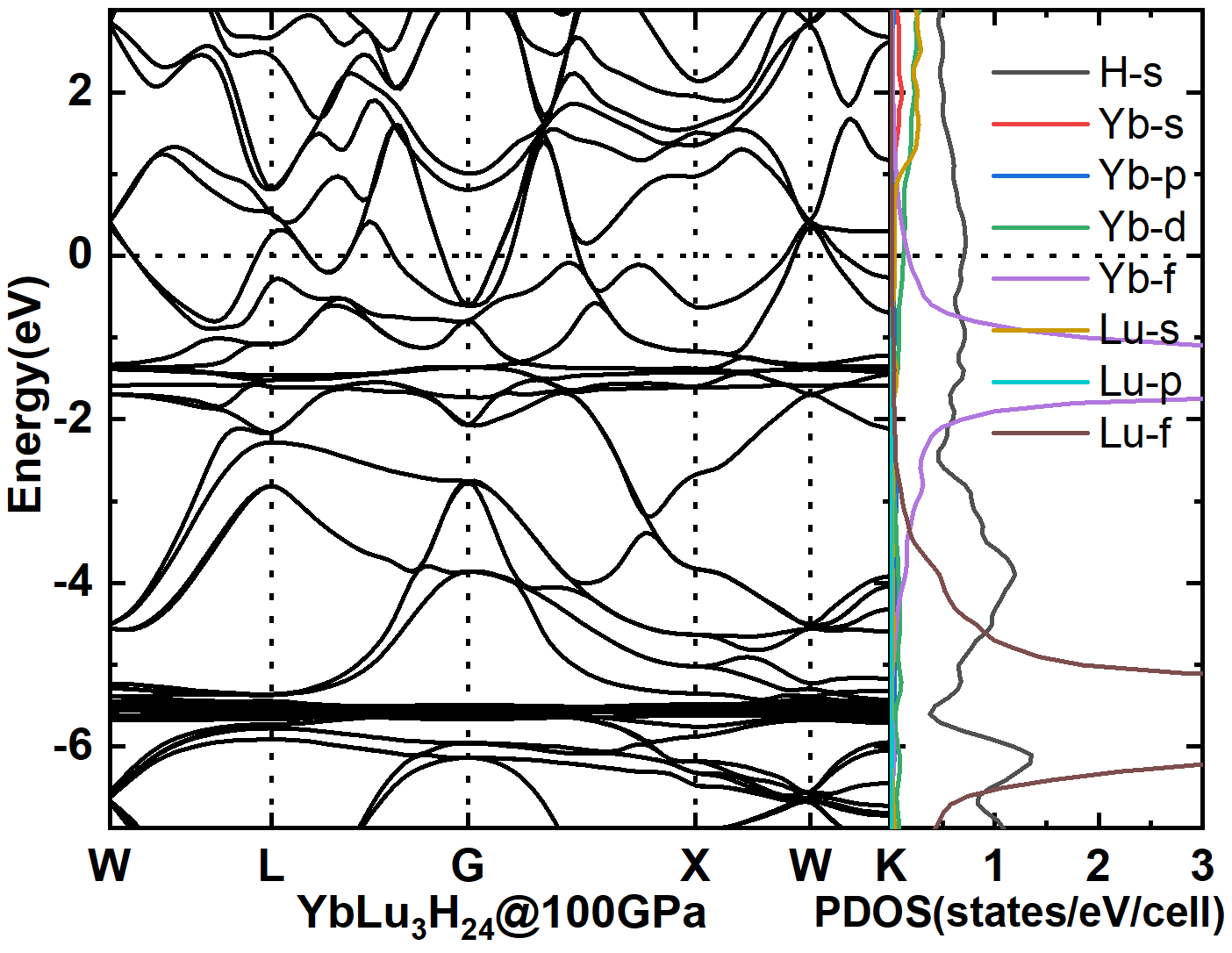

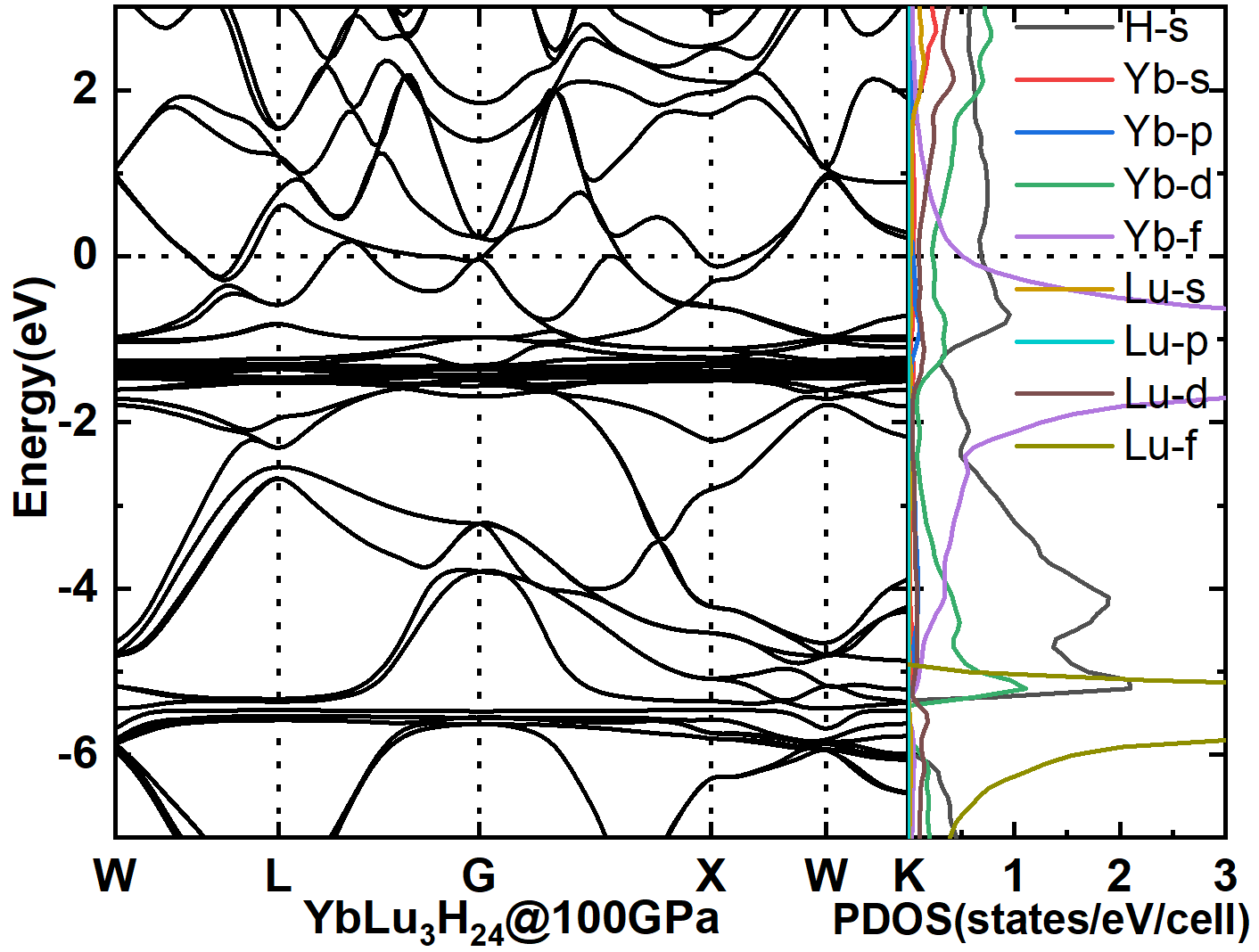


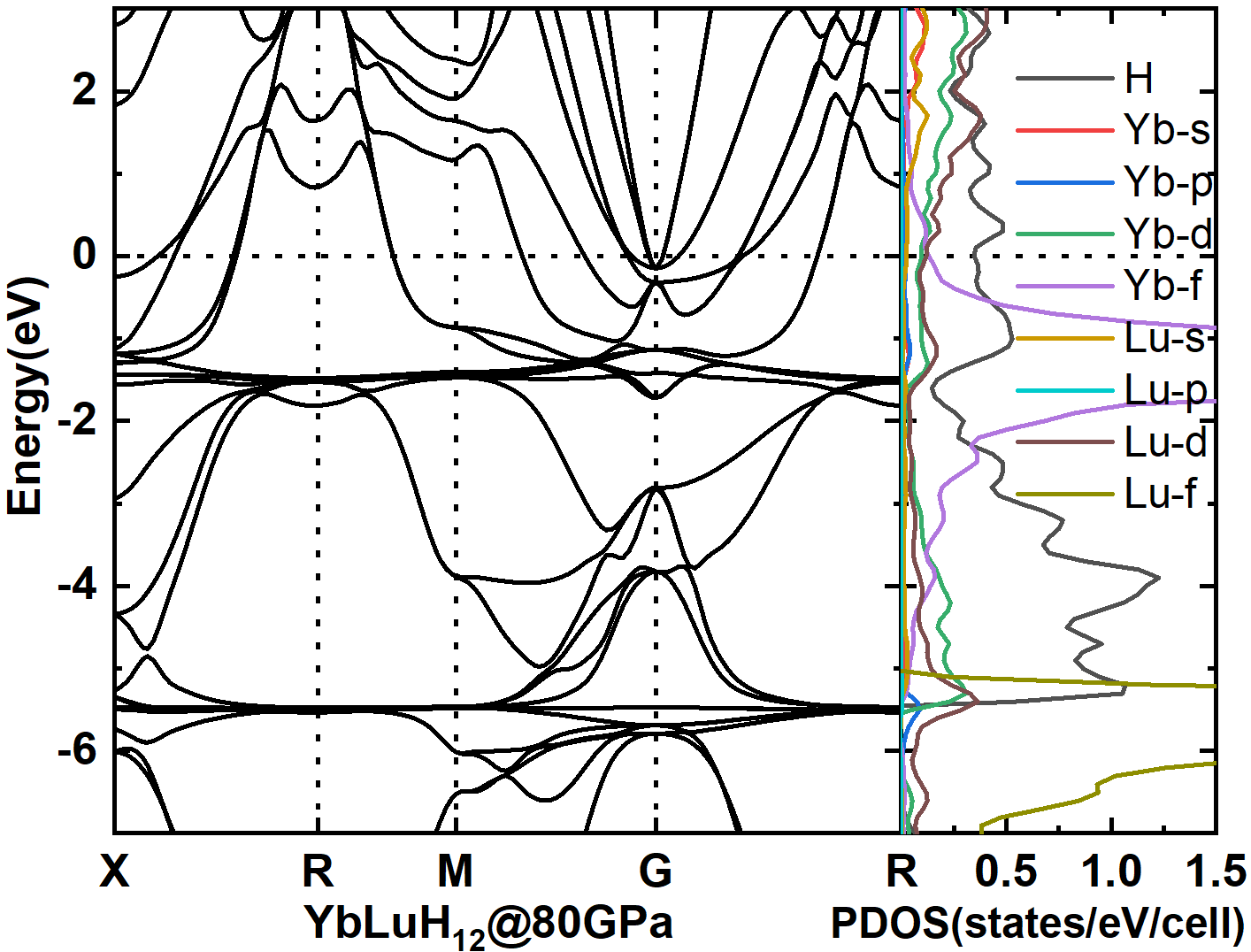


Fig. S22 Electronic band structures and projected density of electronic states of YbLu_2_H_18_, Yb_2_LuH_18_, YbLu_3_H_24_, Yb_3_LuH_24_ and YbLuH_12_ under their minimum dynamically stable pressures, respectively.

# Equations for calculating T_c_ and related parameters

**(1) The Allen−Dynes-modified McMillan equation**

T_c_ can be estimated by the McMillan equation^[6]^:

$T_{c}=\frac{\omega_{log}}{1.2}exp\left[ -\frac{1.04\left( 1+\lambda\right)}{\lambda-\mu^{*}\left( 1+0.62\lambda\right)} \right]$ (1)

where λ and ω_log_ are the electron−phonon coupling constant and the logarithmic-averaged phonon frequency, respectively, and μ^*^ is the Coulomb pseudopotential, for which we use the widely accepted range of 0.1-0.13. λ and ω_log_ are given by

$\lambda=2\int_{0}^{\infty} \frac{\alpha^{2}F(\omega)}{\omega}d\omega$ (2)

and

$\omega_{log}=exp\left( \frac{2}{\lambda}\int_{0}^{\infty} \frac{d\omega}{\omega}\alpha^{2}F(\omega)\ln\omega\right)$ (3)

The parameter ω denotes the phonon frequency, and α^2^F(ω) is the Eliashberg spectral function

$\alpha^{2}F\left( \omega\right)=\frac{1}{2\pi N(\varepsilon_{F})}\sum_{\boldsymbol{q}\upsilon} \frac{\gamma_{\boldsymbol{q}\upsilon}}{\omega_{\boldsymbol{q}\upsilon}}\delta(\omega-\omega_{\boldsymbol{q}\upsilon})$ (4)

The line width γ_q,υ_ is written as

$\gamma_{q\upsilon}=\pi\omega_{q\upsilon}\sum_{mn} \sum_{k} \left| g_{mn}^{\nu}\left( \boldsymbol{k},\boldsymbol{q} \right) \right|^{2}\delta(\varepsilon_{m,\boldsymbol{k}+\boldsymbol{q}}-\varepsilon_{F})\times\delta(\varepsilon_{n,\boldsymbol{k}}-\varepsilon_{F})$ (5)

where ε_n,_**_k_** is the energy of the bare electronic Bloch state, ε_F_ is the Fermi energy, and $g_{mn}^{\nu}\left( \boldsymbol{k},\boldsymbol{q} \right)$ is the electron−phonon matrix element.

When the value of $\lambda$ larger than 1.3, strong-coupling corrections begin to appear. Therefore, P. B. Allen and R. C. Dynes use two separate correction factors ($f_{1}$ and $f_{2}$) to describe these two effects. Then we can further obtain the Allen−Dynes-modified McMillan equation^[7]^:

$T_{c}=\frac{f_{1}f_{2}\omega_{log}}{1.2}exp\left[ -\frac{1.04\left( 1+\lambda\right)}{\lambda-\mu^{*}\left( 1+0.62\lambda\right)} \right]$ (6)

$f_{1}$ and $f_{2}$ are given by

$f_{1}=\sqrt[3]{\left[ 1+\left( \frac{\lambda}{2.46(1+3.8\mu^{*})} \right)^{\frac{3}{2}} \right]}$ (7)

and

$f_{2}=1+\frac{\left( \frac{\omega_{2}}{\omega_{log}}-1 \right)\lambda^{2}}{\lambda^{2}+\left[ 1.82(1+6.3\mu^{*})\frac{\bar{\omega}_{2}}{\omega_{log}} \right]}$ (8)

average frequencies $\bar{\omega}_{2}$ is given by

$\bar{\omega}_{2}=\sqrt{\frac{2}{\lambda}}\int_{0}^{\infty} \frac{d\omega}{\omega}\alpha^{2}F(\omega)\omega d\omega$ (9)

**(2) Self-consistent solution of the Eliashberg equation**

For strong-coupling system, it can be better described with Eliashberg equation^[8]^:

$Z\left( i\omega_{n} \right)\Delta\left( i\omega_{n} \right)=\frac{\pi T}{N_{F}}\sum_{n^{'}} \frac{\Delta\left( i\omega_{n}^{'} \right)}{\sqrt{\omega_{n}^{'2}+\Delta^{2}\left( i\omega_{n}^{'} \right)}}\times[\lambda(\omega_{n}-\omega_{n^{'}})-N_{F}\mu^{*}]\delta(\epsilon)$ (10)

$\begin{aligned} Z(i\omega_{n})=1+\frac{\pi T}{N_{F}\omega_{n}}\sum_{n^{'}} \frac{\omega_{n}^{'}}{\sqrt{\omega_{n}^{'2}+\Delta^{2}\left( i\omega_{n}^{'} \right)}}\lambda\left( \omega_{n}-\omega_{n^{'}} \right)\delta\left( \epsilon\right) \end{aligned}$ (11)

where functions $Z(i\omega_{n})$ and $\Delta\left( i\omega_{n} \right)$ are the renormalization function and pairing order parameter, respectively. $N_{F}$ is the density of electronic states at the Fermi level, and $\delta(\epsilon)$ is the Dirac delta function. ${i\omega}_{n}=i(2n+1)\pi T_{c}$ are the fermion Matsubara frequencies. $\mu^{*}$is the Coulomb pseudopotential, for which we use the widely accepted range of 0.1 - 0.13. $\lambda(\omega_{n}-\omega_{n^{'}})$ contains the electron-phonon coupling matrix, phonon propagator, and the phonon density of states, and is given by:

$\lambda(\omega_{n}-\omega_{n^{'}})=\int_{0}^{\infty} d\omega\frac{2\omega}{(\omega_{n}-\omega_{n}^{'})^{2}+\omega^{2}}\alpha^{2}F(\omega)$ (12)

The equations for the $Z(i\omega_{n})$ and $\Delta\left( i\omega_{n} \right)$ form a coupled nonlinear system and are solved self-consistently. We evaluated renormalization function and the order parameter for each Matsubara frequency along the imaginary energy axis. After calculating $Z(i\omega_{n})$ and $\Delta\left( i\omega_{n} \right)$, an analytic continuation is performed to the real axis using Pade’ functions.

The specific process is as follows:


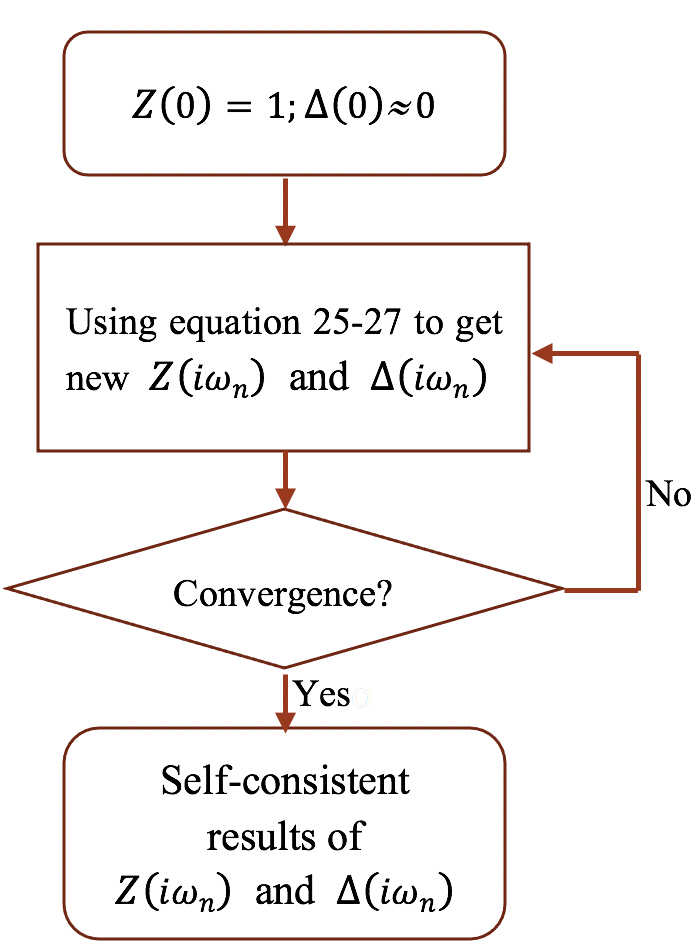


# TABLES

Table S1. The calculated electron-phonon coupling parameter λ, logarithmic average phonon frequency ω_log_, superconducting critical temperature f_1_f_2_*T_c_* using Allen-Dynes modified McMillan equation and *T_c_*^scE^ using the self-consistent solution of the Eliashberg equation for dynamically stable superconductors found in this work. The pressure presented here is where they start to become dynamically stable. The Coulomb pseudopotential μ^∗^ were chose 0.10 and 0.13.

| Compound | Phase | Pressure  (GPa) | λ | ω_log_ (K) | f_1_f_2_T_c_ (K) | T_c_ ^scE^ (K) |
| --- | --- | --- | --- | --- | --- | --- |
| Y_3_LuH_24_ | *Fm*-3*m* | 120 | 3.75 | 696 | 199-218 | 269-283 |
| Y_2_LuH_18_ | *P-*3*m*1 | 100 | 3.21 | 747 | 184-201 | 224-240 |
| YLuH_12_ | *Pm*-3*m* | 140 | 3.17 | 771 | 190-208 | 256-275 |
| YLu_2_H_18_ | *P-*3*m*1 | 100 | 3.24 | 723 | 180-197 | 226-242 |
| YLu_3_H_24_ | *Fm*-3*m* | 110 | 4.78 | 495 | 183-202 | 274-288 |
| Y_3_YbH_24_ | *Fm*-3*m* | 100 | 4.78 | 331 | 124-138 | 209-222 |
| Y_2_YbH_18_ | *P-*3*m*1 | 100 | 3.24 | 595 | 151-165 | 205-221 |
| YYbH_12_ | *Pm*-3*m* | 100 | 2.22 | 761 | 134-147 | 166-178 |
| YYb_2_H_18_ | *P-*3*m*1 | 110 | 1.76 | 946 | 131-144 | 160-172 |
| YYb_3_H_24_ | *Fm*-3*m* | 100 | 1.55 | 944 | 113-125 | 136-148 |
| Ca_3_LuH_24_ | *Fm*-3*m* | 170 | 2.64 | 816 | 169-184 | 206-221 |
| Ca_2_LuH_18_ | *P-*3*m*1 | 170 | 2.19 | 1087 | 188-205 | 216-232 |
| CaLuH_12_ | *Pm*-3*m* | 170 | 3.94 | 648 | 197-216 | 267-282 |
| CaLu_2_H_18_ | *P-*3*m*1 | 140 | 4.13 | 635 | 202-222 | 284-299 |
| CaLu_3_H_24_ | *Fm*-3*m* | 130 | 3.60 | 840 | 198-216 | 240-255 |
| Ca_3_YbH_24_ | *Fm*-3*m* | 150 | 3.09 | 371 | 94-104 | 183-196 |
| Ca_2_YbH_18_ | *P-*3*m*1 | 170 | 2.02 | 707 | 116-128 | 158-171 |
| CaYbH_12_ | *Pm*-3*m* | 130 | 3.30 | 551 | 131-144 | 165-176 |
| CaYbH_12_ | *Fd*-3*m* | 150 | 1.38 | 1140 | 118-132 | 140-153 |
| CaYb_2_H_18_ | *P-*3*m*1 | 130 | 1.65 | 835 | 107-119 | 125-138 |
| CaYb_3_H_24_ | *Fm*-3*m* | 100 | 1.91 | 782 | 118-129 | 141-151 |
| Sc_3_LuH_24_ | *Fm*-3*m* | 100 | 3.58 | 484 | 139-153 | 236-250 |
| Sc_2_LuH_18_ | *P-*3*m*1 | 110 | 3.33 | 535 | 142-156 | 222-239 |
| ScLuH_12_ | *Pm*-3*m* | 100 | 4.43 | 517 | 175-193 | 253-266 |
| ScLu_2_H_18_ | *P-*3*m*1 | 100 | 3.66 | 489 | 143-157 | 233-247 |
| ScLu_3_H_24_ | *Fm*-3*m* | 100 | 2.01 | 501 | 173-191 | 258-271 |
| Sc_3_YbH_24_ | *Fm*-3*m* | 100 | 2.60 | 603 | 127-139 | 190-203 |
| Sc_2_YbH_18_ | *P-*3*m*1 | 150 | 1.94 | 921 | 142-156 | 182-196 |
| ScYbH_12_ | *Pm*-3*m* | 100 | 2.10 | 778 | 130-143 | 177-191 |
| ScYb_2_H_18_ | *P-*3*m*1 | 110 | 1.68 | 886 | 117-129 | 147-160 |
| ScYb_3_H_24_ | *Fm*-3*m* | 100 | 1.65 | 846 | 110-121 | 138-150 |
| Yb_3_LuH_24_ | *Fm*-3*m* | 80 | 1.97 | 787 | 123-135 | 151-162 |
| Yb_2_LuH_18_ | *P-*3*m*1 | 90 | 2.14 | 789 | 133-146 | 170-182 |
| YbLuH_12_ | *Pm*-3*m* | 80 | 2.82 | 636 | 140-153 | 186-198 |
| YbLu_2_H_18_ | *P-*3*m*1 | 150 | 1.77 | 1073 | 150-166 | 197-212 |
| YbLu_3_H_24_ | *Fm*-3*m* | 100 | 2.65 | 833 | 171-187 | 209-222 |

Table S2. The calculated electron-phonon coupling parameter λ, logarithmic average phonon frequency ω_log_, superconducting critical temperature f_1_f_2_*T_c_* using Allen-Dynes modified McMillan equation and *T_c_*^scE^ using the self-consistent solution of the Eliashberg equation for CaH_6_, YH_6_ and ScH_6_. The Coulomb pseudopotential μ^∗^ were chose 0.10 and 0.13.

| Compound | Phase | Pressure  (GPa) | λ | ω_log_ (K) | f_1_f_2_T_c_ (K) | T_c_ ^scE^ (K) |
| --- | --- | --- | --- | --- | --- | --- |
| CaH_6_ | *Im*-3*m* | 150 | 2.46 | 1050 | 195-212 | 220-233 |
| CaH_6_ | *Im*-3*m* | 172 | 2.06 | 1167 | 186-203 | 209-223 |
| CaH_6_ | *Im*-3*m* | 172 | exp^[9]^ | | 215 | |
| YH_6_ | *Im*-3*m* | 120 | 2.77 | 866 | 186-203 | 232-247 |
| YH_6_ | *Im*-3*m* | 166 | 1.98 | 1154 | 180-198 | 218-234 |
| YH_6_ | *Im*-3*m* | 166 | exp^[10]^ | | 224 | |
| ScH_6_ | *Im*-3*m* | 130 | 2.26 | 752 | 137-150 | 189-203 |

Table S3 The lattice parameters and atomic positions of YLuH_12_, YLu_2_H_18_, Y_2_LuH_18_, YLu_3_H_24_ and Y_3_LuH_24_ under different pressures.

| **Structure** | **Parameters**  (Å, deg) | **Atom** | **x** | **y** | **z** |
| --- | --- | --- | --- | --- | --- |
| *Pm*-3*m*  YLuH_12_  (140 GPa) | a=b=c= 3.6094  α=β=γ= 90 | H  Y  Lu | 0.25322  0.50000  0.00000 | 0.00000  0.50000  0.00000 | 0.50000  0.50000  0.00000 |
| *Fd*-3*m*  YLuH_12_  (200 GPa) | a=b=c= 4.9396  α=β=γ= 60 | H  Y  Lu | 1.37371  1.75000  1.00000 | 0.12500  -0.25000  0.00000 | -0.37500  -0.25000  0.00000 |
| *P*-3*m*1  YLu_2_H_18_  (100 GPa) | a=b= 5.2419  c= 3.2088  α=β= 90  γ= 120 | H  H  Y  Lu | 0.08731  0.25752  0.00000  0.66667 | 0.66858  0.25752  0.00000  0.33333 | 0.84076  0.50000  0.00000  0.65774 |
| *P*-3*m*1  Y_2_LuH_18_  (100 GPa) | a=b= 5.2633  c= 3.2244  α=β= 90  γ= 120 | H  H  Y  Lu | 0.58588  0.24566  0.66667  0.00000 | 0.66623  0.24566  0.33333  0.00000 | 0.66995  0.00000  0.83002  0.50000 |
| *Fm*-3*m*  YLu_3_H_24_  (110 GPa) | a=b=c= 5.1959  α=β=γ= 60 | H  Y  Lu  Lu | -0.87486  -0.50000  -0.25000  0.00000 | 1.87486  1.50000  0.75000  0.00000 | -0.37363  -0.50000  -0.25000  0.00000 |
| *Fm*-3*m*  Y_3_LuH_24_  (120 GPa) | a=b=c= 5.1894  α=β=γ= 60 | H  Y  Y  Lu | 1.87502  0.00000  2.25000  1.50000 | -0.62340  0.00000  -0.75000  -0.50000 | -0.87502  0.00000  -0.75000  -0.50000 |

**References**

[1] Liu HY, Naumov, II, Hoffmann R, et al. Potential high-t-c superconducting lanthanum and yttrium hydrides at high pressure. Proc Natl Acad Sci U S A, 2017, 114: 6990-6995

[2] Peng F, Sun Y, Pickard CJ, et al. Hydrogen clathrate structures in rare earth hydrides at high pressures: Possible route to room-temperature superconductivity. Phys Rev Lett, 2017, 119: 6

[3] Hao Song ZZ, Tian Cui, Chris J. Pickard, Vladimir Z. Kresin,, Duan D. High tc superconductivity in heavy rare earth hydrides. Chinese Physics Letters, 2021, 38: 107401

[4] Wang H, Tse JS, Tanaka K, et al. Superconductive sodalite-like clathrate calcium hydride at high pressures. Proc Natl Acad Sci U S A, 2012, 109: 6463

[5] Abe K. Hydrogen-rich scandium compounds at high pressures. Phys Rev B, 2017, 96: 144108

[6] McMillan WL. Transition temperature of strong-coupled superconductors. Phys Rev, 1968, 167: 331

[7] Allen PB, Dynes RC. Transition temperature of strong-coupled superconductors reanalyzed. Phys Rev B, 1975, 12: 905-922

[8] Eliashberg GM. Interactions between electrons and lattice vibrations in a superconductor. Sov Phys Jetp, 1960, 11:3: 696-702

[9] Ma L, Wang K, Xie Y, et al. Experimental observation of superconductivity at 215 k in calcium superhydride under high pressures. In: Proceedings of the 2021.

[10] Troyan IA, Semenok DV, Kvashnin AG, et al. Anomalous high-temperature superconductivity in yh6. Adv Mater, 2021, 33: 2006832
